# Supplementary material for: Unravelling an oxygen-mediated reductive quenching pathway for photopolymerisation under long wavelengths
Source: Nat Commun. 2021 Jan 20;12:478. doi: 10.1038/s41467-020-20640-z (PMC7817663; doi:10.1038/s41467-020-20640-z)
Supplement: Supplementary file 1 — Supplementary Information [file 41467_2020_20640_MOESM1_ESM.pdf]

Supplementary Information

Unravelling an Oxygen-Mediated Reductive Quenching Pathway for Photopolymerisation under Long Wavelengths

Chenyu Wu,[1,2,3] Kenward Jung,[1,2] Yongtao Ma,[3] Wenjian Liu[3*] and Cyrille Boyer[1,2*]

[1]*Centre for Advanced Macromolecular Design (CAMD), School of Chemical Engineering, UNSW Sydney, NSW 2052, Australia. Correspondence to Prof Cyrille Boyer; email: cboyer@unsw.edu.au*

[2]*Australian Centre for NanoMedicine, UNSW Sydney, NSW 2052, Australia. Correspondence to Prof Cyrille Boyer; email: cboyer@unsw.edu.au*

[3]*Qingdao Institute for Theoretical and Computational Sciences, Shandong University, Qingdao 266237, China. Correspondence to Prof Wenjian Liu; email: liuwj@sdu.edu.cn*

Contents

Supplementary Note 1. Materials 4

Supplementary Note 2. Instrumentation 4

Supplementary Note 3. PET-RAFT polymerisation preparation 6

3.1 Selecting the model system 6

3.2 Selecting irradiation conditions 7

3.3 General procedures for dye-catalysed PET-RAFT polymerisation 7

Supplementary Note 4. Characterisation of synthesised polymers 12

4.1 Molecular weight distribution characterisation 12

4.2 Polymer purification 20

4.3 Analysis by NMR spectroscopy 20

4.4 MALDI-TOF MS analysis 22

4.5 End-group fidelity confirmation by chain-extension experiments 23

Supplementary Note 5. Evaluation of photophysical structure-property relationships 32

5.1 Photophysical processes 32

5.2 Structure-property relationships for porphyrin dyes 33

5.3 Structure-property relationships for tetraaza-porphyrin and phthalocyanine dyes 37

Supplementary Note 6. Thermodynamic studies on tetraaza-porphyrin and phthalocyanine dyes. 39

Supplementary Note 7. Electron transfer or energy transfer? 41

7.1 Radiative energy transfer 41

7.2 Förster resonance energy transfer 42

7.3 Dexter energy transfer 42

7.4 Proof from actual systems mechanistically investigated in our previous work 44

7.5 Photoinduced electron transfer and conclusion 44

Supplementary Note 8. Deriving thermodynamic thresholds for SET processes 45

8.1 Characteristics of SET processes 45

8.2 Deriving thermodynamic thresholds for GS-GS SET processes 45

8.3 Deriving thermodynamic thresholds for ES-GS SET (PET) processes 47

Supplementary Note 9. Experimental detection of $^1O_2$ and evidence of $^1O_2$-TEA reaction 48

Supplementary Note 10. Additional discussion of O-RQP-II 51

Supplementary Note 11. Expanded scope of monomers, RAFT agents and solvents in O-RQP ... 52

Supplementary Note 12. Excluding the effect of alkyl groups on phthalocyanine 55

Supplementary Note 13. DFT calculations 56

13.1 Calculation for molecular geometries and energies 56

13.2 Molecular orbital transition and wave function analysis 113

13.3 Calculating excited state energies with ZnOEP as an example 114

13.4 Thermodynamics calculations 118

13.5 Probing into the $O_2^{\bullet -}$-DTPA pair 119

Supplementary Note 14. Chain of evidence summarised for the O-RQP scheme 122

Supplementary References 123

Supplementary Note 1. Materials

Methyl acrylate (MA, 99%), benzyl acrylate (BzA, 99.8%), di(ethylene glycol) ethyl ether acrylate (DEGEEA, ≥ 90%), dimethylacrylamide (DMA, 99%), diethylacrylamide (DEA, 99%), 4-acryloylmorpholine (NAM, 97%) and methyl methacrylate (MMA, 99%) were purchased from Sigma-Aldrich as the model monomer and de-inhibited by percolation over a plug of basic alumina (Ajax Chemical, AR) before use. 2-(dodecylthiocarbonothioylthio)propionic acid (DTPA, 97%) was purchased from Sigma-Aldrich as the model chain transfer agent (CTA) for photoinduced electron transfer reversible addition-fragmentation chain transfer (PET-RAFT) polymerisation via different quenching pathways. In addition, 4-cyano-4-[(dodecylsulfanylthiocarbonyl)sulfanyl]pentanoic acid (CDTPA, 97%) was selected as an alternative CTA for polymerisation of more monomers. Triethylamine (TEA, 99.5%) was purchased from Sigma-Aldrich as the model reducing agent co-catalyst. Dimethyl sulfoxide (DMSO), *N*,*N*'-dimethylacetamide (DMAc), *N*,*N*'-dimethylformamide (DMF), acetonitrile (MeCN), methanol (MeOH), ethyl acetate (EtOAc), tetrahydrofuran (THF), acetone, dioxane, diethyl ether and petroleum spirit were purchased from Ajax Chemical and used as received. Zinc(II) 2,3,7,8,12,13,17,18-octaethyl-porphyrin (ZnOEP, 97%), zinc(II) 5,10,15,20-tetraphenyl-porphyrin (ZnTPP, 97.5%), 5,10,15,20-tetraphenyl-21H,23H-porphyrin (TPP, 99%), zinc (II) 2,7,12,17-tetra-tert-butyl-5,10,15,20-tetraaza-porphine (ZnTtBAzP, 85%), zinc (II) 2,9,16,23-tetra-tert-butyl-phthalocyanine (ZnTtBPC, 96%), zinc (II) phthalocyanine (ZnPC, 97%), zinc (II) 1,4,8,11,15,18,22,25-octabutoxy-phthalocyanine (ZnOBOPC, 95%), zinc (II) 2,11,20,29-tetra-tert-butyl-2,3-naphthalocyanine (ZnTtBNPC, 90%) were purchased from Sigma-Aldrich. zinc(II) 5,10,15,20-tetra-(trimethylsilylethynyl)-porphyrin (ZnTSP, 98%) was purchased from PorphyChem SAS, France.

Supplementary Note 2. Instrumentation

Irradiation setup for PET-RAFT polymerisation. Glass cuvettes (6G) dimensioned 1 cm × 2 mm × 5 cm, capped with a rubber septum and sealed with Parafilm, were used for irradiation in the model

PET-RAFT polymerisation. Thorlabs Mounted Light Emitting Diodes (LEDs) with nominal wavelengths of 565 nm, 590 nm, 625 nm, 660 nm, 730 nm and 780 nm were used as the irradiation light sources. The irradiation wavelength was chosen based on the maximum absorption wavelength λ_{max} corresponding to the lowest-energy-excitation peak (right-most absorption peak in the Q-band) of the photocatalyst (PC) used in the PET-RAFT polymerisations. The light intensity was set to 10 mW/cm^2 and the temperature was kept constant as 25 °C (room temperature, RT) in all cases.

Online Fourier transform near-infrared (FTNIR) spectroscopy. FTNIR spectra were recorded to monitor the monomer signal of the vinylic C-H stretching overtone at ~6200 cm^{-1}. Monomer conversions were calculated by the decrease of the integration of vinylic C-H peak divided by the initial value. The Brucker IFS 66/S Fourier transform spectrometer for recording FTNIR spectra was equipped with a tungsten halogen lamp, a CaF_2 beam splitter and liquid-nitrogen-cooled InSb detector. After irradiation of every specified time interval (10 min or 20 min depending on the polymerisation rate) in the course of polymerisation, the sample was manually placed in the sample holder for FTNIR scanning and each spectrum composed of 16 scans with a resolution of 4 cm^{-1} (15 s per spectrum). Peak analysis was performed in OPUS software.

UV-Vis spectroscopy. UV-vis spectra were recorded by a Varian CARY 300 spectrophotometer.

Gel permeation chromatography (GPC). Molecular weight distribution of synthesised polymers was characterised by GPC with DMAc as the eluent. The Shimazu GPC modular system is composed of an auto-injector and a Phenomenex 5.0 μM bead size guard column (50 × 7.5 m) followed by four Phenomenex 5.0 μM bead size columns (10^5, 10^4, 10^3 and 10^2 Å), a differential refractive-index detector and a UV detector (λ = 305 nm). The DMAc GPC system was calibrated based on narrow molecular distribution of poly(methyl methacrylate) (PMMA) standards with molecular weights of 200 to 10^6 g mol^{-1}. In the model PET-RAFT polymerisation system, because the model monomer used in this work is poly-MA (PMA), we recalibrated the average molecular weights of PMA by introducing the corresponding Mark-Houwink-Kuhn-Sakurada (MHKS) coefficients and using the two-parameter

universal calibration strategy developed by T. Gruendling, T. Junkers, M. Guilhaus and C. Barner-Kowollik in 2010.[1]

Nuclear magnetic resonance (NMR) spectroscopy. NMR analysis was performed on a 300 MHz Bruker Advance III instrument installed with SampleXpress operating for ^{1}H with DMSO-d6 as solvent. Spectra were reported as chemical shift (δ) in ppm downfield from 0 ppm.

Matrix assisted laser desorption ionization-time of flight mass spectrometry (MALDI-TOF MS). MALDI-TOF MS analysis was performed at the Bioanalytical Mass Spectrometry Facility in University of New South Wales (BMSF, UNSW) on a Bruker ultrafleXtreme mass spectrometer in positive ion, linear mode using MeCN as the solvent.

Density functional theory (DFT) computation. DFT calculations and time-dependant (TD-)DFT calculations in this work were performed with Gaussian 09 D01 computational chemistry package installed in the Katana computational cluster located on campus at the University of New South Wales (UNSW), under an academic site license. Post-computational analysis of the wavefunction was conducted with Multiwfn software package[2] including the qualitative molecular surface analysis module. Isosurfaces including molecular orbitals and π-electron analysis and reduced density gradient analysis were visualised by the Multiwfn software package[2] or the Visual Molecular Dynamics (VMD) package;[3] post labelling was conducted with Illustrator CC 2020 under an educational subscription licence. For computational details of each part see respective sections below.

Supplementary Note 3. PET-RAFT polymerisation preparation

3.1 Selecting the model system

In this work, we selected a model system with DTPA as the RAFT (Supplementary Figure 1, top right) and MA as the monomer (Supplementary Figure 1 top left), not only because this is the most commonly used system for kinetic studies of PET-RAFT polymerisation, but also because it is an

excellent material for thermodynamic studies. Indeed, upon one MA monomer addition, DTPA becomes DTPA-MA (Supplementary Figure 1, bottom) but the secondary carbon attached to the trithiocarbonate group is highly similar in its chemical environment between DTPA and DTPA-MA (both with an alkyl group and a carboxylic group). Hence, the thermodynamic properties of DTPA before and after one or more MA additions should be basically the same, which is ideal for thermodynamic studies.

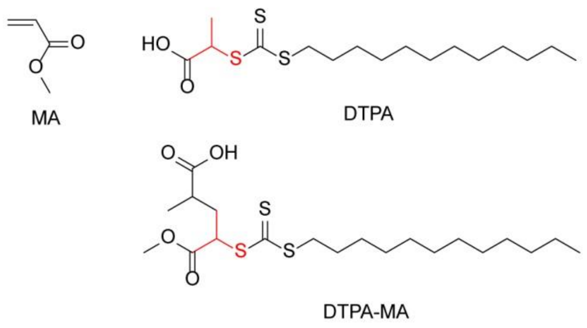

Supplementary Figure 1 Chemical structures of MA, DTPA and DTPA with one MA monomer addition (DTPA-MA).

3.2 Selecting irradiation conditions

10 mW/cm^2 was chosen as a relatively high light intensity for the configuration of our photo-reactions, in a consideration that the excited PC in the solution could be almost saturated at this light intensity, to keep the number of excited PCs roughly comparable for different PCs. At 10 mW/cm^2, 565 nm yellow light (photon flux 2.8×10^{16} s^{-1} cm^{-2}) for ZnOEP, 590 nm orange light (photon flux 3.0×10^{16} s^{-1} cm^{-2}) for ZnTPP, 625 nm red light (photon flux 3.2×10^{16} s^{-1} cm^{-2}) for TPP and 660 nm deep-red light (photon flux 3.3×10^{16} s^{-1} cm^{-2}) for ZnTSP have slightly different photon flux (maximum difference ~15%).

3.3 General procedures for dye-catalysed PET-RAFT polymerisation

The model PET-RAFT system contains DTPA as the CTA, MA as the monomer and DMSO as the solvent, at a fixed molar ratio of [CTA]:[monomer]:[PC] = 200:1:0.01 and a monomer to solvent ratio of 1:1 v/v for a oxidative quenching pathway (OQP). To enable a reductive quenching pathway (RQP)

or an oxygen-mediated RQP (O-RQP), triethylamine (TEA) was added as 0.5 equivalent to CTA. The exact recipes for preparing stock solutions (each has a total volume of 0.6 mL) for different dye-catalysed PET-RAFT polymerisations via any of the three quenching pathways were listed as Supplementary Table 1-14. The stock solution was transferred into a 0.9 mL FTNIR glass cuvette (1 cm × 2 mm × 5 cm) capped with a rubber septum and sealed with Parafilm and covered with aluminium foil before irradiation. Polymerisations via an OQP or an RQP were deoxygenated by purging with nitrogen for 10 min prior to light irradiation, whereas polymerisations via an O-RQP were left in the presence of air (non-deoxygenated) to include oxygen in the system. The glass cuvettes were gently shaken every time after FTNIR to assist oxygen dissolution from the head space. The glass cuvettes were then sent for irradiation under LEDs with nominal wavelengths of 565 nm, 590 nm, 625 nm, 660 nm, 730 nm and 780 nm depending on λ_{max} of the PC used in the PET-RAFT polymerisations, at a light intensity of 10 mW/cm^2 and constant temperature of 25 °C (RT). The cuvettes were transferred to a sample holder manually for FTNIR measurements after irradiation of every specified time interval (10 min or 20 min depending on the polymerisation rate) during the course of polymerisation. Monomer conversions were calculated by taking the ratio of integrations of the wavenumber area 6250-6150 cm^{-1} for all curves at different reaction times to that of time zero. Aliquots were taken occasionally during the reaction for GPC analysis to determine number average molecular weights (M_n), dispersities (M_w/M_n) and molecular weight distributions.

Supplementary Table 1 Recipe for preparing the 0.6 mL stock solution of ZnOEP-catalysed PET-RAFT polymerization via OQP.

Compound	Molar Ratio	Amount	Mass (mg)	Volume (mL)
MA	200	3.33 mmol	286.8	0.300
DTPA	1	0.0167 mmol	5.9	-
ZnOEP	0.01	16.7 µmol	0.0996	-
TEA	0	0	0	0
DMSO	-	-	-	0.300
Gas	Nitrogen			

*As the PC and TEA are both in very low amount when present in the solution, they can be prepared as stock solutions in DMSO. We recommend the PC to be in a 1 mg/mL DMSO solution.

Supplementary Table 2 Recipe for preparing the 0.6 mL stock solution of ZnOEP-catalysed PET-RAFT polymerization via RQP.

Compound	Molar Ratio	Amount	Mass (mg)	Volume (mL)
MA	200	3.33 mmol	286.8	0.300
DTPA	1	0.0167 mmol	5.9	-
ZnOEP	0.01	0.167 µmol	0.0996	-
TEA	0.5	8.39 µmol	0.843	0.00116
DMSO	-	-	-	0.299
Gas	Nitrogen			

*As the PC and TEA are both in very low amount when present in the solution, they can be prepared as stock solutions in DMSO. We recommend the PC to be in a 1 mg/mL DMSO solution.

Supplementary Table 3 Recipe for preparing the 0.6 mL stock solution of ZnOEP-catalysed PET-RAFT polymerization via O-RQP.

Compound	Molar Ratio	Amount	Mass (mg)	Volume (mL)
MA	200	3.33 mmol	286.8	0.300
DTPA	1	0.0167 mmol	5.9	-
ZnOEP	0.01	0.167 µmol	0.0996	-
TEA	0.5	8.39 µmol	0.843	0.00116
DMSO	-	-	-	0.299
Gas	Air			

*As the PC and TEA are both in very low amount when present in the solution, they can be prepared as stock solutions in DMSO. We recommend the PC to be in a 1 mg/mL DMSO solution.

Supplementary Table 4 Recipe for preparing the 0.6 mL stock solution of ZnTPP-catalysed PET-RAFT polymerization via OQP.

Compound	Molar Ratio	Amount	Mass (mg)	Volume (mL)
MA	200	3.33 mmol	286.8	0.300
DTPA	1	0.0167 mmol	5.9	-
ZnTPP	0.01	16.7 µmol	0.1130	-
TEA	0	0	0	0
DMSO	-	-	-	0.300
Gas	Nitrogen			

*As the PC and TEA are both in very low amount when present in the solution, they can be prepared as stock solutions in DMSO. We recommend the PC to be in a 1 mg/mL DMSO solution.

Supplementary Table 5 Recipe for preparing the 0.6 mL stock solution of ZnTPP-catalysed PET-RAFT polymerization via RQP.

Compound	Molar Ratio	Amount	Mass (mg)	Volume (mL)
MA	200	3.33 mmol	286.8	0.300
DTPA	1	0.0167 mmol	5.9	-
ZnTPP	0.01	0.167 µmol	0.1130	-
TEA	0.5	8.39 µmol	0.843	0.00116
DMSO	-	-	-	0.299
Gas	Nitrogen			

*As the PC and TEA are both in very low amount when present in the solution, they can be prepared as stock solutions in DMSO. We recommend the PC to be in a 1 mg/mL DMSO solution.

Supplementary Table 6 Recipe for preparing the 0.6 mL stock solution of ZnTPP-catalysed PET-RAFT polymerization via O-RQP.

Compound	Molar Ratio	Amount	Mass (mg)	Volume (mL)
MA	200	3.33 mmol	286.8	0.300
DTPA	1	0.0167 mmol	5.9	-
ZnTPP	0.01	0.167 μmol	0.1130	-
TEA	0.5	8.39 μmol	0.843	0.00116
DMSO	-	-	-	0.299
Gas	Air			

*As the PC and TEA are both in very low amount when present in the solution, they can be prepared as stock solutions in DMSO. We recommend the PC to be in a 1 mg/mL DMSO solution.

Supplementary Table 7 Recipe for preparing the 0.6 mL stock solution of TPP-catalysed PET-RAFT polymerization via RQP.

Compound	Molar Ratio	Amount	Mass (mg)	Volume (mL)
MA	200	3.33 mmol	286.8	0.300
DTPA	1	0.0167 mmol	5.9	-
TPP	0.01	0.167 μmol	0.1024	-
TEA	0.5	8.39 μmol	0.843	0.00116
DMSO	-	-	-	0.299
Gas	Nitrogen			

*As the PC and TEA are both in very low amount when present in the solution, they can be prepared as stock solutions in DMSO. We recommend the PC to be in a 1 mg/mL DMSO solution.

Supplementary Table 8 Recipe for preparing the 0.6 mL stock solution of TPP-catalysed PET-RAFT polymerization via O-RQP.

Compound	Molar Ratio	Amount	Mass (mg)	Volume (mL)
MA	200	3.33 mmol	286.8	0.300
DTPA	1	0.0167 mmol	5.9	-
TPP	0.01	0.167 μmol	0.1024	-
TEA	0.5	8.39 μmol	0.843	0.00116
DMSO	-	-	-	0.299
Gas	Air			

*As the PC and TEA are both in very low amount when present in the solution, they can be prepared as stock solutions in DMSO. We recommend the PC to be in a 1 mg/mL DMSO solution.

Supplementary Table 9 Recipe for preparing the 0.6 mL stock solution of ZnTSP-catalysed PET-RAFT polymerization via O-RQP.

Compound	Molar Ratio	Amount	Mass (mg)	Volume (mL)
MA	200	3.33 mmol	286.8	0.300
DTPA	1	0.0167 mmol	5.9	-
ZnTSP	0.01	0.167 μmol	0.1263	-
TEA	0.5	8.39 μmol	0.843	0.00116
DMSO	-	-	-	0.299
Gas	Air			

*As the PC and TEA are both in very low amount when present in the solution, they can be prepared as stock solutions in DMSO. We recommend the PC to be in a 1 mg/mL DMSO solution.

Supplementary Table 10 Recipe for preparing the 0.6 mL stock solution of ZnTtBAzP-catalysed PET-RAFT polymerization via O-RQP.

Compound	Molar Ratio	Amount	Mass (mg)	Volume (mL)
MA	200	3.33 mmol	286.8	0.300
DTPA	1	0.0167 mmol	5.9	-
ZnTtBAzP	0.01	0.167 μmol	0.1003	-
TEA	0.5	8.39 μmol	0.843	0.00116
DMSO	-	-	-	0.299
Gas	Air			

*As the PC and TEA are both in very low amount when present in the solution, they can be prepared as stock solutions in DMSO. We recommend the PC to be in a 1 mg/mL DMSO solution.

Supplementary Table 11 Recipe for preparing the 0.6 mL stock solution of ZnTtBPC-catalysed PET-RAFT polymerization via O-RQP.

Compound	Molar Ratio	Amount	Mass (mg)	Volume (mL)
MA	200	3.33 mmol	286.8	0.300
DTPA	1	0.0167 mmol	5.9	-
ZnTtBPC	0.01	0.167 μmol	0.1231	-
TEA	0.5	8.39 μmol	0.843	0.00116
DMSO	-	-	-	0.299
Gas	Air			

*As the PC and TEA are both in very low amount when present in the solution, they can be prepared as stock solutions in DMSO. We recommend the PC to be in a 1 mg/mL DMSO solution.

Supplementary Table 12 Recipe for preparing the 0.6 mL stock solution of ZnPC-catalysed PET-RAFT polymerization via O-RQP.

Compound	Molar Ratio	Amount	Mass (mg)	Volume (mL)
MA	200	3.33 mmol	286.8	0.300
DTPA	1	0.0167 mmol	5.9	-
ZnPC	0.01	0.167 μmol	0.0963	-
TEA	0.5	8.39 μmol	0.843	0.00116
DMSO	-	-	-	0.299
Gas	Air			

*As the PC and TEA are both in very low amount when present in the solution, they can be prepared as stock solutions in DMSO. We recommend the PC to be in a 1 mg/mL DMSO solution.

Supplementary Table 13 Recipe for preparing the 0.6 mL stock solution of ZnOBOPC-catalysed PET-RAFT polymerization via O-RQP.

Compound	Molar Ratio	Amount	Mass (mg)	Volume (mL)
MA	200	3.33 mmol	286.8	0.300
DTPA	1	0.0167 mmol	5.9	-
ZnOBOPC	0.01	0.167 μmol	0.1923	-

TEA	0.5	8.39 μmol	0.843	0.00116
DMSO	-	-	-	0.299
Gas	Air			

*As the PC and TEA are both in very low amount when present in the solution, they can be prepared as stock solutions in DMSO. We recommend the PC to be in a 1 mg/mL DMSO solution.

Supplementary Table 14 Recipe for preparing the 0.6 mL stock solution of ZnTtBNPC-catalysed PET-RAFT polymerization via O-RQP.

Compound	Molar Ratio	Amount	Mass (mg)	Volume (mL)
MA	200	3.33 mmol	286.8	0.300
DTPA	1	0.0167 mmol	5.9	-
ZnTtBNPC	0.01	0.167 μmol	0.1670	-
TEA	0.5	8.39 μmol	0.843	0.00116
DMSO	-	-	-	0.299
Gas	Air			

*As the PC and TEA are both in very low amount when present in the solution, they can be prepared as stock solutions in DMSO. We recommend the PC to be in a 1 mg/mL DMSO solution.

Supplementary Note 4. Characterisation of synthesised polymers

4.1 Molecular weight distribution characterisation

In each of the polymerisations, four aliquots in the course of polymerisation at specific time points were taken to perform GPC characterisation. As shown in Supplementary Figure 2-15, all polymerisations exhibit complete shifts of the molecular weight distribution towards higher molecular weights during polymerisation and result in symmetric and narrow profiles for molecular weight distributions.

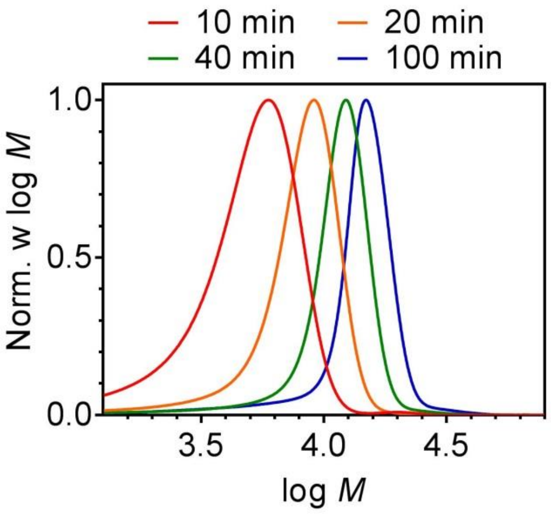

Supplementary Figure 2 | Normalised molecular weight distribution. Normalised molecular weight distribution of four GPC aliquots taken during ZnOEP-catalysed PET-RAFT polymerisation via OQP, denoted as the time point when each aliquot is taken according to the corresponding kinetic plot (main text, Figure 2i, green).

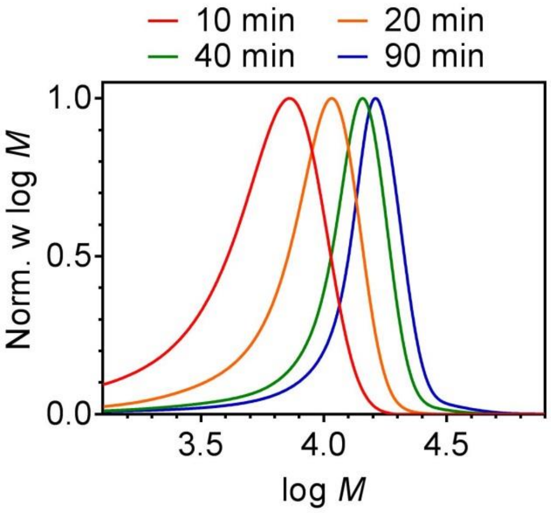

Supplementary Figure 3 | Normalised molecular weight distribution. Normalised molecular weight distribution of four GPC aliquots taken during ZnOEP-catalysed PET-RAFT polymerisation via RQP, denoted as the time point when each aliquot is taken according to the corresponding kinetic plot (main text, Figure 2i, blue).

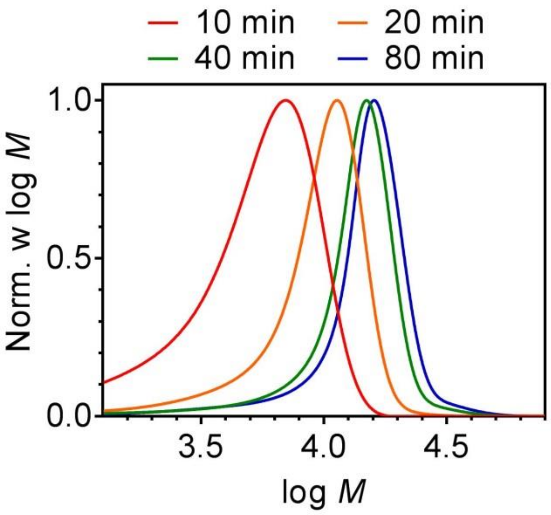

Supplementary Figure 4 | Normalised molecular weight distribution. Normalised molecular weight distribution of four GPC aliquots taken during ZnOEP-catalysed PET-RAFT polymerisation via O-RQP, denoted as the time point when each aliquot is taken according to the corresponding kinetic plot (main text, Figure 2i, red).

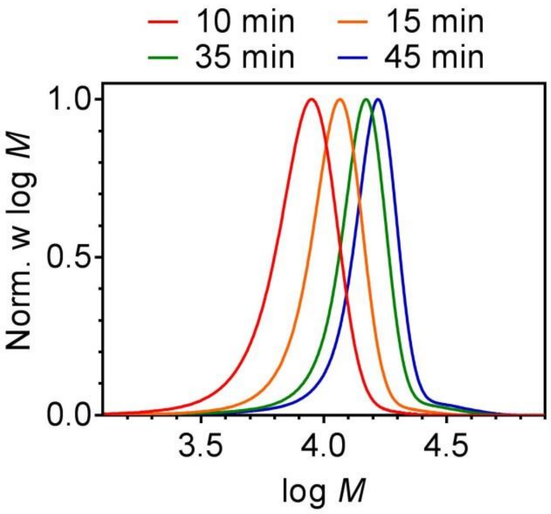

Supplementary Figure 5 | Normalised molecular weight distribution. Normalised molecular weight distribution of four GPC aliquots taken during ZnTPP-catalysed PET-RAFT polymerisation via OQP, denoted as the time point when each aliquot is taken according to the corresponding kinetic plot (main text, Figure 2j, green).

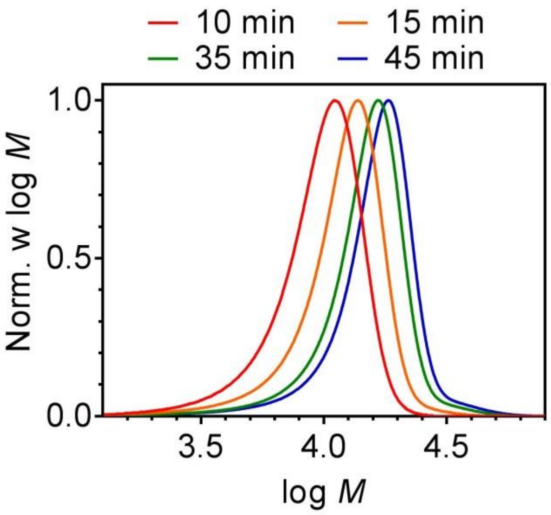

Supplementary Figure 6 | Normalised molecular weight distribution. Normalised molecular weight distribution of four GPC aliquots taken during ZnTPP-catalysed PET-RAFT polymerisation via RQP, denoted as the time point when each aliquot is taken according to the corresponding kinetic plot (main text, Figure 2j, blue).

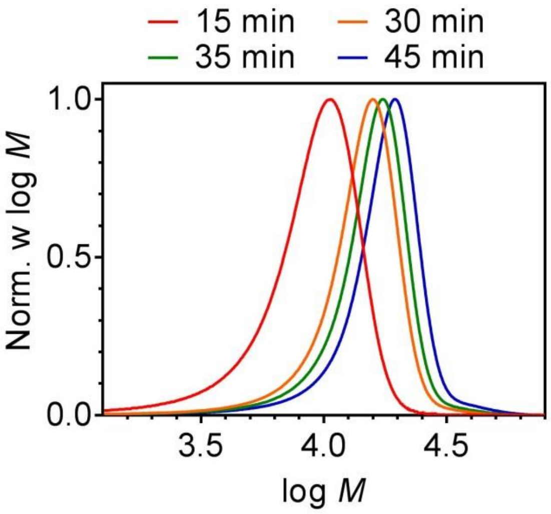

Supplementary Figure 7 | Normalised molecular weight distribution. Normalised molecular weight distribution of four GPC aliquots taken during ZnTPP-catalysed PET-RAFT polymerisation via O-RQP, denoted as the time point when each aliquot is taken according to the corresponding kinetic plot (main text, Figure 2j, red).

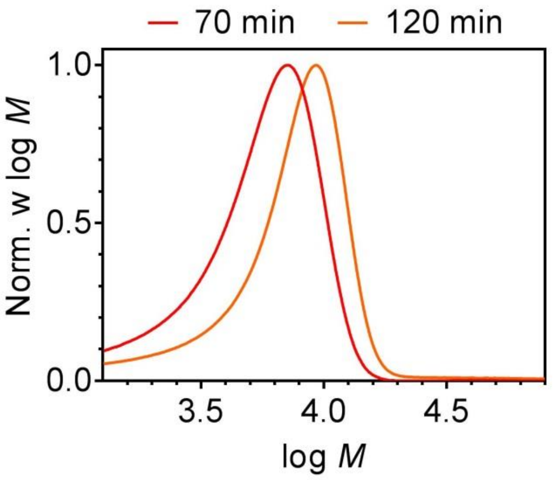

Supplementary Figure 8 | Normalised molecular weight distribution. Normalised molecular weight distribution of four GPC aliquots taken during TPP-catalysed PET-RAFT polymerisation via RQP, denoted as the time point when each aliquot is taken according to the corresponding kinetic plot (main text, Figure 2k, blue).

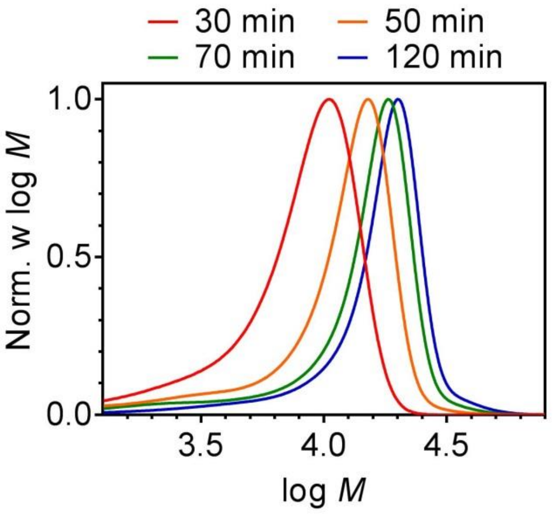

Supplementary Figure 9 | Normalised molecular weight distribution. Normalised molecular weight distribution of four GPC aliquots taken during TPP-catalysed PET-RAFT polymerisation via O-RQP, denoted as the time point when each aliquot is taken according to the corresponding kinetic plot (main text, Figure 2k, red).

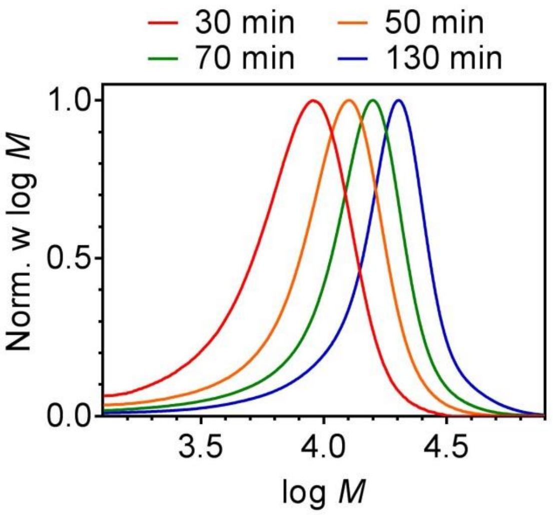

Supplementary Figure 10 | Normalised molecular weight distribution. Normalised molecular weight distribution of four GPC aliquots taken during ZnTSP-catalysed PET-RAFT polymerisation via O-RQP, denoted as the time point when each aliquot is taken according to the corresponding kinetic plot (main text, Figure 2l).

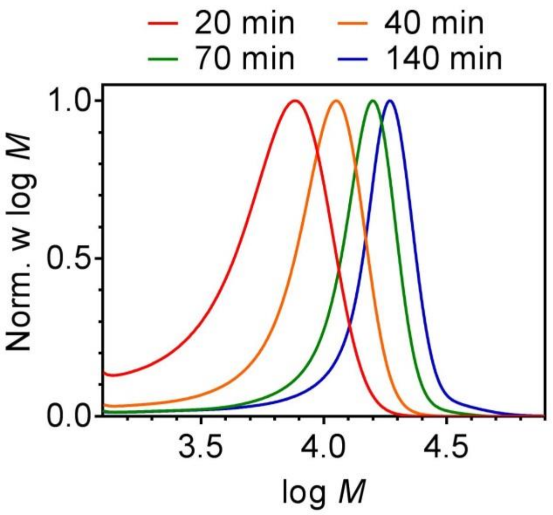

Supplementary Figure 11 | Normalised molecular weight distribution. Normalised molecular weight distribution of four GPC aliquots taken during ZnTtBAzP-catalysed PET-RAFT polymerisation via O-RQP, denoted as the time point when each aliquot is taken according to the corresponding kinetic plot (main text, Figure 5i).

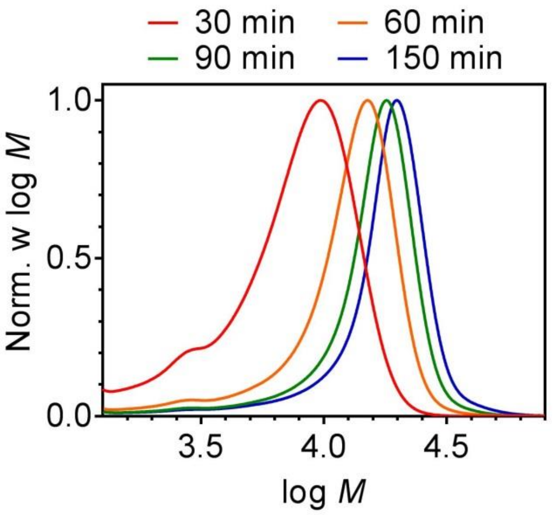

Supplementary Figure 12 | Normalised molecular weight distribution. Normalised molecular weight distribution of four GPC aliquots taken during ZnTtBPC-catalysed PET-RAFT polymerisation via O-RQP, denoted as the time point when each aliquot is taken according to the corresponding kinetic plot (main text, Figure 5j).

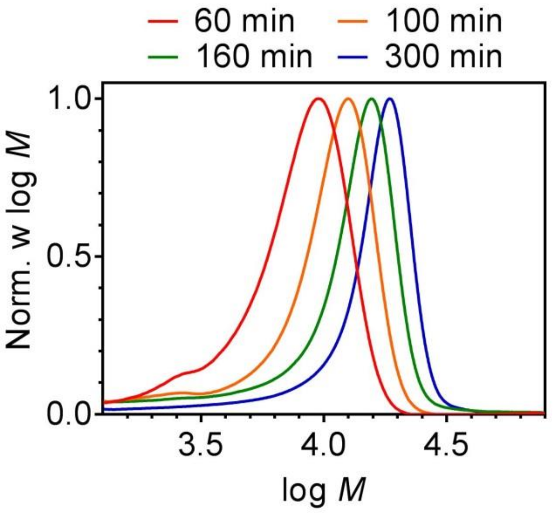

Supplementary Figure 13 | Normalised molecular weight distribution. Normalised molecular weight distribution of four GPC aliquots taken during ZnOBOPC-catalysed PET-RAFT polymerisation via O-RQP under 730 nm irradiation, denoted as the time point when each aliquot is taken according to the corresponding kinetic plot (main text, Figure 5k, orange).

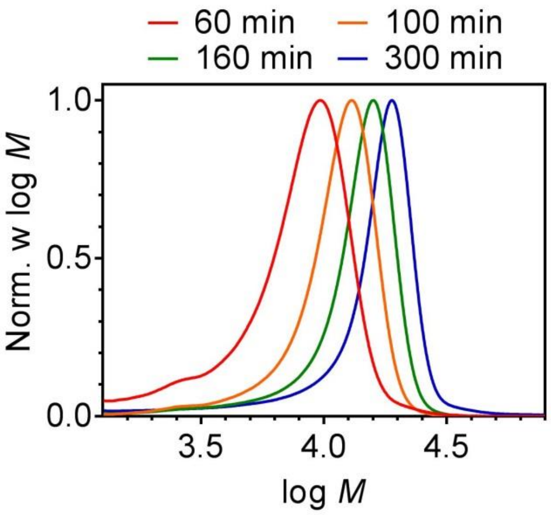

Supplementary Figure 14 | Normalised molecular weight distribution. Normalised molecular weight distribution of four GPC aliquots taken during ZnOBOPC-catalysed PET-RAFT polymerisation via O-RQP under 780 nm irradiation, denoted as the time point when each aliquot is taken according to the corresponding kinetic plot (main text, Figure 5k, red).

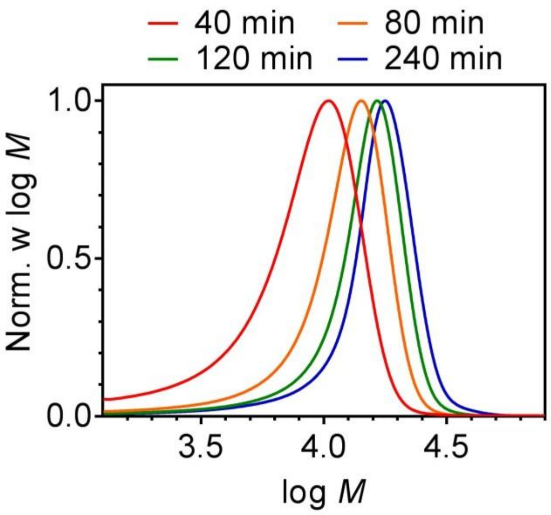

Supplementary Figure 15 | Normalised molecular weight distribution. Normalised molecular weight distribution of four GPC aliquots taken during ZnTtBNPC-catalysed PET-RAFT polymerisation via O-RQP, denoted as the time point when each aliquot is taken according to the corresponding kinetic plot (main text, Figure 5l).

4.2 Polymer purification

After polymerisation, 0.3 mL of the polymer solution was collected and added dropwise to 10 mL diethyl ether/petroleum spirit 7:3 (v/v) for precipitation. After centrifugation, the precipitates were collected and left dry before dissolving in minimal DCM. By repeating this precipitation-centrifugation-redissolution process for a couple of times, the precipitates can form a well-defined suspension. The obtained solid was placed under reduced pressure overnight until dryness to obtain purified polymers.

4.3 Analysis by NMR spectroscopy

To check the end-group fidelity of an O-RQP polymerisation and confirm that the presence of oxygen does not noticeably affect polymerization and end-group fidelity of polymers, we used the polymers obtained from ZnTSP-catalysed PET-RAFT polymerization via O-RQP as an example. The purified polymers were dissolved in 0.6 mL DMSO-d6 for 300 Hz ^{1}H NMR spectroscopy analysis **(Supplementary Figure 16)**. The well-correlated quantitative relationship between the 2 protons in the alkyl tail adjacent to the trithiocarbonate group (b, δ 3.82, 2 H, Integral: 2.14), the proton of the last added monomer on the carbon backbone adjacent to the trithiocarbonate group (a, δ 4.76, 1 H, Integral: 1.13), the three protons on the methyl group adjacent to the carboxylic group (d, δ 1.05, 3 H, Integral: 3.01) and the 3 protons attached to the ending carbon in the alkyl tail (c, δ 0.85, 3 H, Integral: 3.00) substantially confirmed the end group fidelity of the synthesised polymers. In addition, the NMR-determined average molecular weight $M_{n,NMR}$ = 12,300 g/mol correlates well with the theoretical value $M_{n,th}$ = 13,500 g/mol.

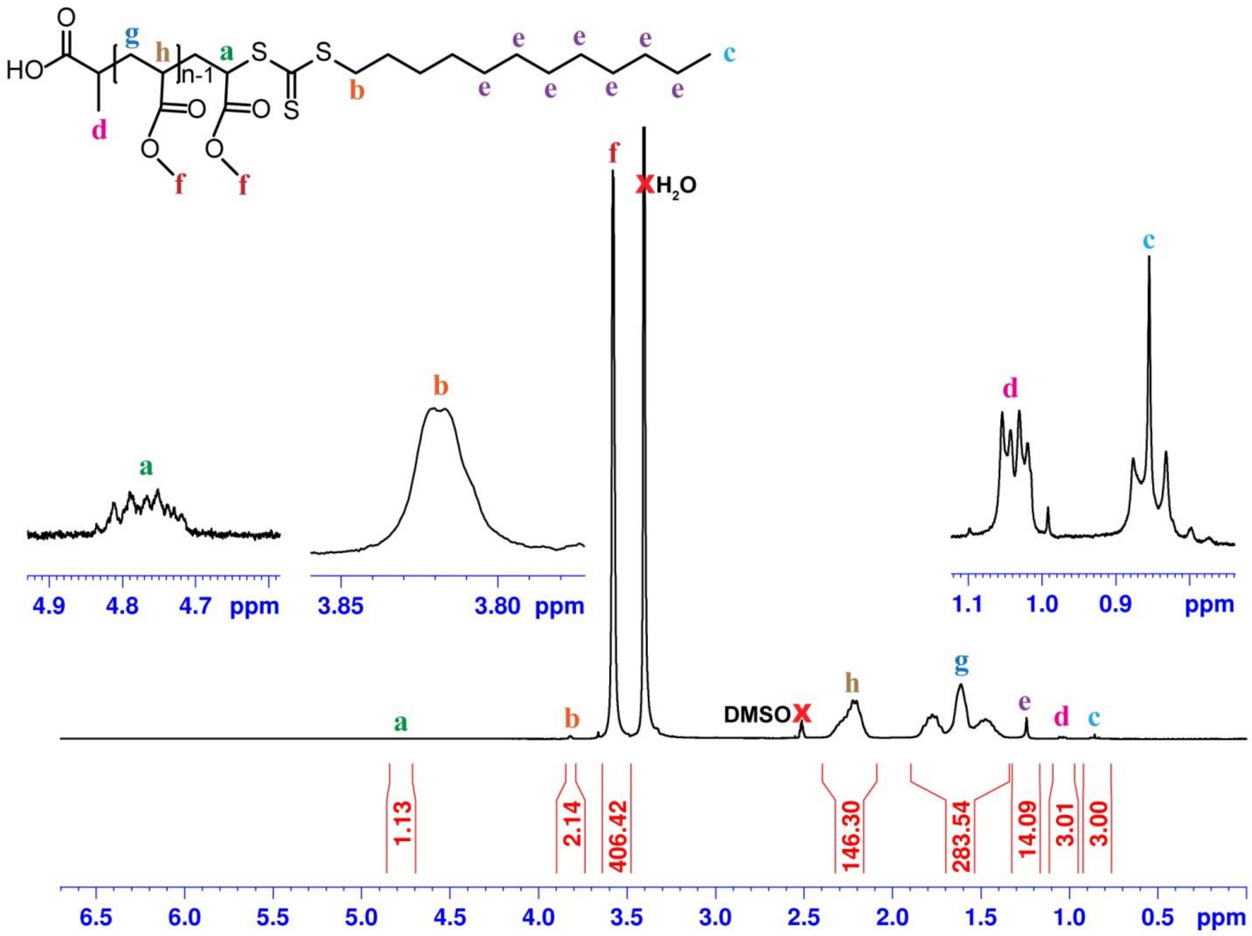

Supplementary Figure 16 | ^{1}H NMR spectrum of the synthesized PMA polymer. ^{1}H NMR spectrum (300 MHz, DMSO-d6) for purified PMA synthesised through ZnTSP-catalysed PET-RAFT polymerization via O-RQP at a molar ratio of [MA]:[DTPA]:[TEA]:[ZnTSP] = 200:1:0.5:0.01 in the presence of oxygen. The monomer conversion was driven to 76.4% before polymer purification.

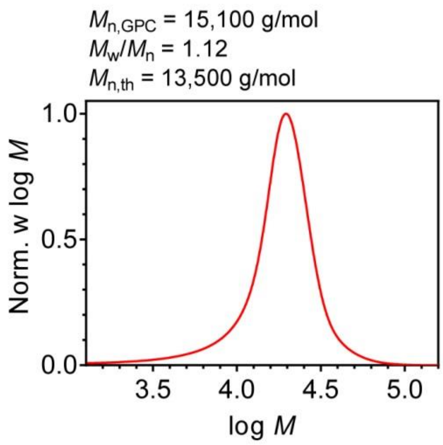

Supplementary Figure 17 | Normalised molecular weight distribution. Molecular weight distribution analysis determined by GPC of the same batch of the synthesised polymers characterised in Supplementary Figure 16. The purified PMA was synthesised through ZnTSP-catalysed PET-RAFT polymerization via O-RQP at a molar ratio of [MA]:[DTPA]:[TEA]:[ZnTSP] = 200:1:0.5:0.01 in the presence of oxygen. The monomer conversion was driven to 76.4% before polymer purification. The GPC-determined average molecular weight $M_{n,GPC}$ = 15,100 g/mol is close to $M_{n,NMR}$ = 12,300 g/mol and $M_{n,th}$ = 13,500 g/mol.

4.4 MALDI-TOF MS analysis

Although NMR spectroscopy has already confirmed the high end-group fidelity of the synthesised polymers from ZnTSP-catalysed PET-RAFT polymerization of MA via O-RQP, MALDI-TOF MS can provide another perspective from molecular weights of isolated polymers for the reader's reference. Here, to provide an extra experimental proof for that the presence of oxygen does not noticeably affect polymerization and end-group fidelity of polymers, we further performed MALDI-TOF MS for a purified poly-DMA (PDMA) sample synthesised through ZnTSP-catalysed PET-RAFT polymerization via O-RQP at a molar ratio of [DMA]:[DTPA]:[TEA]:[ZnTSP] = 100:1:0.5:0.01 in the presence of oxygen. The monomer DMA is used here because the resultant PDMA polymers has better dispersibility, and is easier to be ionised and clearer to be identified in the MALDI-TOF characterisation. The monomer conversion was driven to 22.5% (degree of polymerization DP ~23) to match the suitable molecular weight range for MALDI-TOF MS analysis on PDMA. GPC analysis of the used sample revealed a $M_{n,GPC}$ of 3000 g/mol and a M_w/M_n of 1.26. As shown in **Supplementary Figure 18**, the major peaks are discretely distributed with a spacing of 99.13 g/mol which corresponds to the molecular weight of the monomer DMA. As an example, by zooming into the molecular weight range of 2300-2500 g/mol, we observed that major peaks (inset) excellently matches theoretical predication of PDMA with DTPA chain-end group plus Na+ (chemical structure indicated above the full spectrum), within experimental error (< 2 g/mol). For the left peak in the

inset, the number of monomer insertion (n) is determined to be 20, whereas the right peak in the inset is assigned to n = 21.

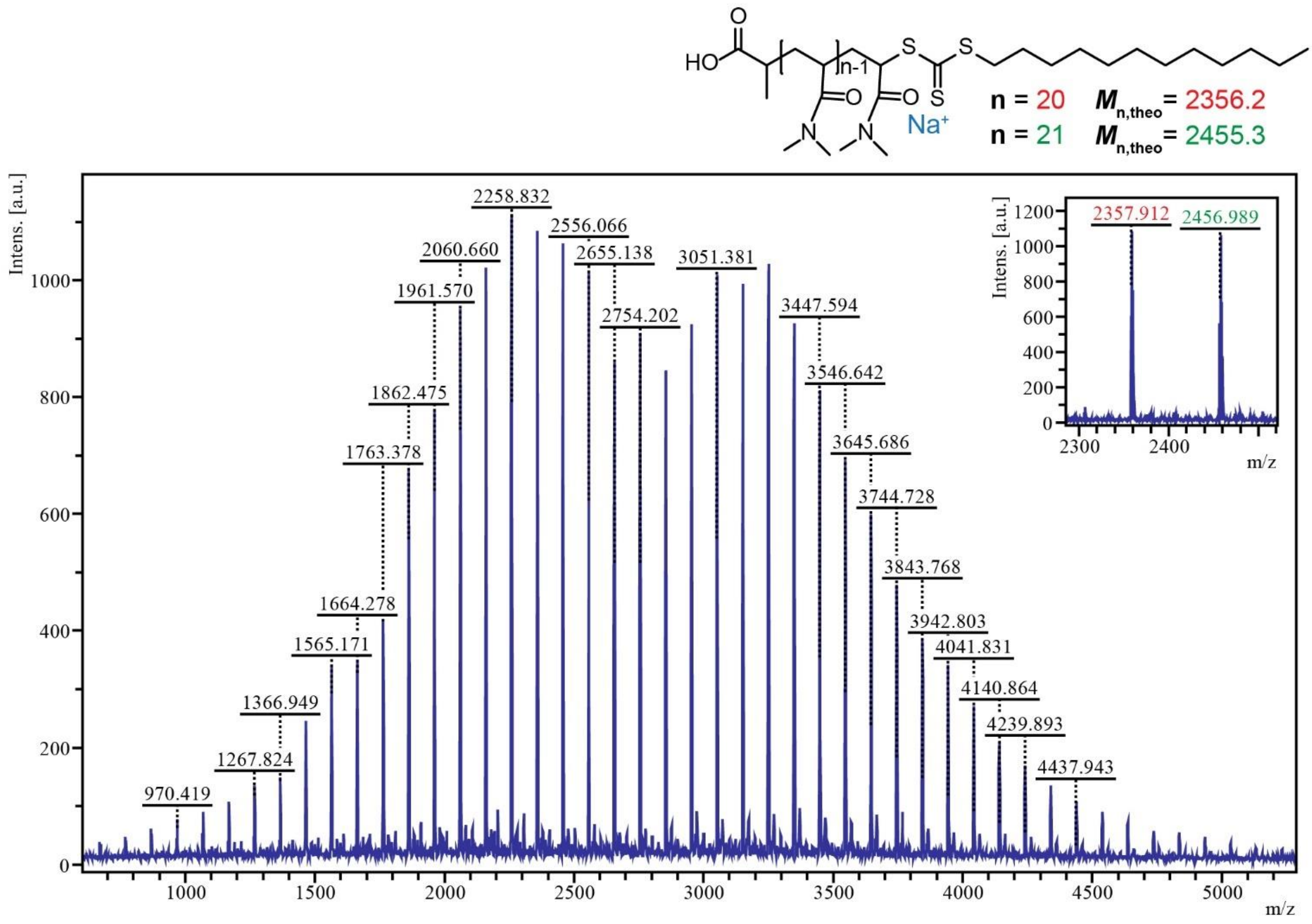

Supplementary Figure 18 | MALDI-TOF MS of the purified polymer. MALDI-TOF MS of a purified PDMA sample synthesised through ZnTSP-catalysed PET-RAFT polymerization via O-RQP.

4.5 End-group fidelity confirmation by chain-extension experiments

After purification of the synthesised polymers, we performed chain extension experiments to assess the fidelity/accessibility of the RAFT end-groups using each batch of purified PMA for PET-RAFT polymerisation catalysed by the same PC, via the same quenching pathway and under the same irradiation with which the polymers were made. The chain extension polymersations were performed at [DMA]:[PDMA-macroRAFT] = 600:1 and driven to around 20-30% monomer conversions. As

shown in Supplementary Figure 19, complete shifts of the molecular weight distribution to higher molecular weights confirmed the high retention of trithiocarbonate end groups in all cases (Figures S19-32).

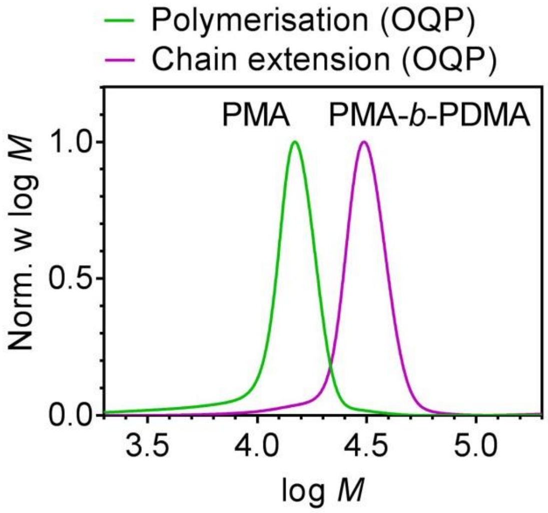

Supplementary Figure 19 | Normalised molecular weight distribution. Molecular weight distributions for the synthesised PMA after ZnOEP-catalysed PET-RAFT polymerisation via OQP (green line) and the chain-extended PMA-*b*-PDMA after chain extension ZnOEP-catalysed PET-RAFT polymerization via OQP (purple line). Complete shift of molecular weight distributions to higher molecular weights confirmed the high retention of the trithiocarbonate chain end group. The final PMA-*b*-PDMA was characterised by GPC and exhibited M_n = 29,000 g/mol and M_w/M_n = 1.08.

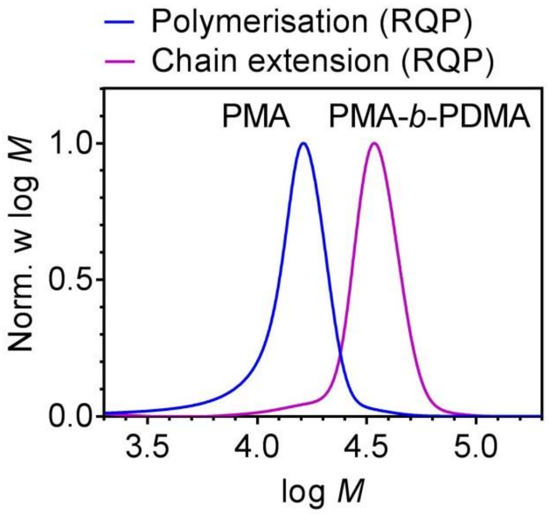

Supplementary Figure 20 | Normalised molecular weight distribution. Molecular weight distributions for the synthesised PMA after ZnOEP-catalysed PET-RAFT polymerisation via RQP (blue line) and the chain-extended PMA-*b*-PDMA after chain extension ZnOEP-catalysed PET-RAFT polymerization via RQP (purple line). Complete shift of molecular weight distributions to higher molecular weights confirmed the high retention of the trithiocarbonate chain end group. The final PMA-*b*-PDMA was characterised by GPC and exhibited M_n = 31,600 g/mol and M_w/M_n = 1.10.

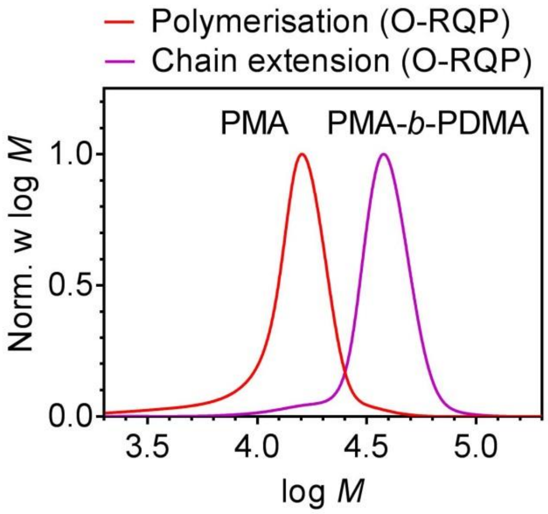

Supplementary Figure 21 | Normalised molecular weight distribution. Molecular weight distributions for the synthesised PMA after ZnOEP-catalysed PET-RAFT polymerisation via O-RQP (red line) and the chain-extended PMA-*b*-PDMA after chain extension ZnOEP-catalysed PET-RAFT polymerization via O-RQP (purple line). Complete shift of molecular weight distributions to higher molecular weights confirmed the high retention of the trithiocarbonate chain end group. The final PMA-*b*-PDMA was characterised by GPC and exhibited M_n = 34,600 g/mol and M_w/M_n = 1.11.

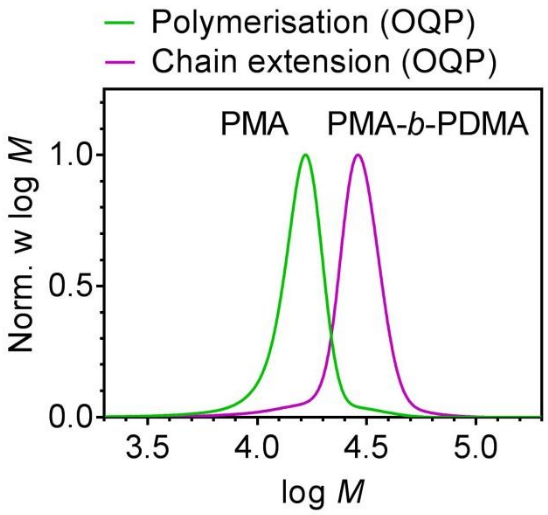

Supplementary Figure 22 | Normalised molecular weight distribution. Molecular weight distributions for the synthesised PMA after ZnTPP-catalysed PET-RAFT polymerisation via OQP (green line) and the chain-extended PMA-*b*-PDMA after chain extension ZnTPP-catalysed PET-RAFT polymerization via OQP (purple line). Complete shift of molecular weight distributions to higher molecular weights confirmed the high retention of the trithiocarbonate chain end group. The final PMA-*b*-PDMA was characterised by GPC and exhibited M_n = 27,200 g/mol and M_w/M_n = 1.09.

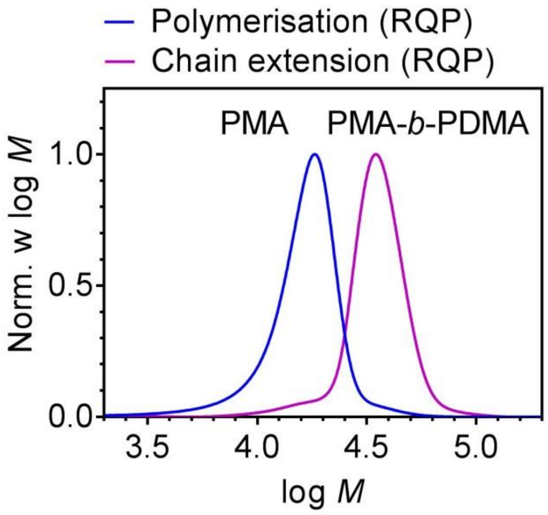

Supplementary Figure 23 | Normalised molecular weight distribution. Molecular weight distributions for the synthesised PMA after ZnTPP-catalysed PET-RAFT polymerisation via RQP (blue line) and the chain-extended PMA-*b*-PDMA after chain extension ZnTPP-catalysed PET-RAFT polymerization via RQP (purple line). Complete shift of molecular weight distributions to higher

molecular weights confirmed the high retention of the trithiocarbonate chain end group. The final PMA-*b*-PDMA was characterised by GPC and exhibited M_n = 32,200 g/mol and M_w/M_n = 1.11.

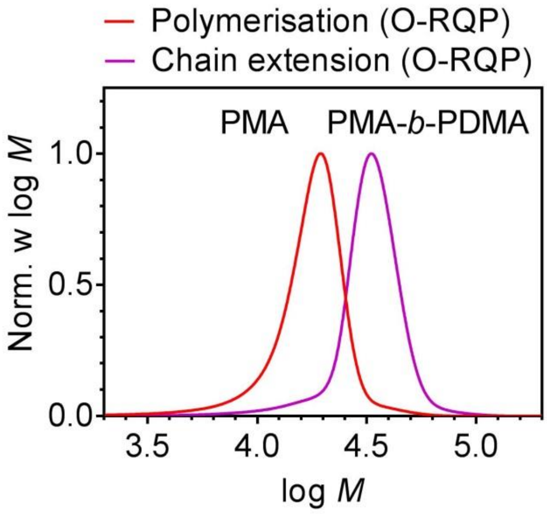

Supplementary Figure 24 | Normalised molecular weight distribution. Molecular weight distributions for the synthesised PMA after ZnTPP-catalysed PET-RAFT polymerisation via O-RQP (red line) and the chain-extended PMA-*b*-PDMA after chain extension ZnTPP-catalysed PET-RAFT polymerization via O-RQP (purple line). Complete shift of molecular weight distributions to higher molecular weights confirmed the high retention of the trithiocarbonate chain end group. The final PMA-*b*-PDMA was characterised by GPC and exhibited M_n = 30,400 g/mol and M_w/M_n = 1.12.

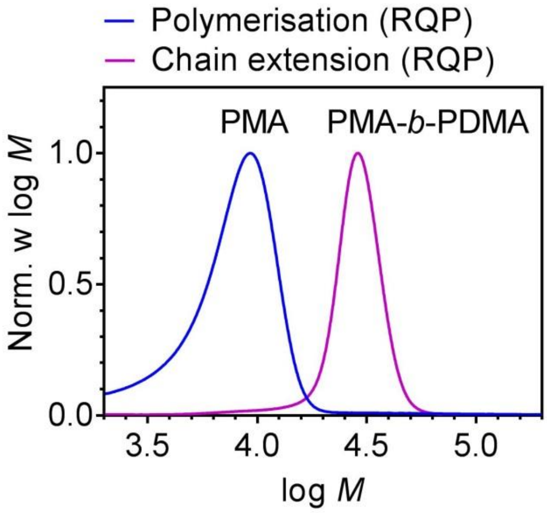

Supplementary Figure 25 | Normalised molecular weight distribution. Molecular weight distributions for the synthesised PMA after TPP-catalysed PET-RAFT polymerisation via RQP (blue

line) and the chain-extended PMA-*b*-PDMA after chain extension TPP-catalysed PET-RAFT polymerization via RQP (purple line). Complete shift of molecular weight distributions to higher molecular weights confirmed the high retention of the trithiocarbonate chain end group. The final PMA-*b*-PDMA was characterised by GPC and exhibited M_n = 26,700 g/mol and M_w/M_n = 1.09.

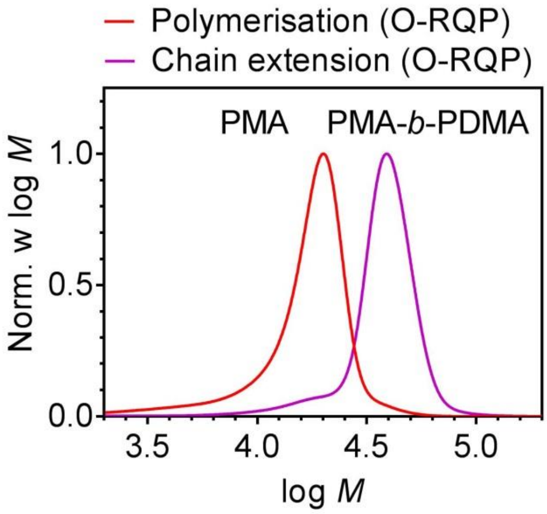

Supplementary Figure 26 | Normalised molecular weight distribution. Molecular weight distributions for the synthesised PMA after TPP-catalysed PET-RAFT polymerisation via O-RQP (red line) and the chain-extended PMA-*b*-PDMA after chain extension TPP-catalysed PET-RAFT polymerization via O-RQP (purple line). Complete shift of molecular weight distributions to higher molecular weights confirmed the high retention of the trithiocarbonate chain end group. The final PMA-*b*-PDMA was characterised by GPC and exhibited M_n = 35,200 g/mol and M_w/M_n = 1.11.

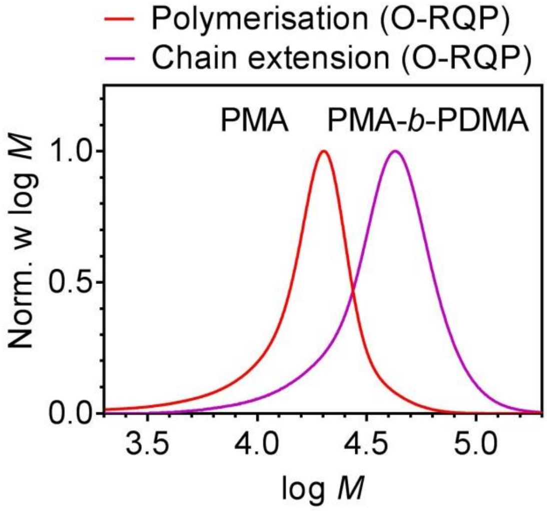

Supplementary Figure 27 | Normalised molecular weight distribution. Molecular weight distributions for the synthesized PMA after ZnTSP-catalysed PET-RAFT polymerisation via O-RQP (red line) and the chain-extended PMA-*b*-PDMA after chain extension ZnTSP-catalysed PET-RAFT polymerization via O-RQP (purple line). Complete shift of molecular weight distributions to higher molecular weights confirmed the high retention of the trithiocarbonate chain end group. The final PMA-*b*-PDMA was characterised by GPC and exhibited M_n = 36,800 g/mol and M_w/M_n = 1.18.

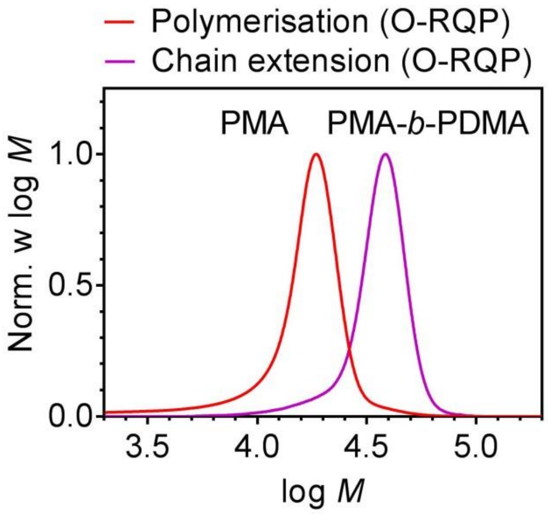

Supplementary Figure 28 | Normalised molecular weight distribution. Molecular weight distributions for the synthesised PMA after ZnTtBAzP-catalysed PET-RAFT polymerisation via O-RQP (red line) and the chain-extended PMA-*b*-PDMA after chain extension ZnTtBAzP-catalysed PET-RAFT polymerization via O-RQP (purple line). Complete shift of molecular weight distributions to higher molecular weights confirmed the high retention of the trithiocarbonate chain end group. The final PMA-*b*-PDMA was characterised by GPC and exhibited M_n = 35,800 g/mol and M_w/M_n = 1.05.

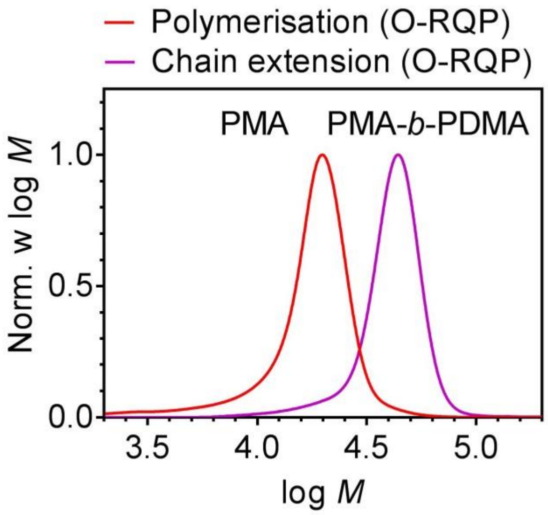

Supplementary Figure 29 | Normalised molecular weight distribution. Molecular weight distributions for the synthesised PMA after ZnTtBPC-catalysed PET-RAFT polymerisation via O-RQP (red line) and the chain-extended PMA-*b*-PDMA after chain extension ZnTtBPC-catalysed PET-RAFT polymerization via O-RQP (purple line). Complete shift of molecular weight distributions to higher molecular weights confirmed the high retention of the trithiocarbonate chain end group. The final PMA-*b*-PDMA was characterised by GPC and exhibited M_n = 40,900 g/mol and M_w/M_n = 1.06.

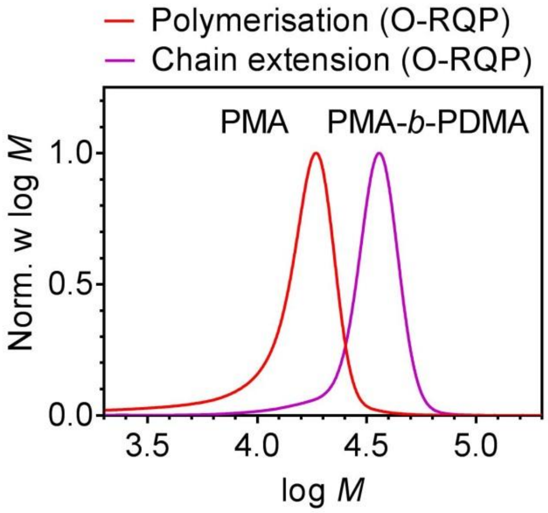

Supplementary Figure 30 | Normalised molecular weight distribution. Molecular weight distributions for the synthesised PMA after ZnOBOPC-catalysed PET-RAFT polymerisation via O-RQP (red line) under 730 nm irradiation and the chain-extended PMA-*b*-PDMA after chain extension

ZnOBOPC-catalysed PET-RAFT polymerization via O-RQP (purple line) under 730 nm irradiation. Complete shift of molecular weight distributions to higher molecular weights confirmed the high retention of the trithiocarbonate chain end group. The final PMA-*b*-PDMA was characterised by GPC and exhibited M_n = 33,400 g/mol and M_w/M_n = 1.06.

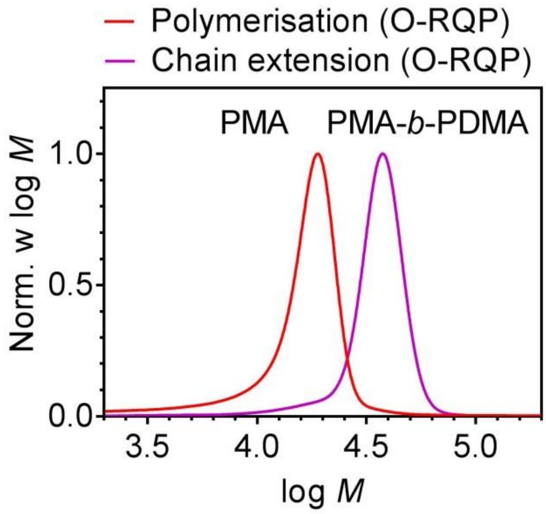

Supplementary Figure 31 | Normalised molecular weight distribution. Molecular weight distributions for the synthesised PMA after ZnOBOPC-catalysed PET-RAFT polymerisation via O-RQP (red line) under 780 nm irradiation and the chain-extended PMA-*b*-PDMA after chain extension ZnOBOPC-catalysed PET-RAFT polymerization via O-RQP (purple line) under 780 nm irradiation. Complete shift of molecular weight distributions to higher molecular weights confirmed the high retention of the trithiocarbonate chain end group. The final PMA-*b*-PDMA was characterised by GPC and exhibited M_n = 35,000 g/mol and M_w/M_n = 1.06.

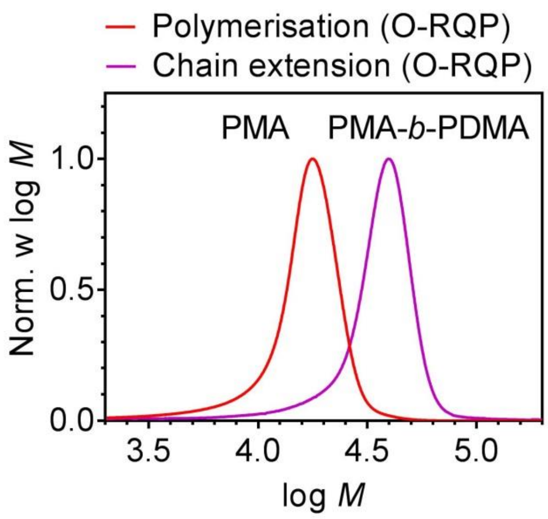

Supplementary Figure 32 | Normalised molecular weight distribution. Molecular weight distributions for the synthesised PMA after ZnTtBNPC-catalysed PET-RAFT polymerisation via O-RQP (red line) under 780 nm irradiation and the chain-extended PMA-*b*-PDMA after chain extension ZnTtBNPC-catalysed PET-RAFT polymerization via O-RQP (purple line). Complete shift of molecular weight distributions to higher molecular weights confirmed the high retention of the trithiocarbonate chain end group. The final PMA-*b*-PDMA was characterised by GPC and exhibited M_n = 35,900 g/mol and M_w/M_n = 1.07.

Supplementary Note 5. Evaluation of photophysical structure-property relationships

5.1 Photophysical processes

PC at the ground state (GS) is excited by absorbing a photon from a light beam, to reach an excited state (ES). The ability of the PC absorbing a photon at λ_{max} is the maximum absorbance σ_{max} corresponding to the oscillator strength of the vertical excitation to the lowest singlet excited state (S_1). Subsequently, a series of non-adiabatic crossing dynamics may occur to derive an excited state with a lifetime sufficiently long for a specific photochemical reaction. As shown in the Jablonski diagram (Supplementary Figure 33), upon photoexcitation at λ_{max}, the PC reaches the lowest singlet excited state (S_1), which can revert to the singlet ground state (S_0) by fluorescence or internal

conversion (IC), or toggle to the lowest triplet excited state (T_1) by intersystem crossing (ISC). As presented in Supplementary Figure 33, Φ_T describes the total quantum yield of T_1 resulting from S_1 evolutions, including both direct $S_1 \rightarrow T_1$ ISC and also $S_1 \rightarrow T_2$ (or higher) ISC followed by IC to T_1.[4] Because of the spin-forbidden nature of ISC, normally, T_1 exhibits lifetime up to the ms or sub-ms level, which is usually sufficient for diffusive encounter of reactants in dilute solutions and is thus considered to be dominant active species for photochemical electron transfer transformations.[5-8] Therefore, σ_{max} and Φ_T should have positive contribution to the efficiency or rate of the PET process (in a roughly proportional manner for dilute solutions), which determines how much T_1 is available to the redox reaction.

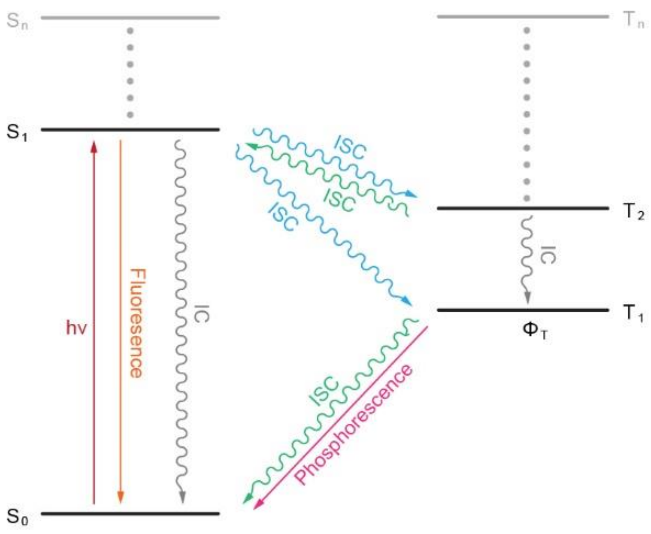

Supplementary Figure 33 | Jablonski diagram of a typical PC. ISC: intersystem crossing; IC: internal conversion. The Jablonski diagram is presented according to the actual excited state energies (calculated) of ZnOEP as an example.

5.2 Structure-property relationships for porphyrin dyes

The excited PC is both a better oxidant and a better reductant compared to its GS, in that the excited energy can be utilised to facilitate the redox activities.[5,9] Hence, λ_{max} corresponding to S_1 vertical excitation can be (but not necessarily) a rough indicator for the T_1 excitation energy and thereby have a notable contribution to the T_1 redox capabilities. Although not accurate, this is the simplest

way to estimate the excited state energies given a known chromophore before any further experimental or computational investigations. We conducted ultraviolet-visible (UV-Vis) spectrophotometry (Supplementary Figure 34 a-d) and observed increased λ_{max} from ZnOEP (575 nm), ZnTPP (598 nm), TPP (645 nm) to ZnTSP (660 nm). To interpret the structure-property relationships within this aspect, we performed π-conjugation analyses (Supplementary Figure 34 e-h) and frontier molecular orbital analyses (Supplementary Figure 34 i-l).

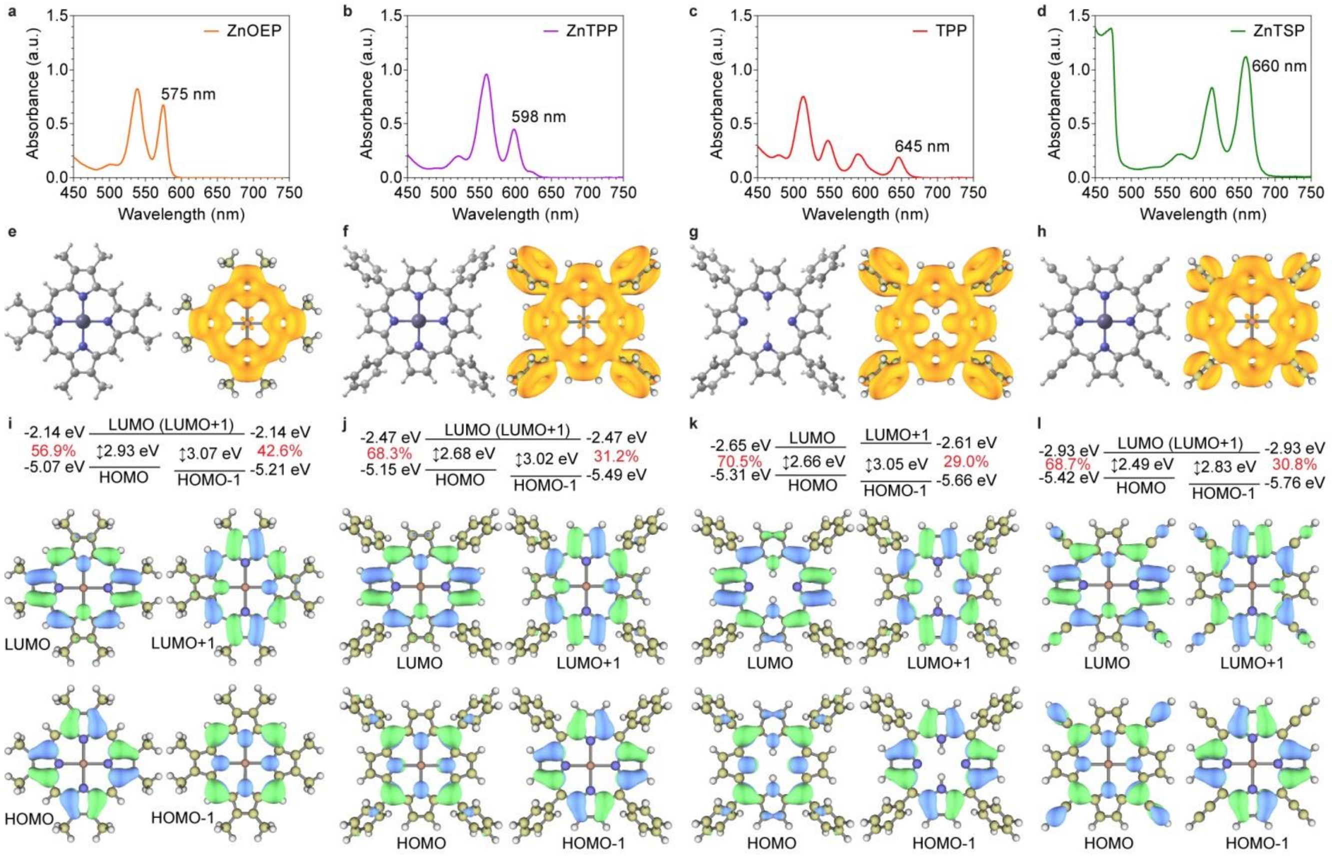

Supplementary Figure 34 | Photophysical studies of PCs. a-d, UV-Vis spectra of ZnOEP (**a**), ZnTPP (**b**), TPP (**c**) and ZnTSP (**d**) with λ_{max} denoted, measured with solvent DMSO to MA ration of 1:1 v/v and 50 ppm PC with respect to MA. **e-h**, Molecular geometries (left) and visualised π-electron localised orbital localiser (LOL) analyses (right) of ZnOEP (**e**), ZnTPP (**f**), TPP (**g**) and ZnTSP (**h**) respectively (isovalue = 0.2). **i-l**, Energy level diagrams (top) and visualisation (bottom) of HOMO-1, HOMO, LUMO and LUMO+1 (isovalue = 0.3) of ZnOEP (**i**), ZnTPP (**j**), TPP (**k**) and ZnTSP (**l**) respectively; percentage contribution of the respective electron transition between molecular orbitals to S_1 is denoted in red.

For planar macrocyclic dyes with the same porphyrin core and moderate substituents, extended conjugation is the main cause of bathochromic effect (i.e. longer λ_{max}). To gain an insight into this aspect, we performed density functional theory (DFT) calculations by the Gaussian09 D01 package at B3LYP-GD3BJ/6-311G* level of theory (pseudopotential basis set LanL2TZ for zinc; SMD-DMSO solvation model). After deriving wave function information of the ground state after geometry optimisation, localised-orbital locator (LOL) analyses were conducted by the Multiwfn package[10] to visualise the π-electron delocalisation within the macrocycles (yellow surfaces in Supplementary Figure 34e-h, right, isovalue = 0.2). In these calculations, as a common practice, ethyl groups in ZnOEP was simplified into methyl groups and triisopropylsilylethynyl groups in ZnTSP was simplified into ethynyl groups to reduce computation expense, since they exert minimal effect on photophysical and electrochemical properties of the PC. Simply speaking, ZnTPP (Supplementary Figure 34f, λ_{max} = 598 nm) bears extended π-conjugation involving four tilted phenyl groups compared to ZnOEP (Supplementary Figure 34e, λ_{max} = 575 nm), which leads to 23 nm red-shift. TPP (Supplementary Figure 34g, λ_{max} = 645 nm) with non-bonded lone pair electrons on the two pyrrole nitrogen atoms contributes to additional extended conjugation compared to ZnTPP, which results in 47 nm red-shift. Lastly, the four acetylenic groups in ZnTSP (Supplementary Figure 34h, λ_{max} = 660 nm) appears to yield enhanced conjugation and hence a greater bathochromic effect by 47 nm than phenyl groups (ZnTPP).

π-conjugation analysis is useful in rough and preliminary prediction of bathochromic effect given only the structure of the PC. However, the most accurate evaluation is to uncover the nature of relevant molecular orbitals and their contributions to S_1 excitation (corresponding to λ_{max} in most cases). The orbitals concerned here are the highest occupied molecular orbital (HOMO), the lowest unoccupied molecular orbital (LUMO), the second HOMO (HOMO-1) and the second LUMO (LUMO+1). HOMO-1, HOMO, LUMO and LUMO+1 were visualised by Multiwfn for all dyes at the ground state (Supplementary Figure 34i-l, bottom). Time-dependent DFT (TD-DFT) calculations by

Gaussian (considering 10 singlet ESs) revealed that HOMO→LUMO transitions have a dominant ~60 % contribution to S_1 vertical excitation for ZnOEP and around 70% for ZnTPP, TPP and ZnTSP, while the rest 30-40% is mainly contributed by HOMO-1→LUMO+1 transitions (Supplementary Figure 34i-l, top, denoted as red in percentage). With regard to ZnTPP, although the moderate electron-withdrawing effect of its phenyl groups moves all of HOMO-1, HOMO, LUMO and LUMO+1 energy levels down compared to ZnOEP, a small content of phenyl-π (where part of the HOMO is populated) to porphyrin-π* (where LUMO is localised, Supplementary Figure 34j) charge transfer (CT) character cancelled out the lowering of HOMO energy level to some extent, thus narrowing the HOMO-LUMO energy gap and leading to the 23 nm bathochromic effect. Interestingly, this cancelling effect seems to have also caused a reverse HOMO/HOMO-1 ordering of ZnTPP (Supplementary Figure 34j) as compared to ZnOEP (Supplementary Figure 34i). When it comes to TPP, despite the absence of the electron-donating zinc leading to lowered energy levels for all molecular orbitals, it is mostly similar to ZnTPP at the first glimpse (Supplementary Figure 34j-k). However, there is a critical difference between the two dyes in the arrangement of pyrrole-nitrogen valence electrons. In ZnTPP, the four lone-pair (n) electrons were equally shared by four pyrrole-nitrogen atoms due to presence of the zinc centre; whereas in TPP (Supplementary Figure 34k), the four n electrons disproportionately localised on two opposite nitrogen atoms. Thereby, the merged LUMO/LUMO+1 energy level (as of ZnTPP) splits into a lower-lying LUMO of TPP without any n* contribution and a higher-lying LUMO+1 of TPP with notable n* contribution, which consequently results in the much narrowed HOMO-LUMO energy gap of TPP and a 47 nm bathochromic effect compared to ZnTPP. The main difference between ZnTSP (Supplementary Figure 34l) and ZnTPP is the acetylenic groups (instead of phenyl groups), which largely stabilises LUMO/LUMO+1 by delocalising their populations to the low-lying π* of acetylenic groups. This has caused significant narrowing in the HOMO-LUMO energy gap of ZnTSP and led to 62 nm bathochromic effect compared to ZnTPP.

Indeed, ZnOEP, ZnTPP, TPP and ZnTSP (sharing the same porphyrin core) in sequence exhibit increased λ_{max} from shorter to longer (Supplementary Figure 34a-d), coinciding with increased selectivity in activating certain quenching pathways of PET-RAFT (main text, Figure 2a-d).

On the other hand, the absorbance at λ_{max} (σ_{max}) and the triplet quantum yield (Φ_T) determine how efficient a PC converts light energy to chemical energies that can be used for SET reactions in dilute solutions. Hence, σ_{max} and Φ_T should positively contribute to efficiencies of subsequent photochemical reactions roughly in a proportional manner and should be considered before evaluating the thermodynamic favourability or selectivity of a PC in activating different quenching pathways. For example, alkylated porphyrins typically exhibit Φ_T of around 0.4-0.5 (e.g. tetramethyl porphyrin has a Φ_T of 0.43) and tetraphenyl porphyrins typically has Φ_T of around 0.8-0.9 (e.g. ZnTPP has Φ_T = 0.88 and TPP has Φ_T = 0.82).

In particular, as T_1 redox properties of ZnOEP and ZnTPP are both thermodynamically favourable for efficient OQP PET-RAFT and both dyes exhibit similar absorbance (Supplementary Figure 34a-b), in terms of kinetics, ZnTPP still displayed a k_p^{app} almost doubled that of ZnOEP in OQP. Hence, we naturally attribute this to the almost doubled Φ_T of tetraphenyl porphyrins (0.8-0.9) compared with alkylated porphyrins (0.4-0.5). Besides, the ethyl substituents could also exhibit hinderance for molecular collision in the PET and SET processes, which could potentially retard the photocatalysis of ZnOEP. However, as the exact kinetics is not a focus of the present study and it can be affected by much more factors, herein we only consider thermodynamic perspective of these PCs.

5.3 Structure-property relationships for tetraaza-porphyrin and phthalocyanine dyes

We performed UV-vis spectroscopy for these four dyes and revealed λ_{max} = 582 nm for ZnTtBAzP, λ_{max} = 661 nm for ZnTtBPC, λ_{max} = 723 nm for ZnOBOPC and λ_{max} = 749 nm for ZnTtBNPC, which covers from orange light up to near-infrared light absorption (Supplementary Figure 35a-d). Relevant molecular orbitals for the four dyes were derived from DFT calculations and contributions of their transitions to the S_1 excitation were calculated from the TD-DFT method. Alkyl groups were simplified in calculations to reduce computational expense as they do not affect photophysical and

electrochemical properties. For all the four dyes, HOMO→LUMO transitions exhibit dominant 88-99% contribution to S_1 vertical excitation. As we postulated, compared to the frontier molecular orbitals of ZnOEP (Supplementary Figure 34i), ZnTtBAzP possess much lowered HOMO and LUMO levels because of the highly electron-withdrawing tetraaza-porphyrin core, while exhibiting similar λ_{max} to ZnOEP. Because the tetraaza-porphyrin core is very electro-withdrawing,[11] overall, the phenyl rings in ZnTtBPC exhibit more notable electron-donating conjugated effect rather than electron-withdrawing inductive effect. Hence, the addition of four phenyl rings moves both the HOMO and LUMO energy levels of ZnTtBPC up but moves up HOMO to a much larger extent, compared with ZnTtBAzP, thus narrowing the energy gap (Supplementary Figure 35j) and leading to 79 nm bathochromic shift (Supplementary Figure 35e,f). Indeed, as seen in Supplementary Figure 35j, bottom, the HOMO→LUMO transition exhibits partial phenyl-π to tetraaza-prophyrin-π* excitation. The installation of highly electron-donating alkyoxy groups[12] in ZnOBOPC further significantly moves up HOMO and LUMO energy levels compared to ZnTtBPC but moves up HOMO to a larger extent,[7] coupled by oxygen-n to tetraaza-prophyrin-π* excitation in addition to the partial phenyl-π to tetraaza-prophyrin-π* excitation (as seen in Figure 35k, bottom), thus narrowing the energy gap (Supplementary Figure 35k) and leading to 62 nm bathochromic shift with respect to ZnTtBPC (Supplementary Figure 35f,g). Similarly, larger aryl rings in ZnTtBNPC yield more notable electron-donating conjugated effect and lead to much more narrowed energy gap (Supplementary Figure 35l) and 88 nm bathochromic shift compared to ZnTtBPC (Supplementary Figure 35g,h).

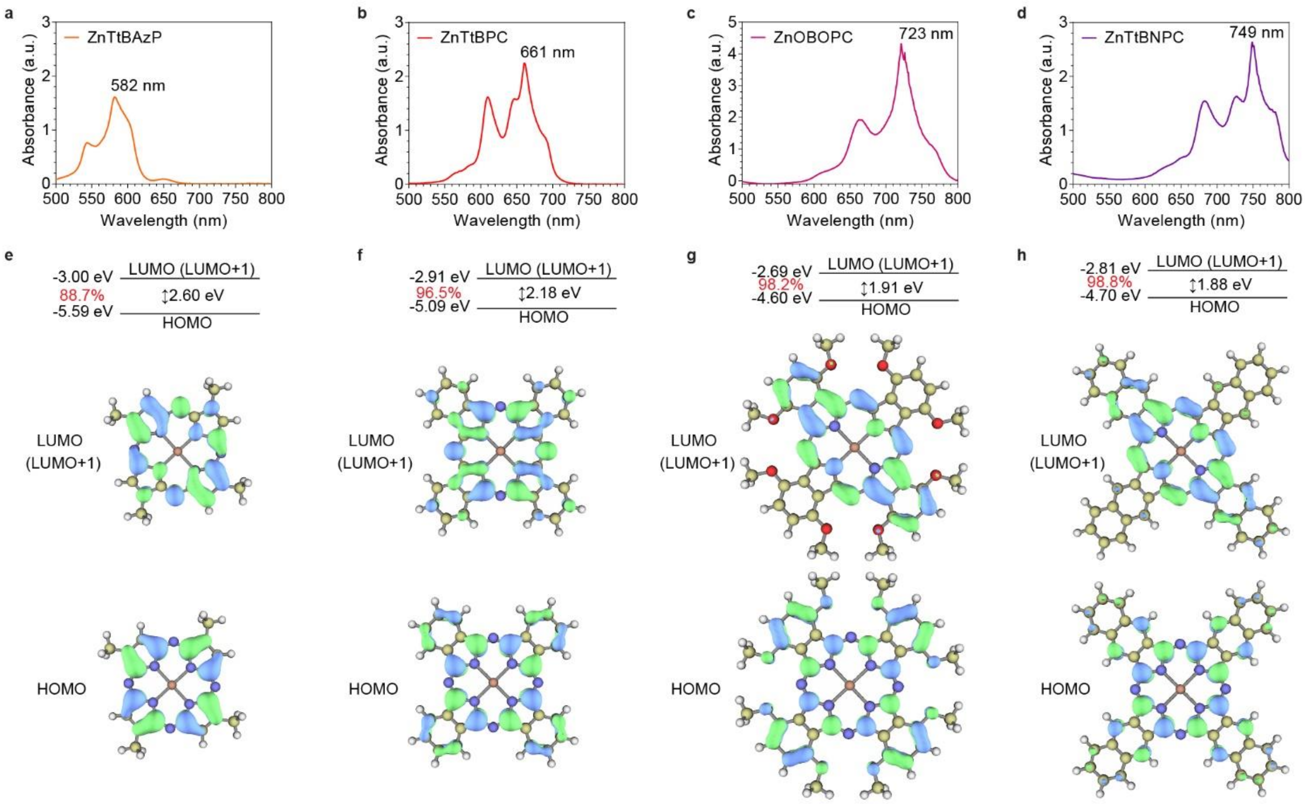

Figure 35 | Photophysical studies of PCs. a-d, UV-Vis spectra of ZnTtBAzP (**a**), ZnTtBPC (**b**), ZnOBOPC (**c**) and ZnTtBNPC (**d**) with λ_{max} denoted, with solvent DMSO/MA in 1/1 v/v and 50 ppm PC with respect to MA. **e-h**, Energy level diagrams (top) and visualisation (bottom) of HOMO, LUMO (LUMO+1, isovalue = 0.3) of ZnTtBAzP (**e**), ZnTtBPC (**f**), ZnOBOPC (**g**) and ZnTtBNPC (**h**) respectively; percentage contribution of the respective electron transition between molecular orbitals to S_1 is denoted in red.

Supplementary Note 6. Thermodynamic studies on tetraaza-porphyrin and phthalocyanine dyes

The thermodynamic calculations indicate that ZnTtBAzP, ZnTtBPC, ZnOBOPC and ZnTtBNPC are all inert for PET-RAFT polymerisation via the OQP because of inability to activate OQP-I (ΔG_{I} >16 kcal/mol, Supplementary Table 15). With respect to the RQP, interestingly, ZnTtBAzP and ZnTtBPC are ineffective for RQP-II (ΔG_{II} > 9 kcal/mol, Supplementary Table 16), whereas, ZnOBOPC and

ZnTtBNPC are ineffective for RQP-I (ΔG_I > 16 kcal/mol, Supplementary Table 16), which makes all of them inert for the RQP. Consequently, all these four PCs can only catalyse the model PET-RAFT polymerisation via the O-RQP.

Supplementary Table 15 Summary of criteria for activating the model PET-RAFT polymerisation via OQP with tetraaza-porphyrin PCs.

OQP	OQP-I	Threshold (ES-GS, kcal/mol)		OQP-II		Threshold (GS-GS, kcal/mol)	
PC	ΔG_I (kcal/mol)	Activated	Prohibited	ΔG_{II} (kcal/mol)	$\Phi_{SET,GS-GS}$	Activated	Prohibited
ZnTtBAzP	28.95	< 15	> 16	-59.93	100.0%	< 5	> 9
ZnTtBPC	23.48	< 15	> 16	-49.16	100.0%	< 5	> 9
ZnOBOPC	16.89	< 15	> 16	-37.21	100.0%	< 5	> 9
ZnTtBNPC	19.52	< 15	> 16	-39.98	100.0%	< 5	> 9

Supplementary Table 16 Summary of criteria for activating the model PET-RAFT polymerisation via RQP with tetraaza-porphyrin PCs.

RQP	RQP-I	Threshold (ES-GS, kcal/mol)		RQP-II		Threshold (GS-GS, kcal/mol)	
PC	ΔG_I (kcal/mol)	Activated	Prohibited	ΔG_{II} (kcal/mol)	$\Phi_{SET,GS-GS}$	Activated	Prohibited
ZnTtBAzP	1.11	< 14	> 16	15.87	0.00%	< 5	> 9

ZnTtBPC	9.81	< 14	> 16	12.47	0.00%	< 5	> 9
ZnOBOPC	19.74	< 14	> 16	7.90	0.13%	< 5	> 9
ZnTtBNPC	17.73	< 14	> 16	9.77	0.03%	< 5	> 9

Supplementary Note 7. Electron transfer or energy transfer?

In this section, we are to investigate the possibility of each energy transfer mechanism between the RAFT agent DTPA and the eight PCs involved in this work. As commonly accepted, energy transfer can be categorised into two main classes, i. e., radiative energy transfer and non-radiative energy transfer (which includes Förster resonance energy transfer and Dexter energy transfer). Triplet-triplet annihilation is not possible here since DTPA as the acceptor is in its singlet ground state (S_0).

7.1 Radiative energy transfer

Radiative energy transfer, also known as trivial energy transfer, requires the excited donor (D*) to emit photons which the ground state acceptor (A) is capable of absorbing.[13] In the scope of the present work, D* is the T_1 state ^{3}PC* and A is the ground state DTPA. While the S_1 state ^{1}PC* emits fluorescence, the T_1 state ^{3}PC* emits phosphorescence. The eight PCs used in this work are ZnOEP with maximum absorption wavelength corresponding to the lowest-energy absorption peak (λ_{max}) of 575 nm, ZnTPP (λ_{max} = 598 nm), TPP (λ_{max} = 645 nm), ZnTSP (λ_{max} = 660 nm), ZnTtBAzP (λ_{max} = 582 nm), ZnTtBPC (λ_{max} = 661 nm), ZnOBOPC (λ_{max} = 723 nm) and ZnTtBNPC (λ_{max} = 749 nm). Because the fluorescence emission wavelengths and phosphorescence emission wavelengths of these eight macrocyclic dyes must be longer than each corresponding λ_{max}, the photon with highest possible energy emitted from the eight dyes can only correspond to > 575 nm light. However, the λ_{max} of DTPA is measured to be ~ 420 nm (or 406 nm from TD-DFT calculations) which means the

lowest energy photon it can accept corresponds to 420 nm light, obviously too much for what the eight PCs can provide.

Therefore, this trivial energy transfer mechanism is impossible between DTPA and the eight excited macrocyclic PCs.

7.2 Förster resonance energy transfer

Förster resonance energy transfer (FRET) is also known as Coulombic energy transfer or dipole-dipole energy transfer. FRET occurs between a singlet excited donor $^1D^*$ (S_1) and a singlet ground state acceptor A (S_0), where $^1D^*$ (S_1) transfers its excess singlet excitation energy to A (S_0) through dipole-dipole interaction, without any electron exchange.[14,15] Because the FRET by dipole-dipole coupling relies on large transition dipoles (or large oscillator strength) and is only effective in singlet-singlet energy transfer, it does not involve the transfer of electron in any form. Hence, FRET is somehow similar to radiative energy transfer in that the acceptor is roughly considered vertical electronic excitation after FRET since the transfer of a "virtual photon" is almost instantaneous.[16] Unlike the trivial radiative energy transfer, which involves photon emission from $^1D^*$ (S_1) de-excitation and photon absorption from A (S_0) vertical excitation as two consecutive processes, instead, the non-irradiative energy transfer FRET through strong dipole-dipole coupling is characteristic of simultaneous $^1D^*$ (S_1) de-excitation and A (S_0) vertical excitation. Nevertheless, just like that in radiative energy transfer as stated in the previous subsection, the excited states of the eight PCs in the present work cannot provide enough energy for the vertical electronic excitation of DTPA and FRET is thus unlikely.

7.3 Dexter energy transfer

Dexter energy transfer (DET), also known as electron exchange energy transfer, or collisional energy transfer. DET always occurs via electron exchange between a triplet excited donor $^3D^*$ (T_1) and a ground state acceptor A (S_0) forming D (S_0) and $^3A^*$ (T_1) in most cases.[17] Unlike FRET which only involves transfer of a "virtual photon", DET involves actual exchange of two electrons.[17]

Therefore, the collisional DET is in some sense an analogue of electron transfer and thus has to be treated as "non-vertical"/"hot-band" energy transfer where A is non-vertically "excited" to $^3A^*$ during the process.[16] Indeed, it is widely accepted that this type of DET can be treated as electron transfer within the scope of Marcus theory[18-22] and can be analysed with similar thermodynamic strategy as described in the main text.[16] Interestingly, the ΔG_{DET} of DET processes between each pair of the $^1PC^*$ (T_1) and DTPA (S_0) are respectively found to be: ZnOEP with ΔG_{DET} = -0.6 kcal/mol, ZnTPP with ΔG_{DET} = 6.3 kcal/mol, TPP with ΔG_{DET} = 9.0 kcal/mol, ZnTSP with ΔG_{DET} = 8.0 kcal/mol, ZnTtBAzP with ΔG_{DET} = 7.6 kcal/mol, ZnTtBPC with ΔG_{DET} = 14.1 kcal/mol, ZnOBOPC with ΔG_{DET} = 19.8 kcal/mol and ZnTtBNPC with ΔG_{DET} = 9.0 kcal/mol according to (TD-)DFT calculations. Obviously, both photoinduced electron transfer (PET) discussed in the main text and DET (also known as P3T in our previous mechanistic work[23]) indicate incapability of TPP, ZnTSP, ZnTtBAzP, ZnTtBPC, ZnOBOPC and ZnTtBNPC six PCs to activate DTPA. Therefore, in terms of experimental observation, the "PET or DET" puzzle (also known as the "PET or P3T" puzzle[23]) within the $^3PC^*$-DTPA interaction is only valid for ZnOEP and ZnTPP. Experimentally, ZnOEP and ZnTPP can both efficiently activate DTPA (see main text). In the scope of PET, as substantially discussed in the main text, thermodynamic analysis for ZnOEP and ZnTPP based on PET fits excellently well with the experimental observation. However, in the scope of DET, ZnTPP exhibit a ΔG_{DET} = 6.3 kcal/mol and is exothermic. As DET is a reversible process (the back DET is also a DET), ΔG_{DET} = 6.3 kcal/mol means ZnTPP can only yield Φ_{DET} = 0.5%, which is apparently too low for effective activation, especially considering the high efficiency that ZnTPP exhibited in PET-RAFT polymerisation via the OQP. Indeed, our previous mechanistic study for ZnTPP in collaboration with Sean Smith et al. did reveal that ZnTPP activates DTPA mostly by PET rather than DET.[24] On the other hand, with respect to ZnOEP for DET, its ΔG_{DET} = -0.6 kcal/mol yielding Φ_{DET} = 62.4 % indicates that ZnOEP is theoretically likely to activate DTPA through DET to some extent.

Therefore, the activation of DTPA by ZnOEP could be a mixture of PET and DET, whereas other seven PCs including ZnTPP, TPP, ZnTSP, ZnTtBAzP, ZnTtBPC, ZnOBOPC and ZnTtBNPC are

only likely to activate DTPA mostly through PET. Although we cannot determine whether any portion of DET is mixed into the activation of DTPA by ZnOEP, it does not affect any conclusions of the present work and does not affect applications of ZnOEP in PET-RAFT polymerisation.

7.4 Proof from actual systems mechanistically investigated in our previous work

We had been collaborating with other computational research groups for years to investigate the "PET or DET" puzzle in PET-RAFT polymerisation. Collaborating with Allonas and co-workers, we combined comprehensive experimental and computational studies and discovered that $Ir(ppy)_3$ and $Ru(bpy)_3^{2+}$ are both favourable for a DET mechanism to activate common RAFT agents.[23] Indeed, referring to the deduction described above, the blue-light absorbing $Ir(ppy)_3$[25] and $Ru^{2+}(bpy)_3$[26] should have higher triplet excitation energies than all the eight orange-to-NIR-light absorbing macrocyclic PCs used in this work; thus, $Ir(ppy)_3$ and $Ru^{2+}(bpy)_3$ should be favourable for the DET mechanism to activate DTPA, which is indeed in agreement with our previous finding. At the same time, collaboration with Smith and co-workers revealed that ZnTPP can only activate DTPA through PET mechanism,[24] which is also in agreement with our deduction here.

7.5 Photoinduced electron transfer and conclusion

In conclusion, in light of the thermodynamic analysis as described in the main text and this section and our previous findings,[23,24] we can conclude that the six PCs used in this work TPP, ZnTSP, ZnTtBAzP, ZnTtBPC, ZnOBOPC and ZnTtBNPC cannot activate DTPA in the presence of appropriate light, whereas ZnTPP can activate DTPA exclusively via PET under irradiation. While it is sure that ZnOEP can activate DTPA via PET under irradiation, it is still possible for a certain portion (might be significant) of DET to occur, which is currently impossible to monitor and does not affect the conclusions or applications in any sense that we could expect.

Supplementary Note 8. Deriving thermodynamic thresholds for SET processes

8.1 Characteristics of SET processes

As described by the Marcus theory,[18-22] the reactants in an outer-sphere bimolecular SET reaction (which is the case in this work) are only weakly coupled, with an electron jumping in between, while their individuality is retained (without large structural changes). Therefore, outer-sphere bimolecular SET does not have an energy barrier as it does not involve a transition state intermediate during the process. While the two reactants approach each other with only weak coupling of their electronic orbitals, an electron can transfer between frontier molecular orbitals of the two species. Meanwhile, molecular geometries of both reactants slightly fluctuate during the SET event, toggling from the starting equilibrium to the new equilibrium after charge separation. Consequently, the rate of a SET process (or whether it should occur or not in a certain condition) is determined by the Gibbs free energy change (ΔG) of the redox reaction. Literally, ΔG of a SET process stands for the energy that the electron acceptor received by gaining an electron and toggling to the final equilibrium structure, abstracted by the energy that the electron donor released by losing an electron and toggling to the final equilibrium structure. During charge separation, solvent reorganisation and internal reorganisation can together provide a small energy barrier. However, because the reorganisation energies are very small values which do not exert notable effect in thermodynamics, we did not consider these reorganisation energies in DFT calculations to reduce computational costs.

8.2 Deriving thermodynamic thresholds for GS-GS SET processes

For a SET process between two ground state species (GS-GS SET), the forward SET and the corresponding reverse SET (RET) occur simultaneously. In this case, RET and the back SET (BET) are the same. As our main focus is how likely the SET products are produced and diffused to the solvent after the outer-sphere GS-GS SET (to be available for the next catalytic event), we define the quantum yield of the GS-GS SET process ($\Phi_{ET,GS\text{-}GS}$) as describing how likely the reversible GS-GS SET ends up with products after the outer-sphere SET event. Thus, from the statistical

perspective where a sufficient number of GS-GS SET events are sampled in a given period of time, the equilibrium concentrations of both reactants are the same ($[R_1] = [R_2] = [R]$) while those of both products are also the same ($[P_1] = [P_2] = [P]$). Consequently, $\Phi_{ET,GS-GS}$ can be expressed as:

Supplementary Equation 1 (expression of $\Phi_{ET,GS-GS}$)

$$\Phi_{\mathrm{ET,GS-GS}} = [\mathrm{P}]/([\mathrm{R}] + [\mathrm{P}]),$$

where $[R_1] = [R_2] = [R]$ is the equilibrium concentration of either reactant in the process of GS-GS SET, and $[P_1] = [P_2] = [P]$ is the equilibrium concentration of either product in the process of GS-GS-SET.

Considering the equilibrium constant *K* of the GS-GS SET reaction is:

Supplementary Equation 2 (expression of *K*)[27-29]

$$K = ([\mathrm{P_1}][\mathrm{P_2}])/([\mathrm{R_1}][\mathrm{R_2}]) = [\mathrm{P}]^2/[\mathrm{R}]^2,$$

the relation between *K* and $\Phi_{ET,GS-GS}$ is thus derived as:

Supplementary Equation 3 (expression of *K* by $\Phi_{SET,GS-GS}$)

$$K = (\Phi_{\mathrm{SET,GS-GS}}{}^{-1} - 1)^{-2}.$$

Further, as the Nernst Supplementary Equation at equilibrium is:

Supplementary Equation 4 (Nernst Supplementary Equation at equilibrium)[30,31]

$$\Delta G = -RT\ln K,$$

where R is the ideal gas constant and T is temperature,

the relation between $\Phi_{\mathrm{ET,GS-GS}}$ and ΔG can be finally obtained as:

Supplementary Equation 5 (expression of $\Phi_{\mathrm{SET,GS-GS}}$ by ΔG)

$$\Phi_{\mathrm{SET,GS-GS}} = (e^{\Delta G/(2RT)} + 1)^{-1}.$$

For instance, $\Phi_{SET,GS-GS}$ at $\Delta G = 0$ should be 50%, while lower ΔG leads to higher $\Phi_{ET,GS-GS}$. As a threshold, at room temperature 298.15 K (RT), if a GS-GS ET process has ΔG = 9 kcal/mol, $\Phi_{ET,GS-}$

$_{GS}$ is derived as 0.05%, which indicates this GS-GS SET reaction cannot generate noticeable SET products for the next-step process in a catalytic cycle at RT. On the other hand, if Δ*G* = 5 kcal/mol, $\Phi_{ET,GS\text{-}GS}$ is derived as 1.45%, which indicates noticeable generation of reactive species for the next-step process. Considering these, we can qualitatively define a GS-GS SET process with Δ*G* less than 5 kcal/mol is effective at RT, whereas any GS-GS ET process with Δ*G* greater than 9 kcal/mol should be considered prohibited at RT; the exact value of $\Phi_{ET,GS\text{-}GS}$ can be derived case by case from the exact Δ*G* of the specified GS-GS SET process.

8.3 Deriving thermodynamic thresholds for ES-GS SET (PET) processes

On the other hand, for a photoinduced SET (PET) process where SET occurs between an excited state species and a ground state species (i.e. ES-GS SET), the thermodynamic thresholds are different. Because one of the reactants in an ES-GS SET is an excited state, the process is irreversible[32] (it is impossible to regenerate an excited state from RET) and consequently always has a Φ_{PET} of 100%. Therefore, ES-GS SET is only limited by the rate of PET (i.e. whether the PET reaction is sufficiently possible to occur within the excited state lifetime). As the Marcus theory clearly suggests that thermodynamics controls the kinetics of SET processes[22,33] (based on the Arrhenius[34] and Eyring[35,36] Supplementary Equations), there must be thermodynamic thresholds for the PET process. To quantify the thermodynamic thresholds for a PET process, we introduced the Eyring Supplementary Equation under the scheme of the transition state theory,[35,36] where the quasi-equilibrium assumption assumes the transition state complex is only in quasi-equilibrium with the reactants (independent of products) in determining the rate constant of the forward reaction. Provided the aforementioned assumption that the small reorganisation energies are negligible, the rate constant of PET (k_{PET}) is determined by its ΔG as the energy barrier, and the reverse rate constant is zero (because RET is prohibited in PET). Therefore, the rate constant of PET can be expressed by the Eyring Supplementary Equation as:

Supplementary Equation 6 (expression of k_{PET} by the Eyring Supplementary Equation)

$$k_{\mathrm{PET}} = k_{\mathrm{B}} T e^{-\Delta G/(RT)}/h,$$

where k_B is the Boltzmann constant.

As aforementioned, k_{PET} should be high enough that it can allow notable product to be formed within lifetime of the excited state. Normally, T_1 has the longest lifetime (τ_T), which is usually at the ms-scale for porphyrin derivatives.[37] Therefore, to give a conservative estimation, ES-GS SET with a half-life $t_{(1/2)} = \ln 2/k_{PET} < 10$ ms (corresponding to $\Delta G < 15$ kcal/mol by solving the Eyring Supplementary Equation) can be considered efficient at RT (i.e. a notable number of PET events can proceed within T_1 lifetime), whereas PET with a $t_{(1/2)} > 100$ ms (corresponding to $\Delta G > 16$ kcal/mol) should be considered prohibited at RT (i.e. seldom does any PET events can proceed within the T_1 lifetime).

In summary, a GS-GS SET process such as process II in OQP (OQP-II), RQP-II, RQP-III and O-RQP-III (Figure 3c), should be considered efficient when its $\Delta G < 5$ kcal/mol but prohibited when $\Delta G > 9$ kcal/mol; whereas a PET process such as process OQP-I, RQP-I and O-RQP-I (Figure 3c), should be considered efficient when its $\Delta G < 15$ kcal/mol but prohibited when $\Delta G > 16$ kcal/mol. Discussions on O-RQP-II are seen as Section 10.

Supplementary Note 9. Experimental detection of $^1O_2$ and evidence of $^1O_2$-TEA reaction

We prepared a DMSO solution of ZnTSP with the same concentration, solvent and cuvette as in the polymerisation, but excluded DTPA and MA. After adding 0.5 mol/L 9,10-dimethylanthracene (DMAn) as $^1O_2$ trapper (mechanism see Supplementary Figure 36a), we placed the solution under 10 mW/cm^2 660 nm irradiation and recorded the ultraviolet-visible (UV-vis) spectra at different irradiation time (Supplementary Figure 36b). Obviously, the signal from DMAn rapidly declined within the first 30 s, indicating considerable $^1O_2$ generation in the irradiated ZnTSP-containing system. We note that there is still a debate on exactly how $^1O_2$ is generated and exists (i.e. whether as free $^1O_2$ or a (PC-O_2)* complex etc.).[38,39] As this aspect has remained in debate for decades and needs comprehensive excited state dynamics to fully investigate, we have decided to launch a series

of computation-centred projects in the near future to uncover the underlying mechanism behind the $^1O_2$ generation ability of macrocyclic PCs. However, herein, it is of certainty that the high energy species $^1O_2$ in a certain form is generated from the system which can efficiently react with DMAn; whereas its exact form or how it is formed is beyond focus of this study. Upon addition of TEA with molar ratio of [PC]:[TEA] = 0.01:0.5 to the same ZnTSP-containing system and put it under the same irradiation, we observed much slower DMAn consumption (Supplementary Figure 38) compared with the sample without TEA (Supplementary Figure 37). This evidently indicates efficient reaction between TEA and $^1O_2$, which competes with $^1O_2$ trapping by DMAn and largely retards the consumption of DMAn (Supplementary Figure 38). We further increase the content of TEA to [PC]:[TEA] = 0.01:5 in the present ZnTSP-containing system and observed no notable consumption of DMAn under irradiation within 180 s (Supplementary Figure 39), which double confirmed the ability of TEA to react with $^1O_2$.

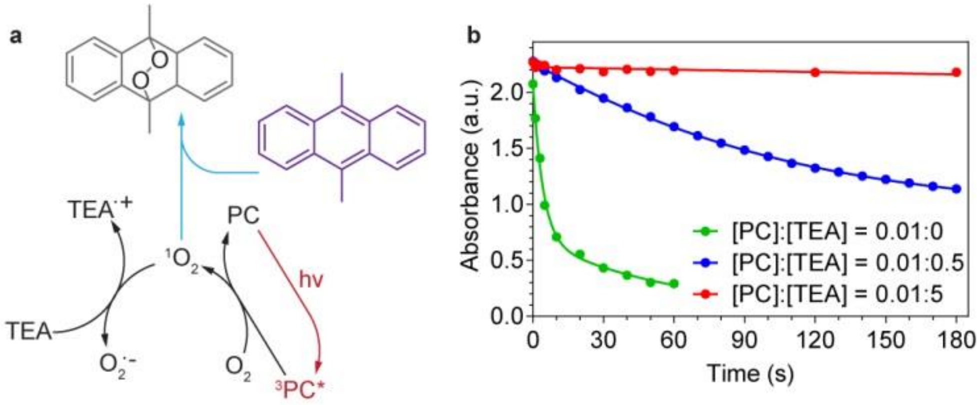

Supplementary Figure 36 a, Scheme of $^1O_2$ generation by PC, its trapping by DMAn and its reaction with TEA. **b**, Absorbance at 402 nm versus irradiation for ZnTSP-containing systems with 0.5 mol/L DMAn and TEA at different concentrations, plotted from Figures S37-39.

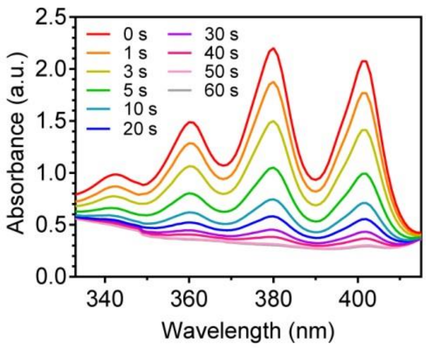

Supplementary Figure 37 UV-Vis spectrum of ZnTSP-containing systems in DMSO with 0.5 mol/L DMAn in the absence of TEA at different irradiation time.

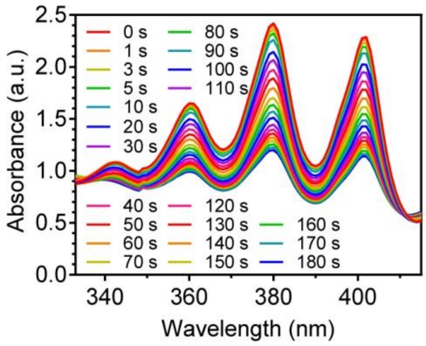

Supplementary Figure 38 UV-Vis spectrum of ZnTSP-containing systems in DMSO with 0.5 mol/L DMAn TEA at a molar ratio of [PC]:[TEA] = 0.01:0.5 at different irradiation time.

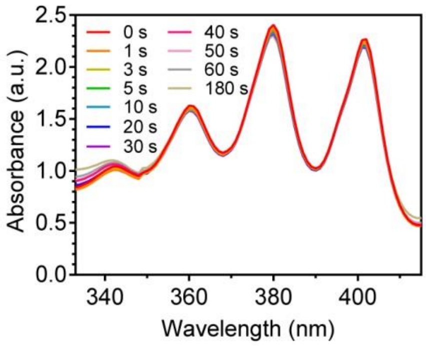

Supplementary Figure 39 UV-Vis spectrum of ZnTSP-containing systems in DMSO with 0.5 mol/L DMAn TEA at a molar ratio of [PC]:[TEA] = 0.01:5 at different irradiation time.

Supplementary Note 10. Additional discussion of O-RQP-II

The reaction between DTPA and $O_2^{\bullet -}$ (O-RQP-II) is a key process of the O-RQP-II mechanism. Considering that $O_2^{\bullet -}$ (doublet) and O_2 (triplet) bear unique electronic structures and involves complex change in multiplicity during electron transfer with DTPA, we performed DFT calculations on the bimolecular system to uncover the nature of a possible $O_2^{\bullet -}$-DTPA electron transfer. Because $O_2^{\bullet -}$ is negatively charged and the carbon centre of the trithiocarbonate is partially positively charged because of the highly electron withdrawing sulphur atoms, there is strong electrostatic interaction between $O_2^{\bullet -}$ and DTPA. To elucidate this point, unrestricted DFT (UDFT) calculations was performed. When multiplicity is 2, which corresponds to the system with DTPA and $O_2^{\bullet -}$, as shown in Supplementary Figure 40a, the distance between trithiocarbonate carbon centre (DTPA-C) and the nearest oxygen atom of $O_2^{\bullet -}$ ($O_2^{\bullet -}$-O) is only 214.9 pm, which evidently indicates strong electrostatic affinity. Indeed, Reduced Density Gradient (RDG) analysis which visualises weak interactions revealed a shoe-pad-shaped isosurface region between DTPA and $O_2^{\bullet -}$ (Supplementary Figure 40c). By zooming into the region (Supplementary Figure 40d), we clearly observed a round hole right along the C-O axis, which obviously corresponds to strong electrostatic affinity between DTPA-C and $O_2^{\bullet -}$-O, similar to an ionic bond. This means that $O_2^{\bullet -}$ can preferably be absorbed to DTPA-C and favours a possible $O_2^{\bullet -}$ to DTPA SET reaction. We then visualised the highest-lying singly occupied molecular orbital (highest SOMO) of the strongly interacting $O_2^{\bullet -}$-DTPA pair. As shown in Supplementary Figure 40a, bottom), the highest SOMO, which corresponds to the orbital where the single electron of $O_2^{\bullet -}$ is localised, has a huge portion delocalised to DTPA. This indicates significant intermolecular charge transfer (CT) from $O_2^{\bullet -}$ to DTPA when the two species approach each other. This is highly favourable for $O_2^{\bullet -}$-DTPA SET to occur. On the other hand, once the SET process is completed, $DTPA^{\bullet -}$ and O_2 are formed, with a total multiplicity of 4 (Supplementary Figure 40b). In this occasion, there is no longer any strong interaction between $DTPA^{\bullet -}$ and O_2, and O_2 is pushed away to 410.7 pm far (Supplementary Figure 40b, top). Meanwhile, we observed that the highest SOMO is solely localised on DTPA with no delocalisation to O_2. Both the repulsion

(evidenced by prolonged distance) and the localisation of highest SOMO on DTPA are unfavourable for a possible reversed SET. Therefore, O-RQP-II can be considered as a CT-assisted irreversible process that applies to the activation threshold of 15 kcal/mol. Accordingly, we calculated ΔG of O-RQP-II to be 10.76 < 15 kcal/mol, indeed favourable at room temperature.

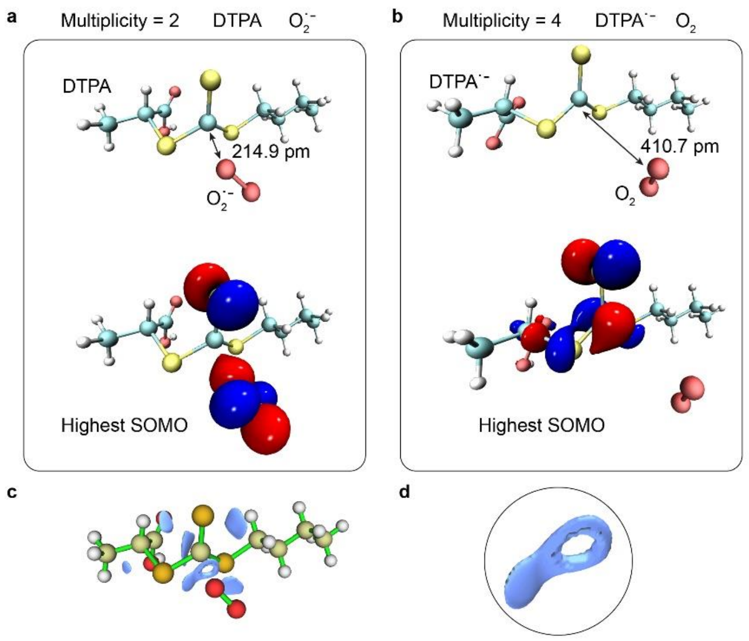

Supplementary Figure 40 a, Relaxed $O_2^{\cdot-}$-DTPA pair with multiplicity of 2. **b**, Relaxed $DTPA^{\cdot-}$-O_2 pair with multiplicity of 4. **c**, Reduced Density Gradient (RDG) analysis of the $O_2^{\cdot-}$-DTPA. **d**, Zoomed view of the RDG iso-surface between $O_2^{\cdot-}$ and DTPA.

Supplementary Note 11. Expanded scope of monomers, RAFT agents and solvents in O-RQP

An expanded scope of monomers including acrylates (MA, BzA and DEGEEA), acrylamides (DMA, DEA and NAM) and a methacrylate (MMA), RAFT agents including DTPA and CDTPA, solvents including DMSO, DMF, DMAc, Acetone, EtOAc, MeCN, MeOH, THF and Dioxane (Supplementary Figure 41) were tested with different combinations for ZnTSP-catalysed polymerisation via O-RQP in the presence of TEA and oxygen under 10 mW/cm^2 660 nm light at room temperature at a fixed molar ratio of [CTA]:[monomer]:[TEA]:[PC] = 200:1:0.5:0.01 and monomer to solvent 1:1 v/v

(Supplementary Table 17). Most of the 18 polymerizations displayed excellent correlation between experimental M_n (determined by GPC and/or NMR) and theoretical M_n values. Low $M_w/M_n < 1.2$ was observed in all cases except for BzA (M_w/M_n = 1.24), DEGEEA (M_w/M_n = 1.24) and MMA (M_w/M_n = 1.47).

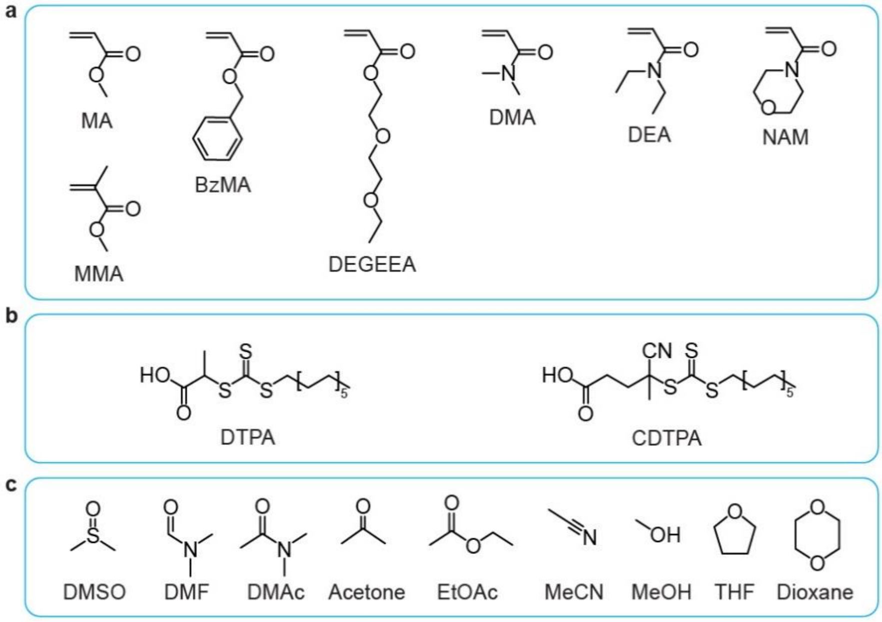

Supplementary Figure 41 Chemical structures of varied components of PET-RAFT polymerisation via O-RQP. **a**, Chemical structures of monomers. **b**, Chemical structures of RAFT agents. **b**, Chemical structures of solvents.

Supplementary Table 17 Details for polymerisation with different monomers, RAFT agents and solvents via O-RQP catalysed by ZnTSP.

Entry	Monomer	RAFT	Solvent	Time (h)	α (%)	$M_{n,theo}$ (g/mol)	$M_{n,GPC}$ (g/mol)	M_w/M_n
1	MA	DTPA	DMSO	2	78.4	13800	14500	1.12
2	BzA	DTPA	DMSO	1	88.6	29100	23500	1.24

3	DEGEEA	DTPA	DMSO	1	78.9	30100	34700	1.37
4	DMA	DTPA	DMSO	1	59.0	12000	12600	1.14
5	DEA	DTPA	DMSO	1	87.4	22600	14600	1.14
6	NAM	DTPA	DMSO	0.5	99.9	28600	20822	1.17
7	MA	CDTPA	DMSO	4	43.2	7800	8800	1.09
8	DMA	CDTPA	DMSO	3	65.1	13300	15400	1.14
9	MMA	CDTPA	DMSO	3	67.7	14000	17900	1.47
11	DMA	DTPA	DMF	2	57.4	11700	12200	1.11
12	DMA	DTPA	DMAc	2	74.4	15100	14500	1.12
13	DMA	DTPA	Acetone	2	48.1	10000	10200	1.12
14	DMA	DTPA	EtOAc	2	59.2	12100	12400	1.11
15	DMA	DTPA	MeCN	2	33.4	7000	8700	1.17
16	DMA	DTPA	MeOH	2	79.9	16200	16000	1.19
17	DMA	DTPA	THF	2	83.2	16800	16700	1.13
18	DMA	DTPA	Dioxane	2	70.0	14200	14500	1.10

Note: All polymerisations were ZnTSP-catalysed PET-RAFT polymerisation via O-RQP in the presence of TEA and oxygen under 10 mW/cm^2 660 nm light at room temperature at a fixed molar ratio of [CTA]:[monomer]:[TEA]:[PC] = 200:1:0.5:0.01 and monomer to solvent 1:1 v/v. α is monomer conversion. $M_{n,theo}$ is theoretical molecular weight. $M_{n,GPC}$ is average molecular weight determined by GPC. M_w/M_n is molecular weight dispersity.

Supplementary Note 12. Excluding the effect of alkyl groups on phthalocyanine

To demonstrate that alkyl substituents present in this work only enhance the solubility of the PCs in solvents and do not exert noticeable effect on photophysical and electrochemical properties, we further recorded the UV-vis spectrum (Supplementary Figure 42) and kinetic studies (Supplementary Figure 43-45) for ZnPC, where almost exactly the same data were obtained as compared to ZnTtBPC (main text, Figure 4f, Figure 4b and Supplementary Figure 29 respectively).

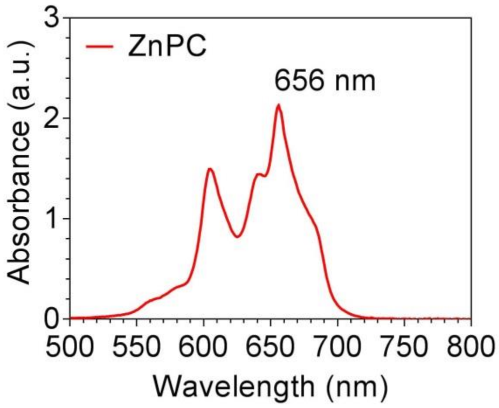

Supplementary Figure 42 UV-Vis spectra of ZnPC with λ_{max} denoted, with solvent DMSO/MA in 1/1 v/v and 50 ppm PC with respect to MA.

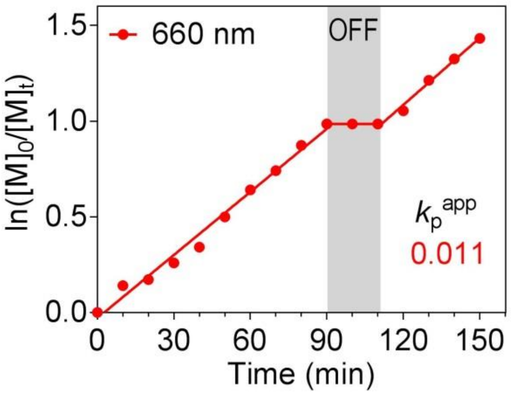

Supplementary Figure 43 Plot of $\ln([M]_0/[M]_t)$ versus time revealing k_p^{app} and temporal control for model PET-RAFT polymerisation via O-RQP catalysed by ZnPC.

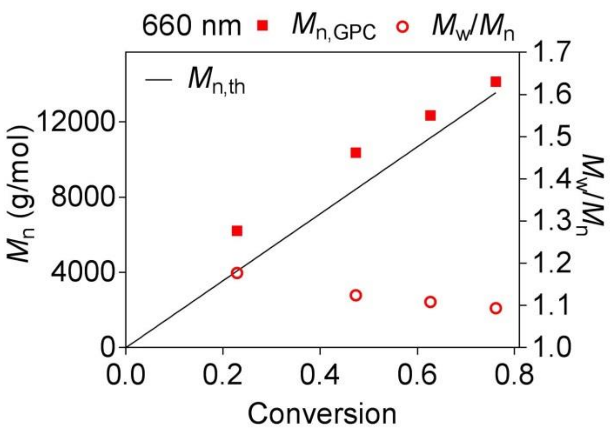

Supplementary Figure 44 M_n (solid square) and M_w/M_n (hollow circle) versus monomer conversion analysed from GPC aliquots taken at different time intervals during model PET-RAFT polymerisation via O-RQP catalysed by ZnPC.

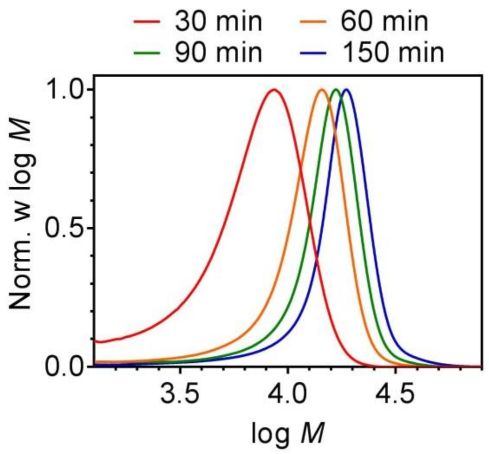

Supplementary Figure 45 Normalised molecular weight distribution of four GPC aliquots taken during ZnPC-catalysed PET-RAFT polymerisation via O-RQP, denoted as the time point when each aliquot is taken according to the corresponding kinetic plot (Supplementary Figure 43).

Supplementary Note 13. DFT calculations

13.1 Calculation for molecular geometries and energies

Geometry optimisation and energy calculation for PCs and other species at different states were performed by density functional theory (DFT) calculations with the Gaussian09 D01 software package installed in the Katana high performance computing clusters at the University of New South

Wales. Geometry optimisations were at B3LYP-GD3BJ/6-311G* level of theory (pseudopotential basis set LanL2TZ for zinc; SMD-DMSO solvation model). Single-point energy calculations were performed at B3LYP-GD3BJ/6-311+G** level of theory with the SMD-DMSO solvation model. The ball-and-stick models were presented by the CYLview software. The energy of each relaxed species at a specific state.

<u>Related molecular coordinates:</u>

Optimised coordinates for ground state ZnOEP	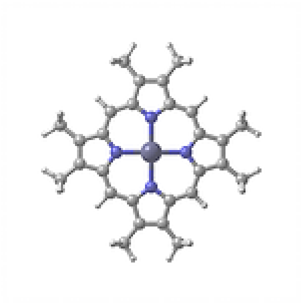
**ZnOEP, ground state**	
Experimental compound: zinc octaethyl porphyrin	
Simplified for computation: zinc octamethyl porphyrin	
B3LYP-GD3BJ; 6-311G* (C H N), LanL2TZ (Zn); solvation SMD-DMSO	
Multiplicity 1; charge 0	
Single-point energy calculated at B3LYP-GD3BJ/6-311+G** (SMD-DMSO): -3082.83955	

C	-0.68670200	4.28537200	0.00347900
C	-1.10658400	2.88798000	0.00435300
N	0.00000000	2.08648800	0.00000000
C	1.10658400	2.88798000	-0.00435300
C	0.68670200	4.28537200	-0.00347900
C	2.43063700	2.43063700	-0.00727200
C	2.88798000	1.10658400	-0.00435300
N	2.08648800	0.00000000	0.00000000
C	2.88798000	-1.10658400	0.00435300
C	4.28537200	-0.68670200	0.00347900
C	4.28537200	0.68670200	-0.00347900
C	-2.43063700	2.43063700	0.00727200
C	-2.88798000	1.10658400	0.00435300
C	-4.28537200	0.68670200	0.00347900
C	-4.28537200	-0.68670200	-0.00347900
C	-2.88798000	-1.10658400	-0.00435300
N	-2.08648800	0.00000000	0.00000000
C	-2.43063700	-2.43063700	-0.00727200
C	-1.10658400	-2.88798000	-0.00435300
C	-0.68670200	-4.28537200	-0.00347900
C	0.68670200	-4.28537200	0.00347900
C	1.10658400	-2.88798000	0.00435300
N	0.00000000	-2.08648800	0.00000000
C	2.43063700	-2.43063700	0.00727200
Zn	0.00000000	0.00000000	0.00000000
H	-3.19753500	-3.19753500	-0.01086100

H	-3.19753500	3.19753500	0.01086100
H	3.19753500	3.19753500	-0.01086100
H	3.19753500	-3.19753500	0.01086100
C	1.62050200	5.45325800	-0.00904500
H	2.28023600	5.43617700	-0.88272000
H	2.26704700	5.45596000	0.87478200
H	1.07891900	6.40034100	-0.02417000
C	-1.62050200	5.45325800	0.00904500
H	-2.28023600	5.43617700	0.88272000
H	-2.26704700	5.45596000	-0.87478200
H	-1.07891900	6.40034100	0.02417000
C	-5.45325800	1.62050200	0.00904400
H	-5.45595500	2.26705100	-0.87478000
H	-5.43618100	2.28023200	0.88272200
H	-6.40034100	1.07891900	0.02416100
C	-5.45325800	-1.62050200	-0.00904400
H	-5.45595500	-2.26705100	0.87478000
H	-5.43618100	-2.28023200	-0.88272200
H	-6.40034100	-1.07891900	-0.02416100
C	-1.62050200	-5.45325800	-0.00904500
H	-2.28023600	-5.43617700	-0.88272000
H	-2.26704700	-5.45596000	0.87478200
H	-1.07891900	-6.40034100	-0.02417000
C	1.62050200	-5.45325800	0.00904500
H	2.28023600	-5.43617700	0.88272000
H	2.26704700	-5.45596000	-0.87478200
H	1.07891900	-6.40034100	0.02417000
C	5.45325800	-1.62050200	0.00904400
H	5.45595500	-2.26705100	-0.87478000
H	5.43618100	-2.28023200	0.88272200
H	6.40034100	-1.07891900	0.02416100
C	5.45325800	1.62050200	-0.00904400
H	5.45595500	2.26705100	0.87478000
H	5.43618100	2.28023200	-0.88272200
H	6.40034100	1.07891900	-0.02416100

Optimised coordinates for T_1 state ZnOEP	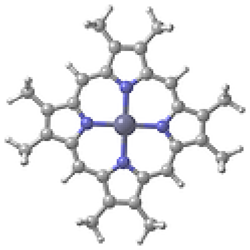
**^{3}ZnOEP*, T_1 state (UDFT)**	
Experimental compound: zinc octaethyl porphyrin	
Simplified for computation: zinc octamethyl porphyrin	
B3LYP-GD3BJ; 6-311G* (C H N), LanL2TZ (Zn); solvation SMD-DMSO	
Multiplicity 3; charge 0	
Single-point energy calculated at B3LYP-GD3BJ/6-311+G** (SMD-DMSO): -3082.773012	

C	4.33259900	0.52434600	0.00024300
C	2.94515500	1.01538100	0.00034200
N	2.08805400	-0.08287800	-0.00011400
C	2.84309300	-1.20275300	-0.00053300
C	4.27143200	-0.83705700	-0.00041200
C	2.34883800	-2.52221500	-0.00086300
C	1.00882900	-2.94442200	-0.00058600
N	-0.08031000	-2.09308000	-0.00015100
C	-1.19951700	-2.85442700	0.00044200
C	-0.85082700	-4.25053600	0.00050600
C	0.53777400	-4.30973200	-0.00038800
C	2.54958100	2.32488300	0.00068600
C	1.19951700	2.85442700	0.00044200
C	0.85082700	4.25053600	0.00050600
C	-0.53777400	4.30973200	-0.00038800
C	-1.00882900	2.94442200	-0.00058600
N	0.08031000	2.09308000	-0.00015100
C	-2.34883800	2.52221500	-0.00086300
C	-2.84309300	1.20275300	-0.00053300
C	-4.27143200	0.83705700	-0.00041200
C	-4.33259900	-0.52434600	0.00024300
C	-2.94515500	-1.01538100	0.00034200
N	-2.08805400	0.08287800	-0.00011400
C	-2.54958100	-2.32488300	0.00068600
Zn	0.00000000	0.00000000	-0.00014600
H	-3.09599900	3.30777600	-0.00121000
H	3.33831400	3.06906600	0.00104500
H	3.09599900	-3.30777600	-0.00121000
H	-3.33831400	-3.06906600	0.00104500
C	5.38648500	-1.82988100	-0.00093800
H	5.34042900	-2.48218200	-0.87957100
H	5.34113300	-2.48238600	0.87758600
H	6.36146000	-1.34080900	-0.00129700
C	5.53537000	1.40928900	0.00073600
H	5.54908500	2.06271200	0.87932400
H	5.54998500	2.06252600	-0.87798300
H	6.46118000	0.83205000	0.00128500
C	1.83279800	5.38030700	0.00154300
H	2.48592700	5.35432500	-0.87786400
H	2.48732200	5.35126100	0.87979100
H	1.32871600	6.34829800	0.00375000
C	-1.41600000	5.52050300	-0.00059900
H	-2.06507600	5.55156400	0.88153700
H	-2.07481000	5.54399600	-0.87563300
H	-0.83055100	6.44174500	-0.00777600
C	-5.38648500	1.82988100	-0.00093800
H	-5.34042900	2.48218200	-0.87957100
H	-5.34113300	2.48238600	0.87758600
H	-6.36146000	1.34080900	-0.00129700
C	-5.53537000	-1.40928900	0.00073600
H	-5.54908500	-2.06271200	0.87932400
H	-5.54998500	-2.06252600	-0.87798300

H	-6.46118000	-0.83205000	0.00128500
C	-1.83279800	-5.38030700	0.00154300
H	-2.48592700	-5.35432500	-0.87786400
H	-2.48732200	-5.35126100	0.87979100
H	-1.32871600	-6.34829800	0.00375000
C	1.41600000	-5.52050300	-0.00059900
H	2.06507600	-5.55156400	0.88153700
H	2.07481000	-5.54399600	-0.87563300
H	0.83055100	-6.44174500	-0.00777500

Optimised coordinates for ZnOEP anion radical	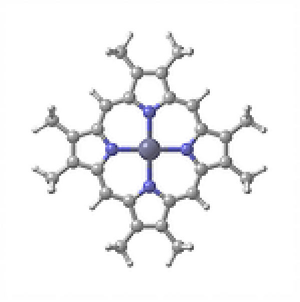
**$ZnOEP^{\bullet-}$, anion radical**	
Experimental compound: zinc octaethyl porphyrin	
Simplified for computation: zinc octamethyl porphyrin	
B3LYP-GD3BJ; 6-311G* (C H N), LanL2TZ (Zn); solvation SMD-DMSO	
Multiplicity 2; charge -1	
Single-point energy calculated at B3LYP-GD3BJ/6-311+G** (SMD-DMSO): -3082.933527	

C	0.68435000	4.29432300	0.00020800
C	1.11476100	2.89271700	0.00022200
N	0.00000000	2.08629800	0.00006000
C	-1.11448000	2.89299500	-0.00010200
C	-0.68369200	4.29451300	-0.00004100
C	-2.43045600	2.45263100	-0.00023800
C	-2.90587300	1.11044500	-0.00016000
N	-2.09841700	-0.00001400	0.00001300
C	-2.90617800	-1.11019000	0.00019700
C	-4.28113900	-0.69781100	0.00017000
C	-4.28093300	0.69846200	-0.00012900
C	2.43076300	2.45234900	0.00031500
C	2.90617800	1.11019000	0.00019700
C	4.28113900	0.69781100	0.00017000
C	4.28093300	-0.69846200	-0.00012900
C	2.90587300	-1.11044500	-0.00016000
N	2.09841700	0.00001400	0.00001300
C	2.43045600	-2.45263100	-0.00023800
C	1.11448000	-2.89299500	-0.00010200
C	0.68369200	-4.29451300	-0.00004100
C	-0.68435000	-4.29432300	0.00020800
C	-1.11476100	-2.89271700	0.00022200
N	0.00000000	-2.08629800	0.00006000
C	-2.43076300	-2.45234900	0.00031500
Zn	0.00000000	0.00000000	-0.00041800
H	3.19425500	-3.22351400	-0.00035500
H	3.19445500	3.22333700	0.00043700

H	-3.19425500	3.22351400	-0.00035500
H	-3.19445500	-3.22333700	0.00043700
C	-1.61719100	5.46328600	-0.00021000
H	-2.27213100	5.45608000	0.87800100
H	-2.27223900	5.45571600	-0.87833500
H	-1.07778400	6.41223000	-0.00045300
C	1.61826800	5.46277000	0.00042300
H	2.27324000	5.45533300	-0.87776700
H	2.27331800	5.45492000	0.87854700
H	1.07923300	6.41193500	0.00067800
C	5.45965400	1.62393000	0.00039300
H	5.46759100	2.28076700	0.87819700
H	5.46886700	2.27967200	-0.87823100
H	6.40343800	1.07411600	0.00143900
C	5.45908700	-1.62503400	-0.00027900
H	5.46647000	-2.28229800	-0.87777200
H	5.46828400	-2.28040000	0.87863600
H	6.40312900	-1.07565000	-0.00186000
C	1.61719100	-5.46328600	-0.00021000
H	2.27213100	-5.45608000	0.87800100
H	2.27223900	-5.45571600	-0.87833500
H	1.07778400	-6.41223000	-0.00045300
C	-1.61826800	-5.46277000	0.00042300
H	-2.27324000	-5.45533300	-0.87776700
H	-2.27331800	-5.45492000	0.87854700
H	-1.07923300	-6.41193500	0.00067800
C	-5.45965400	-1.62393000	0.00039300
H	-5.46759100	-2.28076700	0.87819700
H	-5.46886700	-2.27967200	-0.87823100
H	-6.40343800	-1.07411600	0.00143900
C	-5.45908700	1.62503400	-0.00027900
H	-5.46647000	2.28229800	-0.87777200
H	-5.46828400	2.28040000	0.87863600
H	-6.40312900	1.07565000	-0.00186000

Optimised coordinates for ZnOEP cation radical	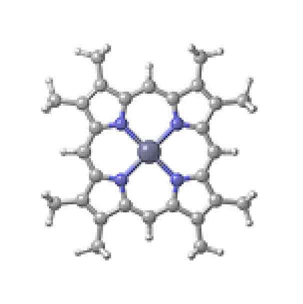
**ZnOEP$^{\bullet+}$, cation radical**	
Experimental compound: zinc octaethyl porphyrin	
Simplified for computation: zinc octamethyl porphyrin	
B3LYP-GD3BJ; 6-311G* (C H N), LanL2TZ (Zn); solvation SMD-DMSO	
Multiplicity 2; charge 1	
Single-point energy calculated at B3LYP-GD3BJ/6-311+G** (SMD-DMSO): -3082.661375	

C	2.55181700	3.51525500	0.00688700
C	1.25474200	2.81377900	0.00834900
N	1.46771000	1.46771000	0.00000000
C	2.81377900	1.25474200	-0.00834900
C	3.51525500	2.55181700	-0.00688700
C	3.43336800	0.00000000	-0.01412800
C	2.81377900	-1.25474200	-0.00834900
N	1.46771000	-1.46771000	0.00000000
C	1.25474200	-2.81377900	0.00834900
C	2.55181700	-3.51525500	0.00688700
C	3.51525500	-2.55181700	-0.00688700
C	0.00000000	3.43336800	0.01412800
C	-1.25474200	2.81377900	0.00834900
C	-2.55181700	3.51525500	0.00688700
C	-3.51525500	2.55181700	-0.00688700
C	-2.81377900	1.25474200	-0.00834900
N	-1.46771000	1.46771000	0.00000000
C	-3.43336800	0.00000000	-0.01412800
C	-2.81377900	-1.25474200	-0.00834900
C	-3.51525500	-2.55181700	-0.00688700
C	-2.55181700	-3.51525500	0.00688700
C	-1.25474200	-2.81377900	0.00834900
N	-1.46771000	-1.46771000	0.00000000
C	0.00000000	-3.43336800	0.01412800
Zn	0.00000000	0.00000000	0.00000000
H	-4.51637800	0.00000000	-0.02084900
H	0.00000000	4.51637800	0.02084900
H	4.51637800	0.00000000	-0.02084900
H	0.00000000	-4.51637800	0.02084900
C	4.99883100	2.70329300	-0.01898600
H	5.43992900	2.21190400	-0.89199100
H	5.45214300	2.24482900	0.86585200
H	5.29441500	3.75254800	-0.04133200
C	2.70329300	4.99883100	0.01898600
H	2.21190400	5.43992900	0.89199100
H	2.24482900	5.45214300	-0.86585200
H	3.75254800	5.29441500	0.04133200
C	-2.70329300	4.99883100	0.01898600
H	-2.24482900	5.45214300	-0.86585200
H	-2.21190400	5.43992900	0.89199100
H	-3.75254800	5.29441500	0.04133200
C	-4.99883100	2.70329300	-0.01898600
H	-5.45214300	2.24482900	0.86585200
H	-5.43992900	2.21190400	-0.89199100
H	-5.29441500	3.75254800	-0.04133200
C	-4.99883100	-2.70329300	-0.01898600
H	-5.43992900	-2.21190400	-0.89199100
H	-5.45214300	-2.24482900	0.86585200
H	-5.29441500	-3.75254800	-0.04133200
C	-2.70329300	-4.99883100	0.01898600
H	-2.21190400	-5.43992900	0.89199100
H	-2.24482900	-5.45214300	-0.86585200

H	-3.75254800	-5.29441500	0.04133200
C	2.70329300	-4.99883100	0.01898600
H	2.24482900	-5.45214300	-0.86585200
H	2.21190400	-5.43992900	0.89199100
H	3.75254800	-5.29441500	0.04133200
C	4.99883100	-2.70329300	-0.01898600
H	5.45214300	-2.24482900	0.86585200
H	5.43992900	-2.21190400	-0.89199100
H	5.29441500	-3.75254800	-0.04133200

Optimised coordinates for ground state ZnTPP

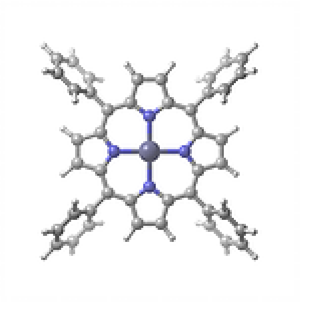

ZnTPP, ground state

Experimental compound: zinc tetraphenyl porphyrin

Simplified for computation: (no need to simplify)

B3LYP-GD3BJ; 6-311G* (C H N), LanL2TZ (Zn); solvation SMD-DMSO

Multiplicity 1; charge 0

Single-point energy calculated at B3LYP-GD3BJ/6-311+G** (SMD-DMSO): -3692.71118

C	-0.68148400	4.26915700	0.16408600
C	-1.11054100	2.89012900	0.03550300
N	0.00000000	2.09052900	-0.02745900
C	1.11054100	2.89012900	0.03550300
C	0.68148400	4.26915700	0.16408600
C	2.45218300	2.45218300	0.00000000
C	2.89012900	1.11054100	-0.03550300
N	2.09052900	0.00000000	0.02745900
C	2.89012900	-1.11054100	-0.03550300
C	4.26915700	-0.68148400	-0.16408600
C	4.26915700	0.68148400	-0.16408600
C	-2.45218300	2.45218300	0.00000000
C	-2.89012900	1.11054100	-0.03550300
C	-4.26915700	0.68148400	-0.16408600
C	-4.26915700	-0.68148400	-0.16408600
C	-2.89012900	-1.11054100	-0.03550300
N	-2.09052900	0.00000000	0.02745900
C	-2.45218300	-2.45218300	0.00000000
C	-1.11054100	-2.89012900	0.03550300
C	-0.68148400	-4.26915700	0.16408600
C	0.68148400	-4.26915700	0.16408600
C	1.11054100	-2.89012900	0.03550300
N	0.00000000	-2.09052900	-0.02745900
C	2.45218300	-2.45218300	0.00000000
C	3.50824200	3.50824200	0.00000000
C	3.62135700	4.40503800	-1.06967400
C	4.60748500	5.38875300	-1.07113700

C	5.49380600	5.49380600	0.00000000
C	5.38875300	4.60748500	1.07113700
C	4.40503800	3.62135700	1.06967400
C	-5.49380600	5.49380600	0.00000000
C	-4.60748500	5.38875300	-1.07113700
C	-3.62135700	4.40503800	-1.06967400
C	-3.50824200	3.50824200	0.00000000
C	-4.40503800	3.62135700	1.06967400
C	-5.38875300	4.60748500	1.07113700
C	3.50824200	-3.50824200	0.00000000
C	4.40503800	-3.62135700	1.06967400
C	5.38875300	-4.60748500	1.07113700
C	5.49380600	-5.49380600	0.00000000
C	4.60748500	-5.38875300	-1.07113700
C	3.62135700	-4.40503800	-1.06967400
C	-3.50824200	-3.50824200	0.00000000
C	-4.40503800	-3.62135700	1.06967400
C	-5.38875300	-4.60748500	1.07113700
C	-5.49380600	-5.49380600	0.00000000
C	-4.60748500	-5.38875300	-1.07113700
C	-3.62135700	-4.40503800	-1.06967400
H	-1.33091700	5.12561800	0.25982800
H	1.33091700	5.12561800	0.25982800
H	5.12561800	-1.33091700	-0.25982800
H	5.12561800	1.33091700	-0.25982800
H	-5.12561800	1.33091700	-0.25982800
H	-5.12561800	-1.33091700	-0.25982800
H	-1.33091700	-5.12561800	0.25982800
H	1.33091700	-5.12561800	0.25982800
H	2.93696700	4.32321400	-1.90704000
H	4.68458400	6.07144600	-1.91098000
H	6.26099200	6.26099200	0.00000000
H	6.07144600	4.68458400	1.91098000
H	4.32321400	2.93696700	1.90704000
H	-6.26099200	6.26099200	0.00000000
H	-4.68458400	6.07144600	-1.91098000
H	-2.93696700	4.32321400	-1.90704000
H	-4.32321400	2.93696700	1.90704000
H	-6.07144600	4.68458400	1.91098000
H	4.32321400	-2.93696700	1.90704000
H	6.07144600	-4.68458400	1.91098000
H	6.26099200	-6.26099200	0.00000000
H	4.68458400	-6.07144600	-1.91098000
H	2.93696700	-4.32321400	-1.90704000
H	-4.32321400	-2.93696700	1.90704000
H	-6.07144600	-4.68458400	1.91098000
H	-6.26099200	-6.26099200	0.00000000
H	-4.68458400	-6.07144600	-1.91098000
H	-2.93696700	-4.32321400	-1.90704000
Zn	0.00000000	0.00000000	0.00000000

Optimised coordinates for T_1 state ZnTPP

^{3}ZnTPP*, T_1 state (UDFT)

Experimental compound: zinc tetraphenyl porphyrin

Simplified for computation: (no need to simplify)

B3LYP-GD3BJ; 6-311G* (C H N), LanL2TZ (Zn); solvation SMD-DMSO

Multiplicity 3; charge 0

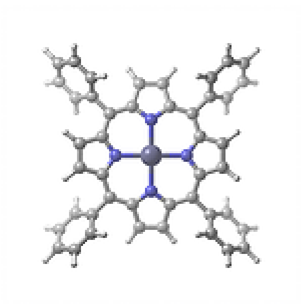

Single-point energy calculated at B3LYP-GD3BJ/6-311+G** (SMD-DMSO): -3692.655731

C	0.67967500	4.25948600	-0.34987700
C	1.11137500	2.90256600	-0.06147100
N	0.00000000	2.10903600	0.08875100
C	-1.11137500	2.90256600	-0.06147100
C	-0.67967500	4.25948600	-0.34987700
C	-2.44504600	2.47229500	0.03221200
C	-2.89281000	1.10726300	0.17051100
N	-2.10177100	0.00000000	-0.00262500
C	-2.89281000	-1.10726300	0.17051100
C	-4.21685000	-0.69408200	0.48707000
C	-4.21685000	0.69408200	0.48707000
C	2.44504600	2.47229500	0.03221200
C	2.89281000	1.10726300	0.17051100
C	4.21685000	0.69408200	0.48707000
C	4.21685000	-0.69408200	0.48707000
C	2.89281000	-1.10726300	0.17051100
N	2.10177100	0.00000000	-0.00262500
C	2.44504600	-2.47229500	0.03221200
C	1.11137500	-2.90256600	-0.06147100
C	0.67967500	-4.25948600	-0.34987700
C	-0.67967500	-4.25948600	-0.34987700
C	-1.11137500	-2.90256600	-0.06147100
N	0.00000000	-2.10903600	0.08875100
C	-2.44504600	-2.47229500	0.03221200
C	-3.50527300	3.51071900	-0.00999000
C	-3.51422100	4.56961600	0.90960900
C	-4.51069400	5.54119600	0.86468300
C	-5.50927800	5.47674900	-0.10566000
C	-5.50995800	4.42901800	-1.02702000
C	-4.52342000	3.44984900	-0.97383600
C	5.50927800	5.47674900	-0.10566000
C	4.51069400	5.54119600	0.86468300
C	3.51422100	4.56961600	0.90960900
C	3.50527300	3.51071900	-0.00999000
C	4.52342000	3.44984900	-0.97383600
C	5.50995800	4.42901800	-1.02702000
C	-3.50527300	-3.51071900	-0.00999000
C	-4.52342000	-3.44984900	-0.97383600
C	-5.50995800	-4.42901800	-1.02702000

C	-5.50927800	-5.47674900	-0.10566000
C	-4.51069400	-5.54119600	0.86468300
C	-3.51422100	-4.56961600	0.90960900
C	3.50527300	-3.51071900	-0.00999000
C	4.52342000	-3.44984900	-0.97383600
C	5.50995800	-4.42901800	-1.02702000
C	5.50927800	-5.47674900	-0.10566000
C	4.51069400	-5.54119600	0.86468300
C	3.51422100	-4.56961600	0.90960900
H	1.32899900	5.09668600	-0.55359800
H	-1.32899900	5.09668600	-0.55359800
H	-5.05336000	-1.34257300	0.69803900
H	-5.05336000	1.34257300	0.69803900
H	5.05336000	1.34257300	0.69803900
H	5.05336000	-1.34257300	0.69803900
H	1.32899900	-5.09668600	-0.55359800
H	-1.32899900	-5.09668600	-0.55359800
H	-2.74483400	4.61882900	1.67179400
H	-4.50751800	6.34754900	1.59057800
H	-6.28295300	6.23634600	-0.14335900
H	-6.28040100	4.37463900	-1.78901800
H	-4.52667800	2.63957800	-1.69378700
H	6.28295300	6.23634600	-0.14335900
H	4.50751800	6.34754900	1.59057800
H	2.74483400	4.61882900	1.67179400
H	4.52667800	2.63957800	-1.69378700
H	6.28040100	4.37463900	-1.78901800
H	-4.52667800	-2.63957800	-1.69378700
H	-6.28040100	-4.37463900	-1.78901800
H	-6.28295300	-6.23634600	-0.14335900
H	-4.50751800	-6.34754900	1.59057800
H	-2.74483400	-4.61882900	1.67179400
H	4.52667800	-2.63957800	-1.69378700
H	6.28040100	-4.37463900	-1.78901800
H	6.28295300	-6.23634600	-0.14335900
H	4.50751800	-6.34754900	1.59057800
H	2.74483400	-4.61882900	1.67179400
Zn	0.00000000	0.00000000	0.04006800

Optimised coordinates for ZnTPP anion radical	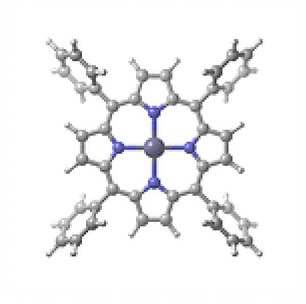
**ZnTPP$^{\bullet-}$, anion radical**	
Experimental compound: zinc tetraphenyl porphyrin	
Simplified for computation: (no need to simplify)	
B3LYP-GD3BJ; 6-311G* (C H N), LanL2TZ (Zn); solvation SMD-DMSO	
Multiplicity 2; charge -1	

Single-point energy calculated at B3LYP-GD3BJ/6-311+G** (SMD-DMSO): -3692.816602			
C	0.67912400	4.27797500	-0.14360100
C	1.11849600	2.89569500	-0.00813000
N	0.00000000	2.09166700	0.05825600
C	-1.11849600	2.89569500	-0.00813000
C	-0.67912400	4.27797500	-0.14360100
C	-2.45001200	2.47219400	0.01979500
C	-2.90623200	1.11361600	0.07009400
N	-2.10138400	0.00000000	-0.00362100
C	-2.90623200	-1.11361600	0.07009400
C	-4.26413200	-0.69262300	0.21378600
C	-4.26413200	0.69262300	0.21378600
C	2.45001200	2.47219400	0.01979500
C	2.90623200	1.11361600	0.07009400
C	4.26413200	0.69262300	0.21378600
C	4.26413200	-0.69262300	0.21378600
C	2.90623200	-1.11361600	0.07009400
N	2.10138400	0.00000000	-0.00362100
C	2.45001200	-2.47219400	0.01979500
C	1.11849600	-2.89569500	-0.00813000
C	0.67912400	-4.27797500	-0.14360100
C	-0.67912400	-4.27797500	-0.14360100
C	-1.11849600	-2.89569500	-0.00813000
N	0.00000000	-2.09166700	0.05825600
C	-2.45001200	-2.47219400	0.01979500
C	-3.50799000	3.52478400	-0.00358100
C	-3.63289700	4.44841500	1.04298100
C	-4.61637700	5.43591500	1.01509100
C	-5.49870200	5.51616000	-0.06088200
C	-5.38963300	4.60029200	-1.10777600
C	-4.40636400	3.61471900	-1.07659300
C	5.49870200	5.51616000	-0.06088200
C	4.61637700	5.43591500	1.01509100
C	3.63289700	4.44841500	1.04298100
C	3.50799000	3.52478400	-0.00358100
C	4.40636400	3.61471900	-1.07659300
C	5.38963300	4.60029200	-1.10777600
C	-3.50799000	-3.52478400	-0.00358100
C	-4.40636400	-3.61471900	-1.07659300
C	-5.38963300	-4.60029200	-1.10777600
C	-5.49870200	-5.51616000	-0.06088200
C	-4.61637700	-5.43591500	1.01509100
C	-3.63289700	-4.44841500	1.04298100
C	3.50799000	-3.52478400	-0.00358100
C	4.40636400	-3.61471900	-1.07659300
C	5.38963300	-4.60029200	-1.10777600
C	5.49870200	-5.51616000	-0.06088200
C	4.61637700	-5.43591500	1.01509100
C	3.63289700	-4.44841500	1.04298100
H	1.32678000	5.13602400	-0.24419400
H	-1.32678000	5.13602400	-0.24419400
H	-5.12271600	-1.33983300	0.31468200

H	-5.12271600	1.33983300	0.31468200
H	5.12271600	1.33983300	0.31468200
H	5.12271600	-1.33983300	0.31468200
H	1.32678000	-5.13602400	-0.24419400
H	-1.32678000	-5.13602400	-0.24419400
H	-2.95436000	4.38665200	1.88709400
H	-4.69543900	6.13951200	1.83775500
H	-6.26479300	6.28430900	-0.08410200
H	-6.06851500	4.65656700	-1.95278300
H	-4.32376100	2.90967200	-1.89677100
H	6.26479300	6.28430900	-0.08410200
H	4.69543900	6.13951200	1.83775500
H	2.95436000	4.38665200	1.88709400
H	4.32376100	2.90967200	-1.89677100
H	6.06851500	4.65656700	-1.95278300
H	-4.32376100	-2.90967200	-1.89677100
H	-6.06851500	-4.65656700	-1.95278300
H	-6.26479300	-6.28430900	-0.08410200
H	-4.69543900	-6.13951200	1.83775500
H	-2.95436000	-4.38665200	1.88709400
H	4.32376100	-2.90967200	-1.89677100
H	6.06851500	-4.65656700	-1.95278300
H	6.26479300	-6.28430900	-0.08410200
H	4.69543900	-6.13951200	1.83775500
H	2.95436000	-4.38665200	1.88709400
Zn	0.00000000	0.00000000	0.02399900

Optimised coordinates for ZnTPP cation radical

ZnTPP$^{•+}$, cation radical

Experimental compound: zinc tetraphenyl porphyrin

Simplified for computation: (no need to simplify)

B3LYP-GD3BJ; 6-311G* (C H N), LanL2TZ (Zn); solvation SMD-DMSO

Multiplicity 2; charge 1

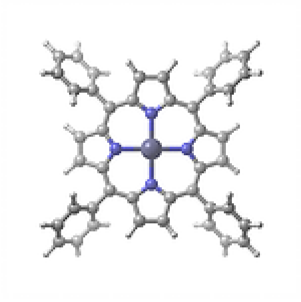

Single-point energy calculated at B3LYP-GD3BJ/6-311+G** (SMD-DMSO): -3692.529842

C	-0.68242200	4.24124700	0.39438300
C	-1.10462000	2.88897100	0.10608400
N	0.00000000	2.09948800	-0.04378000
C	1.10462000	2.88897100	0.10608400
C	0.68242200	4.24124700	0.39438300
C	2.45098300	2.45098300	0.00000000
C	2.88897100	1.10462000	-0.10608400
N	2.09948800	0.00000000	0.04378000
C	2.88897100	-1.10462000	-0.10608400
C	4.24124700	-0.68242200	-0.39438300

C	4.24124700	0.68242200	-0.39438300
C	-2.45098300	2.45098300	0.00000000
C	-2.88897100	1.10462000	-0.10608400
C	-4.24124700	0.68242200	-0.39438300
C	-4.24124700	-0.68242200	-0.39438300
C	-2.88897100	-1.10462000	-0.10608400
N	-2.09948800	0.00000000	0.04378000
C	-2.45098300	-2.45098300	0.00000000
C	-1.10462000	-2.88897100	0.10608400
C	-0.68242200	-4.24124700	0.39438300
C	0.68242200	-4.24124700	0.39438300
C	1.10462000	-2.88897100	0.10608400
N	0.00000000	-2.09948800	-0.04378000
C	2.45098300	-2.45098300	0.00000000
C	3.49955100	3.49955100	0.00000000
C	3.46930700	4.53646100	-0.94423100
C	4.45943500	5.51338200	-0.94908300
C	5.48064600	5.48064600	0.00000000
C	5.51338200	4.45943500	0.94908300
C	4.53646100	3.46930700	0.94423100
C	-5.48064600	5.48064600	0.00000000
C	-4.45943500	5.51338200	-0.94908300
C	-3.46930700	4.53646100	-0.94423100
C	-3.49955100	3.49955100	0.00000000
C	-4.53646100	3.46930700	0.94423100
C	-5.51338200	4.45943500	0.94908300
C	3.49955100	-3.49955100	0.00000000
C	4.53646100	-3.46930700	0.94423100
C	5.51338200	-4.45943500	0.94908300
C	5.48064600	-5.48064600	0.00000000
C	4.45943500	-5.51338200	-0.94908300
C	3.46930700	-4.53646100	-0.94423100
C	-3.49955100	-3.49955100	0.00000000
C	-4.53646100	-3.46930700	0.94423100
C	-5.51338200	-4.45943500	0.94908300
C	-5.48064600	-5.48064600	0.00000000
C	-4.45943500	-5.51338200	-0.94908300
C	-3.46930700	-4.53646100	-0.94423100
H	-1.33241300	5.07672600	0.60038300
H	1.33241300	5.07672600	0.60038300
H	5.07672600	-1.33241300	-0.60038300
H	5.07672600	1.33241300	-0.60038300
H	-5.07672600	1.33241300	-0.60038300
H	-5.07672600	-1.33241300	-0.60038300
H	-1.33241300	-5.07672600	0.60038300
H	1.33241300	-5.07672600	0.60038300
H	2.68065500	4.55922300	-1.68738000
H	4.43335300	6.30067100	-1.69469200
H	6.24766900	6.24766900	0.00000000
H	6.30067100	4.43335300	1.69469200
H	4.55922300	2.68065500	1.68738000
H	-6.24766900	6.24766900	0.00000000
H	-4.43335300	6.30067100	-1.69469200

H	-2.68065500	4.55922300	-1.68738000
H	-4.55922300	2.68065500	1.68738000
H	-6.30067100	4.43335300	1.69469200
H	4.55922300	-2.68065500	1.68738000
H	6.30067100	-4.43335300	1.69469200
H	6.24766900	-6.24766900	0.00000000
H	4.43335300	-6.30067100	-1.69469200
H	2.68065500	-4.55922300	-1.68738000
H	-4.55922300	-2.68065500	1.68738000
H	-6.30067100	-4.43335300	1.69469200
H	-6.24766900	-6.24766900	0.00000000
H	-4.43335300	-6.30067100	-1.69469200
H	-2.68065500	-4.55922300	-1.68738000
Zn	0.00000000	0.00000000	0.00000000

Optimised coordinates for ground state TPP

TPP, ground state

Experimental compound: tetraphenyl porphyrin

Simplified for computation: (no need to simplify)

B3LYP-GD3BJ; 6-311G* (C H N); solvation SMD-DMSO

Multiplicity 1; charge 0

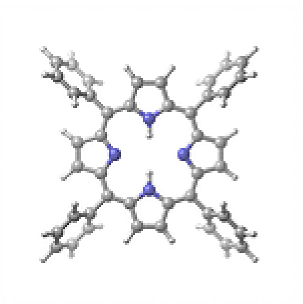

Single-point energy calculated at B3LYP-GD3BJ/6-311+G** (SMD-DMSO): -1914.458836

C	-0.68415700	4.23797100	0.17340000
C	-1.13172900	2.88312300	0.04039600
N	0.00000000	2.10277300	-0.02395100
C	1.13172900	2.88312300	0.04039600
C	0.68415700	4.23797100	0.17340000
C	2.45873600	2.43875500	-0.00974700
C	2.86050100	1.09095900	-0.06503600
N	2.03969700	0.00000000	0.00960900
C	2.86050100	-1.09095900	-0.06503600
C	4.24999300	-0.67740600	-0.22257500
C	4.24999300	0.67740600	-0.22257500
C	-2.45873600	2.43875500	-0.00974700
C	-2.86050100	1.09095900	-0.06503600
C	-4.24999300	0.67740600	-0.22257500
C	-4.24999300	-0.67740600	-0.22257500
C	-2.86050100	-1.09095900	-0.06503600
N	-2.03969700	0.00000000	0.00960900
C	-2.45873600	-2.43875500	-0.00974700
C	-1.13172900	-2.88312300	0.04039600
C	-0.68415700	-4.23797100	0.17340000
C	0.68415700	-4.23797100	0.17340000
C	1.13172900	-2.88312300	0.04039600
N	0.00000000	-2.10277300	-0.02395100

C	2.45873600	-2.43875500	-0.00974700
C	3.51915900	3.48936500	0.00177700
C	3.63977500	4.39529400	-1.05868900
C	4.63244500	5.37237000	-1.04642500
C	5.51515800	5.46050200	0.02908400
C	5.40113500	4.56435200	1.09112200
C	4.41154300	3.58452100	1.07643800
C	-5.51515800	5.46050200	0.02908400
C	-4.63244500	5.37237000	-1.04642500
C	-3.63977500	4.39529400	-1.05868900
C	-3.51915900	3.48936500	0.00177700
C	-4.41154300	3.58452100	1.07643800
C	-5.40113500	4.56435200	1.09112200
C	3.51915900	-3.48936500	0.00177700
C	4.41154300	-3.58452100	1.07643800
C	5.40113500	-4.56435200	1.09112200
C	5.51515800	-5.46050200	0.02908400
C	4.63244500	-5.37237000	-1.04642500
C	3.63977500	-4.39529400	-1.05868900
C	-3.51915900	-3.48936500	0.00177700
C	-4.41154300	-3.58452100	1.07643800
C	-5.40113500	-4.56435200	1.09112200
C	-5.51515800	-5.46050200	0.02908400
C	-4.63244500	-5.37237000	-1.04642500
C	-3.63977500	-4.39529400	-1.05868900
H	-1.33286200	5.09359600	0.26959100
H	1.33286200	5.09359600	0.26959100
H	5.09797200	-1.33485800	-0.33682700
H	5.09797200	1.33485800	-0.33682700
H	-5.09797200	1.33485800	-0.33682700
H	-5.09797200	-1.33485800	-0.33682700
H	-1.33286200	-5.09359600	0.26959100
H	1.33286200	-5.09359600	0.26959100
H	2.95811000	4.32551200	-1.89933200
H	4.71729000	6.06291400	-1.87895800
H	6.28725400	6.22262500	0.03949300
H	6.08164300	4.62857600	1.93370500
H	4.32231800	2.89105900	1.90541400
H	-6.28725400	6.22262500	0.03949300
H	-4.71729000	6.06291400	-1.87895800
H	-2.95811000	4.32551200	-1.89933200
H	-4.32231800	2.89105900	1.90541400
H	-6.08164300	4.62857600	1.93370500
H	4.32231800	-2.89105900	1.90541400
H	6.08164300	-4.62857600	1.93370500
H	6.28725400	-6.22262500	0.03949300
H	4.71729000	-6.06291400	-1.87895800
H	2.95811000	-4.32551200	-1.89933200
H	-4.32231800	-2.89105900	1.90541400
H	-6.08164300	-4.62857600	1.93370500
H	-6.28725400	-6.22262500	0.03949300
H	-4.71729000	-6.06291400	-1.87895800
H	-2.95811000	-4.32551200	-1.89933200

H	0.00000000	1.09243800	-0.08272100
H	0.00000000	-1.09243800	-0.08272100

Optimised coordinates for T_1 state TPP

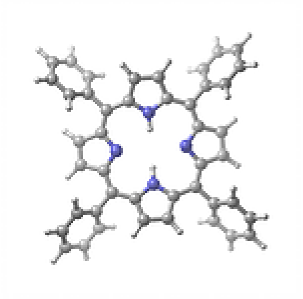

^{3}TPP*, T_1 state (UDFT)

Experimental compound: tetraphenyl porphyrin

Simplified for computation: (no need to simplify)

B3LYP-GD3BJ; 6-311G* (C H N); solvation SMD-DMSO

Multiplicity 3; charge 0

Single-point energy calculated at B3LYP-GD3BJ/6-311+G** (SMD-DMSO): -1914.402595

C	0.976334	-4.113220	0.552153
C	1.321335	-2.796097	0.201646
N	0.145266	-2.109823	0.007401
C	-0.931069	-2.946574	0.188857
C	-0.418072	-4.206404	0.543637
C	-2.311861	-2.589978	0.006304
C	-2.798955	-1.286387	-0.120930
N	-2.062791	-0.138311	0.044855
C	-2.948307	0.899660	-0.107533
C	-4.278873	0.389877	-0.433969
C	-4.186339	-0.958637	-0.443344
C	2.642815	-2.257352	0.031317
C	2.948308	-0.899669	-0.107495
C	4.278875	-0.389896	-0.433940
C	4.186341	0.958618	-0.443360
C	2.798956	1.286378	-0.120966
N	2.062790	0.138307	0.044853
C	2.311863	2.589974	0.006231
C	0.931069	2.946570	0.188769
C	0.418076	4.206364	0.543678
C	-0.976331	4.113179	0.552195
C	-1.321334	2.796091	0.201558
N	-0.145267	2.109841	0.007228
C	-2.642816	2.257347	0.031243
C	-3.273369	-3.719580	-0.033039
C	-3.126806	-4.749141	-0.974768
C	-4.035311	-5.801474	-1.021023
C	-5.094878	-5.853926	-0.115067
C	-5.242966	-4.843334	0.833471
C	-4.343082	-3.781666	0.871495

C	5.856008	-5.101008	-0.011405
C	4.809158	-5.213202	-0.926700
C	3.760910	-4.298815	-0.906689
C	3.751477	-3.243243	0.017593
C	4.809428	-3.139934	0.932312
C	5.850502	-4.063971	0.920091
C	-3.751475	3.243240	0.017554
C	-4.809417	3.139906	0.932280
C	-5.850479	4.063956	0.920110
C	-5.855984	5.101031	-0.011343
C	-4.809143	5.213251	-0.926645
C	-3.760905	4.298850	-0.906684
C	3.273369	3.719577	-0.033078
C	4.343074	3.781645	0.871467
C	5.242942	4.843327	0.833491
C	5.094847	5.853953	-0.115010
C	4.035289	5.801519	-1.020977
C	3.126798	4.749172	-0.974768
H	1.675196	-4.899338	0.788030
H	-1.009239	-5.078187	0.772998
H	-5.149669	0.988862	-0.651636
H	-4.967476	-1.667887	-0.669364
H	5.149674	-0.988887	-0.651580
H	4.967479	1.667862	-0.669399
H	1.009246	5.078118	0.773139
H	-1.675195	4.899266	0.788171
H	-2.307012	-4.711343	-1.683009
H	-3.916591	-6.581530	-1.765628
H	-5.798674	-6.678870	-0.147092
H	-6.059029	-4.881290	1.547322
H	-4.456473	-3.002303	1.616458
H	6.669812	-5.818265	-0.023782
H	4.809137	-6.014371	-1.658244
H	2.952013	-4.387389	-1.622868
H	4.803753	-2.340363	1.664300
H	6.656176	-3.974880	1.641169
H	-4.803741	2.340308	1.664241
H	-6.656145	3.974846	1.641195
H	-6.669779	5.818299	-0.023680
H	-4.809121	6.014450	-1.658156
H	-2.952013	4.387447	-1.622866
H	4.456466	3.002260	1.616407
H	6.058997	4.881270	1.547353
H	5.798630	6.678909	-0.146996
H	3.916563	6.581602	-1.765552
H	2.307008	4.711391	-1.683016
H	0.081761	-1.122587	-0.200093
H	-0.081762	1.122650	-0.200477

Optimised coordinates for TPP anion radical	

**TPP•-, anion radical**	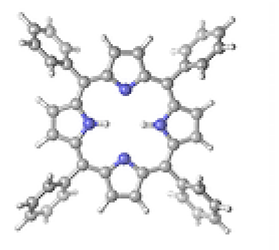
Experimental compound: tetraphenyl porphyrin	
Simplified for computation: (no need to simplify)	
B3LYP-GD3BJ; 6-311G* (C H N); solvation SMD-DMSO	
Multiplicity 2; charge -1	
Single-point energy calculated at B3LYP-GD3BJ/6-311+G** (SMD-DMSO): -1914.57149	

C	4.25942500	-0.13621200	0.68094100
C	2.89804500	-0.00651000	1.14217400
N	2.11653400	0.06071900	0.00000000
C	2.89804500	-0.00651000	-1.14217400
C	4.25942500	-0.13621200	-0.68094100
C	2.46057600	0.02829200	-2.45636200
C	1.09532900	0.09073600	-2.87869400
N	-0.00000100	0.00512600	-2.05626600
C	-1.09532000	0.09075600	-2.87870700
C	-0.68797600	0.25828500	-4.24668200
C	0.68802000	0.25832000	-4.24666800
C	2.46057600	0.02829200	2.45636200
C	1.09532900	0.09073600	2.87869400
C	0.68802000	0.25832000	4.24666800
C	-0.68797600	0.25828500	4.24668200
C	-1.09532000	0.09075600	2.87870700
N	-0.00000100	0.00512600	2.05626600
C	-2.46057400	0.02831800	2.45637600
C	-2.89803900	-0.00652700	1.14218000
C	-4.25940900	-0.13633200	0.68094000
C	-4.25940900	-0.13633200	-0.68094000
C	-2.89803900	-0.00652700	-1.14218000
N	-2.11653800	0.06069600	0.00000000
C	-2.46057400	0.02831800	-2.45637600
C	3.50622800	-0.00477700	-3.52117700
C	4.42696000	1.04111700	-3.66236600
C	5.40478200	1.00362400	-4.65511300
C	5.47702300	-0.08171000	-5.52650400
C	4.56364100	-1.12844700	-5.39858600
C	3.58698100	-1.08797500	-4.40688300
C	5.47702300	-0.08171000	5.52650400
C	5.40478200	1.00362400	4.65511300
C	4.42696000	1.04111700	3.66236600
C	3.50622800	-0.00477700	3.52117700
C	3.58698100	-1.08797500	4.40688300
C	4.56364100	-1.12844700	5.39858600
C	-3.50622900	-0.00475500	-3.52119000
C	-3.58707800	-1.08800500	-4.40682500
C	-4.56375000	-1.12846600	-5.39851900
C	-5.47704300	-0.08166100	-5.52650900
C	-5.40470600	1.00372800	-4.65519400
C	-4.42688200	1.04120400	-3.66244900

C	-3.50622900	-0.00475500	3.52119000
C	-3.58707800	-1.08800500	4.40682500
C	-4.56375000	-1.12846600	5.39851900
C	-5.47704300	-0.08166100	5.52650900
C	-5.40470600	1.00372800	4.65519400
C	-4.42688200	1.04120400	3.66244900
H	5.11768100	-0.22984500	1.32757100
H	5.11768100	-0.22984500	-1.32757100
H	-1.34175400	0.37444100	-5.09843600
H	1.34182200	0.37449200	-5.09840400
H	1.34182200	0.37449200	5.09840400
H	-1.34175400	0.37444100	5.09843600
H	-5.11765300	-0.23009600	1.32756600
H	-5.11765300	-0.23009600	-1.32756600
H	4.37028500	1.89187700	-2.99177900
H	6.10712400	1.82559100	-4.74949100
H	6.23782600	-0.11217400	-6.29958100
H	4.61410600	-1.97981000	-6.06975300
H	2.88191400	-1.90634800	-4.30929000
H	6.23782600	-0.11217400	6.29958100
H	6.10712400	1.82559100	4.74949100
H	4.37028500	1.89187700	2.99177900
H	2.88191400	-1.90634800	4.30929000
H	4.61410600	-1.97981000	6.06975300
H	-2.88209300	-1.90643900	-4.30917100
H	-4.61429200	-1.97987300	-6.06962300
H	-6.23784900	-0.11211400	-6.29958400
H	-6.10697700	1.82575000	-4.74962900
H	-4.37012700	1.89201200	-2.99193300
H	-2.88209300	-1.90643900	4.30917100
H	-4.61429200	-1.97987300	6.06962300
H	-6.23784900	-0.11211400	6.29958400
H	-6.10697700	1.82575000	4.74962900
H	-4.37012700	1.89201200	2.99193300
H	1.10642800	0.09350100	0.00000000
H	-1.10644500	0.09384600	0.00000000

Optimised coordinates for TPP cation radical	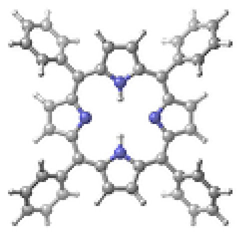
**TPP•+, cation radical**	
Experimental compound: tetraphenyl porphyrin	
Simplified for computation: (no need to simplify)	
B3LYP-GD3BJ; 6-311G* (C H N); solvation SMD-DMSO	
Multiplicity 2; charge 1	
Single-point energy calculated at B3LYP-GD3BJ/6-311+G** (SMD-DMSO): -1914.273077	

C	-0.68697600	4.17339100	0.50803300
C	-1.12503800	2.86331700	0.15315700
N	0.00000000	2.10071500	-0.02365800
C	1.12503800	2.86331700	0.15315700
C	0.68697600	4.17339100	0.50803300
C	2.46064300	2.43031500	-0.01169200
C	2.86072800	1.08765900	-0.17805500
N	2.05299700	0.00000000	-0.00369900
C	2.86072800	-1.08765900	-0.17805500
C	4.21154900	-0.67795300	-0.53155400
C	4.21154900	0.67795300	-0.53155400
C	-2.46064300	2.43031500	-0.01169200
C	-2.86072800	1.08765900	-0.17805500
C	-4.21154900	0.67795300	-0.53155400
C	-4.21154900	-0.67795300	-0.53155400
C	-2.86072800	-1.08765900	-0.17805500
N	-2.05299700	0.00000000	-0.00369900
C	-2.46064300	-2.43031500	-0.01169200
C	-1.12503800	-2.86331700	0.15315700
C	-0.68697600	-4.17339100	0.50803300
C	0.68697600	-4.17339100	0.50803300
C	1.12503800	-2.86331700	0.15315700
N	0.00000000	-2.10071500	-0.02365800
C	2.46064300	-2.43031500	-0.01169200
C	3.50701200	3.47785100	-0.00028000
C	3.44330000	4.56091800	-0.88976500
C	4.43514900	5.53578900	-0.88126700
C	5.48929700	5.45466900	0.02797600
C	5.55443000	4.38724400	0.92326600
C	4.57689300	3.39858100	0.90372500
C	-5.48929700	5.45466900	0.02797600
C	-4.43514900	5.53578900	-0.88126700
C	-3.44330000	4.56091800	-0.88976500
C	-3.50701200	3.47785100	-0.00028000
C	-4.57689300	3.39858100	0.90372500
C	-5.55443000	4.38724400	0.92326600
C	3.50701200	-3.47785100	-0.00028000
C	4.57689300	-3.39858100	0.90372500
C	5.55443000	-4.38724400	0.92326600
C	5.48929700	-5.45466900	0.02797600
C	4.43514900	-5.53578900	-0.88126700
C	3.44330000	-4.56091800	-0.88976500
C	-3.50701200	-3.47785100	-0.00028000
C	-4.57689300	-3.39858100	0.90372500
C	-5.55443000	-4.38724400	0.92326600
C	-5.48929700	-5.45466900	0.02797600
C	-4.43514900	-5.53578900	-0.88126700
C	-3.44330000	-4.56091800	-0.88976500
H	-1.33583800	4.99739900	0.75506200
H	1.33583800	4.99739900	0.75506200
H	5.03148700	-1.33581100	-0.77296600
H	5.03148700	1.33581100	-0.77296600

H	-5.03148700	1.33581100	-0.77296600
H	-5.03148700	-1.33581100	-0.77296600
H	-1.33583800	-4.99739900	0.75506200
H	1.33583800	-4.99739900	0.75506200
H	2.62978500	4.62026100	-1.60346000
H	4.38469700	6.35926200	-1.58522000
H	6.25736800	6.22049500	0.03895800
H	6.36766100	4.32428900	1.63804800
H	4.62323300	2.57429700	1.60573600
H	-6.25736800	6.22049500	0.03895800
H	-4.38469700	6.35926200	-1.58522000
H	-2.62978500	4.62026100	-1.60346000
H	-4.62323300	2.57429700	1.60573600
H	-6.36766100	4.32428900	1.63804800
H	4.62323300	-2.57429700	1.60573600
H	6.36766100	-4.32428900	1.63804800
H	6.25736800	-6.22049500	0.03895800
H	4.38469700	-6.35926200	-1.58522000
H	2.62978500	-4.62026100	-1.60346000
H	-4.62323300	-2.57429700	1.60573600
H	-6.36766100	-4.32428900	1.63804800
H	-6.25736800	-6.22049500	0.03895800
H	-4.38469700	-6.35926200	-1.58522000
H	-2.62978500	-4.62026100	-1.60346000
H	0.00000000	1.11398100	-0.24333100
H	0.00000000	-1.11398100	-0.24333100

Optimised coordinates for ground state ZnTSP

ZnTSP, ground state

Experimental compound: zinc tetra(trimethylsilylethynyl) porphyrin

Simplified for computation: zinc tetraethynyl porphyrin

B3LYP-GD3BJ; 6-311G* (C H N), LanL2TZ (Zn); solvation SMD-DMSO

Multiplicity 1; charge 0

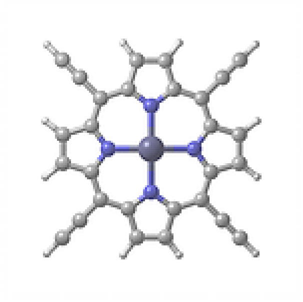

Single-point energy calculated at B3LYP-GD3BJ/6-311+G** (SMD-DMSO): -3072.841517

C	-0.68116300	-4.27329500	-0.04119300
C	-1.10199500	-2.88918600	-0.00631400
N	0.00000000	-2.08169000	0.01622200
C	1.10199500	-2.88918600	-0.00631400
C	0.68116300	-4.27329500	-0.04119300
C	2.44613500	-2.44613500	-0.01155200
C	2.88918600	-1.10199500	-0.00631400
N	2.08169000	0.00000000	0.01622200
C	2.88918600	1.10199500	-0.00631400
C	4.27329500	0.68116300	-0.04119300

C	4.27329500	-0.68116300	-0.04119300
C	-2.44613500	-2.44613500	-0.01155200
C	-2.88918600	-1.10199500	-0.00631400
C	-4.27329500	-0.68116300	-0.04119300
C	-4.27329500	0.68116300	-0.04119300
C	-2.88918600	1.10199500	-0.00631400
N	-2.08169000	0.00000000	0.01622200
C	-2.44613500	2.44613500	-0.01155200
C	-1.10199500	2.88918600	-0.00631400
C	-0.68116300	4.27329500	-0.04119300
C	0.68116300	4.27329500	-0.04119300
C	1.10199500	2.88918600	-0.00631400
N	0.00000000	2.08169000	0.01622200
H	-1.33968200	-5.12856100	-0.06729400
H	1.33968200	-5.12856100	-0.06729400
H	5.12856100	1.33968200	-0.06729400
H	5.12856100	-1.33968200	-0.06729400
H	-5.12856100	-1.33968200	-0.06729400
H	-5.12856100	1.33968200	-0.06729400
H	-1.33968200	5.12856100	-0.06729400
H	1.33968200	5.12856100	-0.06729400
Zn	0.00000000	0.00000000	0.18877600
C	2.44613500	2.44613500	-0.01155200
C	3.45498900	3.45498900	-0.04062100
C	-3.45498900	-3.45498900	-0.04062100
C	4.30737100	4.30737100	-0.06977900
C	-4.30737100	-4.30737100	-0.06977900
C	3.45498900	-3.45498900	-0.04062100
C	4.30737100	-4.30737100	-0.06977900
C	-3.45498900	3.45498900	-0.04062100
C	-4.30737100	4.30737100	-0.06977900
H	-5.06191500	5.06191500	-0.09298200
H	5.06191500	5.06191500	-0.09298200
H	5.06191500	-5.06191500	-0.09298200
H	-5.06191500	-5.06191500	-0.09298200

Optimised coordinates for T_1 state ZnTSP	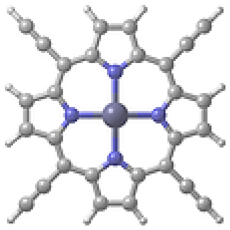
**ZnTSP, T_1 state (UDFT)**	
Experimental compound: zinc tetra(trimethylsilylethynyl) porphyrin	
Simplified for computation: zinc tetraethynyl porphyrin	
B3LYP-GD3BJ; 6-311G* (C H N), LanL2TZ (Zn); solvation SMD-DMSO	
Multiplicity 3; charge 0	
Single-point energy calculated at B3LYP-GD3BJ/6-311+G** (SMD-DMSO): -3072.789283	

C	0.69283500	4.26784800	-0.04960500
C	1.10062600	2.90623900	-0.01440700
N	0.00000000	2.09272800	0.00651600
C	-1.10062400	2.90623900	-0.01440700
C	-0.69283400	4.26784900	-0.04960500
C	-2.46796800	2.43996000	-0.01248800
C	-2.91077100	1.10226300	-0.00899000
N	-2.10095400	0.00000100	0.00374500
C	-2.91077200	-1.10226100	-0.00899000
C	-4.29819600	-0.67959400	-0.02948500
C	-4.29819500	0.67959600	-0.02948500
C	2.46796900	2.43995900	-0.01248800
C	2.91077200	1.10226100	-0.00899000
C	4.29819600	0.67959400	-0.02948500
C	4.29819500	-0.67959500	-0.02948500
C	2.91077200	-1.10226300	-0.00898900
N	2.10095400	0.00000000	0.00374500
C	2.46796800	-2.43996000	-0.01248800
C	1.10062400	-2.90623900	-0.01440700
C	0.69283300	-4.26784900	-0.04960500
C	-0.69283600	-4.26784800	-0.04960500
C	-1.10062600	-2.90623900	-0.01440700
N	-0.00000100	-2.09272800	0.00651600
H	1.34775700	5.12610200	-0.07552100
H	-1.34775500	5.12610200	-0.07552100
H	-5.15317300	-1.33884100	-0.04600300
H	-5.15317200	1.33884300	-0.04600300
H	5.15317300	1.33884100	-0.04600300
H	5.15317200	-1.33884300	-0.04600300
H	1.34775500	-5.12610200	-0.07552100
H	-1.34775700	-5.12610200	-0.07552100
Zn	0.00000000	0.00000000	0.15908900
C	-2.46796900	-2.43995900	-0.01248800
C	-3.46827600	-3.44362200	-0.02534300
C	3.46827500	3.44362200	-0.02534300
C	-4.30876600	-4.31123700	-0.03649300
C	4.30876600	4.31123800	-0.03649400
C	-3.46827400	3.44362400	-0.02534300
C	-4.30876400	4.31123900	-0.03649300
C	3.46827400	-3.44362400	-0.02534300
C	4.30876400	-4.31123900	-0.03649300
H	5.05490000	-5.07473700	-0.04668900
H	-5.05490300	-5.07473500	-0.04668900
H	-5.05490000	5.07473700	-0.04668800
H	5.05490200	5.07473500	-0.04669000

Optimised coordinates for ZnTSP anion radical	
**ZnTSP$^{\bullet-}$, anion radical**	

Experimental compound: zinc tetra(trimethylsilylethynyl) porphyrin	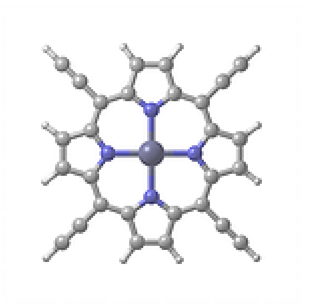
Simplified for computation: zinc tetraethynyl porphyrin	
B3LYP-GD3BJ; 6-311G* (C H N), LanL2TZ (Zn); solvation SMD-DMSO	
Multiplicity 2; charge -1	
Single-point energy calculated at B3LYP-GD3BJ/6-311+G** (SMD-DMSO): -3072.965055	

C	0.67946300	4.29160300	-0.00001400
C	1.10954300	2.90291400	-0.00000500
N	0.00000000	2.09656300	0.00000000
C	-1.10954300	2.90291400	-0.00000500
C	-0.67946300	4.29160300	-0.00001400
C	-2.44577900	2.46930800	-0.00000700
C	-2.91054100	1.10705300	-0.00000800
N	-2.10119400	0.00000000	-0.00000200
C	-2.91054100	-1.10705300	-0.00000800
C	-4.27588100	-0.69194400	-0.00001900
C	-4.27588100	0.69194400	-0.00001900
C	2.44577900	2.46930800	-0.00000700
C	2.91054100	1.10705300	-0.00000800
C	4.27588100	0.69194400	-0.00001900
C	4.27588100	-0.69194400	-0.00001900
C	2.91054100	-1.10705300	-0.00000800
N	2.10119400	0.00000000	-0.00000200
C	2.44577900	-2.46930800	-0.00000700
C	1.10954300	-2.90291400	-0.00000500
C	0.67946300	-4.29160300	-0.00001400
C	-0.67946300	-4.29160300	-0.00001400
C	-1.10954300	-2.90291400	-0.00000500
N	0.00000000	-2.09656300	0.00000000
H	1.33654300	5.14912400	-0.00002100
H	-1.33654300	5.14912400	-0.00002100
H	-5.13598100	-1.34586200	-0.00002800
H	-5.13598100	1.34586200	-0.00002800
H	5.13598100	1.34586200	-0.00002800
H	5.13598100	-1.34586200	-0.00002800
H	1.33654300	-5.14912400	-0.00002100
H	-1.33654300	-5.14912400	-0.00002100
Zn	0.00000000	0.00000000	0.00007900
C	-2.44577900	-2.46930800	-0.00000700
C	-3.45282900	-3.47649800	-0.00001300
C	3.45282900	3.47649800	-0.00001300
C	-4.32197300	-4.31605100	-0.00001900
C	4.32197300	4.31605100	-0.00001900
C	-3.45282900	3.47649800	-0.00001300
C	-4.32197300	4.31605100	-0.00001900
C	3.45282900	-3.47649800	-0.00001300
C	4.32197300	-4.31605100	-0.00001900
H	5.08515500	-5.06146100	-0.00002500

H	-5.08515500	-5.06146100	-0.00002500
H	-5.08515500	5.06146100	-0.00002500
H	5.08515500	5.06146100	-0.00002500

Optimised coordinates for ZnTSP cation radical

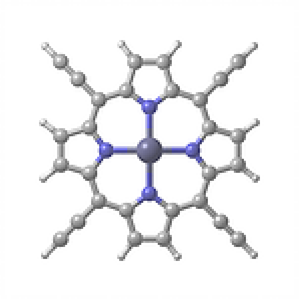

ZnTSP$^{•+}$, cation radical

Experimental compound: zinc tetra(trimethylsilylethynyl) porphyrin

Simplified for computation: zinc tetraethynyl porphyrin

B3LYP-GD3BJ; 6-311G* (C H N), LanL2TZ (Zn); solvation SMD-DMSO

Multiplicity 2; charge 1

Single-point energy calculated at B3LYP-GD3BJ/6-311+G** (SMD-DMSO): -3072.648066

C	-0.68171000	-4.27928900	-0.04469700
C	-1.09682700	-2.89624300	-0.00685600
N	0.00000000	-2.08830200	0.01729400
C	1.09682700	-2.89624300	-0.00685600
C	0.68171000	-4.27928900	-0.04469700
C	2.44445200	-2.44445200	-0.01224600
C	2.89624300	-1.09682700	-0.00685600
N	2.08830200	0.00000000	0.01729400
C	2.89624300	1.09682700	-0.00685600
C	4.27928900	0.68171000	-0.04469700
C	4.27928900	-0.68171000	-0.04469700
C	-2.44445200	-2.44445200	-0.01224600
C	-2.89624300	-1.09682700	-0.00685600
C	-4.27928900	-0.68171000	-0.04469700
C	-4.27928900	0.68171000	-0.04469700
C	-2.89624300	1.09682700	-0.00685600
N	-2.08830200	0.00000000	0.01729400
C	-2.44445200	2.44445200	-0.01224600
C	-1.09682700	2.89624300	-0.00685600
C	-0.68171000	4.27928900	-0.04469700
C	0.68171000	4.27928900	-0.04469700
C	1.09682700	2.89624300	-0.00685600
N	0.00000000	2.08830200	0.01729400
H	-1.34109000	-5.13332000	-0.07261000
H	1.34109000	-5.13332000	-0.07261000
H	5.13332000	1.34109000	-0.07261000
H	5.13332000	-1.34109000	-0.07261000
H	-5.13332000	-1.34109000	-0.07261000
H	-5.13332000	1.34109000	-0.07261000
H	-1.34109000	5.13332000	-0.07261000
H	1.34109000	5.13332000	-0.07261000
Zn	0.00000000	0.00000000	0.19691800
C	2.44445200	2.44445200	-0.01224600

C	3.44723800	3.44723800	-0.04188500
C	-3.44723800	-3.44723800	-0.04188500
C	4.29986400	4.29986400	-0.06948100
C	-4.29986400	-4.29986400	-0.06948100
C	3.44723800	-3.44723800	-0.04188500
C	4.29986400	-4.29986400	-0.06948100
C	-3.44723800	3.44723800	-0.04188500
C	-4.29986400	4.29986400	-0.06948100
H	-5.05503700	5.05503700	-0.09240600
H	5.05503700	5.05503700	-0.09240600
H	5.05503700	-5.05503700	-0.09240600
H	-5.05503700	-5.05503700	-0.09240600

Optimised coordinates for ground state ZnTtBAzP

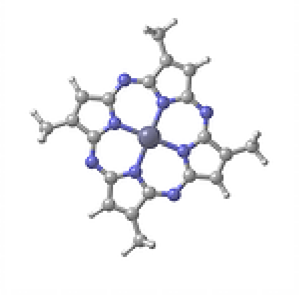

ZnTtBAzP, ground state

Experimental compound: zinc tetra(tert-butyl) tetraazaporphyrin

Simplified for computation: zinc tetrameyhyl tetraazaporphyrin

B3LYP-GD3BJ; 6-311G* (C H N), LanL2TZ (Zn); solvation SMD-DMSO

Multiplicity 1; charge 0

Single-point energy calculated at B3LYP-GD3BJ/6-311+G** (SMD-DMSO): -2989.677059

C	-2.18491400	2.06613800	0.00000000
N	-1.86234700	0.74398400	0.00000000
C	-2.99981000	0.00000000	0.00000000
C	-4.15743000	0.91558000	0.00000000
C	-3.64193400	2.17904000	0.00000000
N	-3.09962000	-1.33307900	0.00000000
C	-2.06613800	-2.18491400	0.00000000
N	-0.74398400	-1.86234700	0.00000000
C	0.00000000	-2.99981000	0.00000000
C	-0.91558000	-4.15743000	0.00000000
C	-2.17904000	-3.64193400	0.00000000
N	-1.33307900	3.09962000	0.00000000
C	0.91558000	4.15743000	0.00000000
C	2.17904000	3.64193400	0.00000000
C	2.06613800	2.18491400	0.00000000
N	0.74398400	1.86234700	0.00000000
C	0.00000000	2.99981000	0.00000000
N	3.09962000	1.33307900	0.00000000
N	1.86234700	-0.74398400	0.00000000
C	2.99981000	0.00000000	0.00000000
C	4.15743000	-0.91558000	0.00000000
C	3.64193400	-2.17904000	0.00000000
C	2.18491400	-2.06613800	0.00000000
N	1.33307900	-3.09962000	0.00000000

Zn	0.00000000	0.00000000	0.00000000
C	-5.58014600	0.47492000	0.00000000
H	-4.18612500	3.11338700	0.00000000
C	-0.47492000	-5.58014600	0.00000000
H	-3.11338700	-4.18612500	0.00000000
C	0.47492000	5.58014600	0.00000000
H	3.11338700	4.18612500	0.00000000
C	5.58014600	-0.47492000	0.00000000
H	4.18612500	-3.11338700	0.00000000
H	-6.25986200	1.32867800	0.00000000
H	-5.80321000	-0.14056000	0.87713900
H	-5.80321000	-0.14056000	-0.87713900
H	0.14056000	-5.80321000	0.87713900
H	0.14056000	-5.80321000	-0.87713900
H	-1.32867800	-6.25986200	0.00000000
H	-0.14056000	5.80321000	0.87713900
H	-0.14056000	5.80321000	-0.87713900
H	1.32867800	6.25986200	0.00000000
H	5.80321000	0.14056000	0.87713900
H	5.80321000	0.14056000	-0.87713900
H	6.25986200	-1.32867800	0.00000000

Optimised coordinates for T_1 state ZnTtBAzP

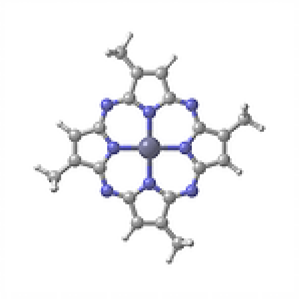

^{3}ZnTtBAzP*, T_1 state (UDFT)

Experimental compound: zinc tetra(tert-butyl) tetraazaporphyrin

Simplified for computation: zinc tetrameyhyl tetraazaporphyrin

B3LYP-GD3BJ; 6-311G* (C H N), LanL2TZ (Zn); solvation SMD-DMSO

Multiplicity 3; charge 0

Single-point energy calculated at B3LYP-GD3BJ/6-311+G** (SMD-DMSO): -2989.627702

C	2.80577400	-1.10924900	0.00000000
N	2.00973900	0.00104500	0.00000000
C	2.78959300	1.11041400	0.00000000
C	4.19673700	0.69325700	0.00000000
C	4.18695600	-0.68143700	0.00000000
N	2.37936700	2.41248200	0.00000000
C	1.11396900	2.79314800	0.00000000
N	0.00000000	2.00087500	0.00000000
C	-1.11868800	2.78153800	0.00000000
C	-0.68085000	4.21963900	0.00000000
C	0.66854800	4.20641800	0.00000000
N	2.38195100	-2.41077500	0.00000000
C	0.68085000	-4.21963900	0.00000000
C	-0.66854800	-4.20641800	0.00000000
C	-1.11396900	-2.79314800	0.00000000

N	0.00000000	-2.00087500	0.00000000
C	1.11868800	-2.78153800	0.00000000
N	-2.37936700	-2.41248200	0.00000000
N	-2.00973900	-0.00104500	0.00000000
C	-2.78959300	-1.11041400	0.00000000
C	-4.19673700	-0.69325700	0.00000000
C	-4.18695600	0.68143700	0.00000000
C	-2.80577400	1.10924900	0.00000000
N	-2.38195100	2.41077500	0.00000000
Zn	0.00000000	0.00000000	0.00000000
C	5.35442000	1.62924100	0.00000000
H	5.04084800	-1.34454200	0.00000000
C	-1.62823600	5.36368600	0.00000000
H	1.34129700	5.05285000	0.00000000
C	1.62823600	-5.36368600	0.00000000
H	-1.34129700	-5.05285000	0.00000000
C	-5.35442000	-1.62924100	0.00000000
H	-5.04084800	1.34454200	0.00000000
H	6.30227200	1.08827500	0.00000000
H	5.33344000	2.28469600	0.87708000
H	5.33344000	2.28469600	-0.87708000
H	-2.28262500	5.32651100	0.87683200
H	-2.28262500	5.32651100	-0.87683200
H	-1.10076300	6.31863500	0.00000000
H	2.28262500	-5.32651100	0.87683200
H	2.28262500	-5.32651100	-0.87683200
H	1.10076300	-6.31863500	0.00000000
H	-5.33344000	-2.28469600	0.87708000
H	-5.33344000	-2.28469600	-0.87708000
H	-6.30227200	-1.08827500	0.00000000

Optimised coordinates for ZnTtBAzP anion radical

ZnTtBAzP$^{\bullet -}$, anion radical

Experimental compound: zinc tetra(tert-butyl) tetraazaporphyrin

Simplified for computation: zinc tetrameyhyl tetraazaporphyrin

B3LYP-GD3BJ; 6-311G* (C H N), LanL2TZ (Zn); solvation SMD-DMSO

Multiplicity 2; charge -1

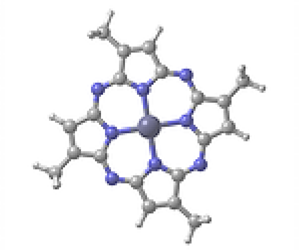

Single-point energy calculated at B3LYP-GD3BJ/6-311+G** (SMD-DMSO): -2989.805477

C	-2.94848100	0.65496900	0.00000000
N	-1.98791800	-0.31657000	0.00000000
C	-2.59089300	-1.54391100	0.00000000
C	-4.02198300	-1.35674600	0.00000000
C	-4.23371300	0.01573100	0.00000000
N	-1.97224800	-2.76040200	0.00000000

C	-0.66177500	-2.94102700	0.00000000
N	0.31649600	-1.98144900	0.00000000
C	1.54619300	-2.57978200	0.00000000
C	1.34329800	-4.04925200	0.00000000
C	0.00000000	-4.25342700	0.00000000
N	-2.73635000	2.00562500	0.00000000
C	-1.34329800	4.04925200	0.00000000
C	0.00000000	4.25342700	0.00000000
C	0.66177500	2.94102700	0.00000000
N	-0.31649600	1.98144900	0.00000000
C	-1.54619300	2.57978200	0.00000000
N	1.97224800	2.76040200	0.00000000
N	1.98791800	0.31657000	0.00000000
C	2.59089300	1.54391100	0.00000000
C	4.02198300	1.35674600	0.00000000
C	4.23371300	-0.01573100	0.00000000
C	2.94848100	-0.65496900	0.00000000
N	2.73635000	-2.00562500	0.00000000
Zn	0.00000000	0.00000000	0.00000000
C	-5.03752600	-2.45379100	0.00000000
H	-5.18645900	0.52952100	0.00000000
C	2.45227000	-5.04459700	0.00000000
H	-0.52311900	-5.20080600	0.00000000
C	-2.45227000	5.04459700	0.00000000
H	0.52311900	5.20080600	0.00000000
C	5.03752600	2.45379100	0.00000000
H	5.18645900	-0.52952100	0.00000000
H	-6.05388900	-2.05305100	0.00000000
H	-4.93400300	-3.10179000	0.87720400
H	-4.93400300	-3.10179000	-0.87720400
H	3.09583500	-4.91871100	0.87679900
H	3.09583500	-4.91871100	-0.87679900
H	2.07111700	-6.06769600	0.00000000
H	-3.09583500	4.91871100	0.87679900
H	-3.09583500	4.91871100	-0.87679900
H	-2.07111700	6.06769600	0.00000000
H	4.93400300	3.10179000	0.87720400
H	4.93400300	3.10179000	-0.87720400
H	6.05388900	2.05305100	0.00000000

Optimised coordinates for ZnTtBAzP cation radical	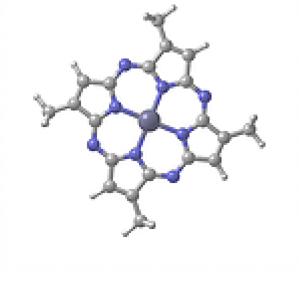
**ZnTtBAzP$^{\bullet+}$, cation radical**	
Experimental compound: zinc tetra(tert-butyl) tetraazaporphyrin	
Simplified for computation: zinc tetrameyhyl tetraazaporphyrin	
B3LYP-GD3BJ; 6-311G* (C H N), LanL2TZ (Zn); solvation SMD-DMSO	

Multiplicity 2; charge 1				
Single-point energy calculated at B3LYP-GD3BJ/6-311+G** (SMD-DMSO): -2989.478434				
C	-2.18227400	2.06263200	0.00000000	
N	-1.85784100	0.74185700	0.00000000	
C	-2.98843400	0.00000000	0.00000000	
C	-4.16357100	0.92855200	0.00000000	
C	-3.64842000	2.18226500	0.00000000	
N	-3.09616000	-1.33062300	0.00000000	
C	-2.06263200	-2.18227400	0.00000000	
N	-0.74185700	-1.85784100	0.00000000	
C	0.00000000	-2.98843400	0.00000000	
C	-0.92855200	-4.16357100	0.00000000	
C	-2.18226500	-3.64842000	0.00000000	
N	-1.33062300	3.09616000	0.00000000	
C	0.92855200	4.16357100	0.00000000	
C	2.18226500	3.64842000	0.00000000	
C	2.06263200	2.18227400	0.00000000	
N	0.74185700	1.85784100	0.00000000	
C	0.00000000	2.98843400	0.00000000	
N	3.09616000	1.33062300	0.00000000	
N	1.85784100	-0.74185700	0.00000000	
C	2.98843400	0.00000000	0.00000000	
C	4.16357100	-0.92855200	0.00000000	
C	3.64842000	-2.18226500	0.00000000	
C	2.18227400	-2.06263200	0.00000000	
N	1.33062300	-3.09616000	0.00000000	
Zn	0.00000000	0.00000000	0.00000000	
C	-5.57474800	0.47052700	0.00000000	
H	-4.18302400	3.12098000	0.00000000	
C	-0.47052700	-5.57474800	0.00000000	
H	-3.12098000	-4.18302400	0.00000000	
C	0.47052700	5.57474800	0.00000000	
H	3.12098000	4.18302400	0.00000000	
C	5.57474800	-0.47052700	0.00000000	
H	4.18302400	-3.12098000	0.00000000	
H	-6.26624600	1.31357700	0.00000000	
H	-5.78055100	-0.15139700	0.87688600	
H	-5.78055100	-0.15139700	-0.87688600	
H	0.15139700	-5.78055100	0.87688600	
H	0.15139700	-5.78055100	-0.87688600	
H	-1.31357700	-6.26624600	0.00000000	
H	-0.15139700	5.78055100	0.87688600	
H	-0.15139700	5.78055100	-0.87688600	
H	1.31357700	6.26624600	0.00000000	
H	5.78055100	0.15139700	0.87688600	
H	5.78055100	0.15139700	-0.87688600	
H	6.26624600	-1.31357700	0.00000000	

Optimised coordinates for ground state ZnTtBPC	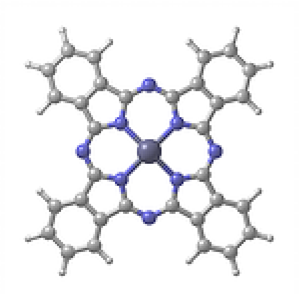
**ZnTtBPC, ground state**	
Experimental compound: zinc tetra(tert-butyl) phthylocyanine	
Simplified for computation: zinc phthylocyanine	
B3LYP-GD3BJ; 6-311G* (C H N), LanL2TZ (Zn); solvation SMD-DMSO	
Multiplicity 1; charge 0	
Single-point energy calculated at B3LYP-GD3BJ/6-311+G** (SMD-DMSO): -3447.173486	

C	2.76650700	1.18235000	0.01953300
N	1.42470100	1.42470100	0.06819100
C	1.18235000	2.76650700	0.01953300
C	2.46772100	3.46648900	-0.04738800
C	3.46648900	2.46772100	-0.04738800
N	0.00000000	3.37815200	0.00933000
C	-1.18235000	2.76650700	0.01953300
N	-1.42470100	1.42470100	0.06819100
C	-2.76650700	1.18235000	0.01953300
C	-3.46648900	2.46772100	-0.04738800
C	-2.46772100	3.46648900	-0.04738800
N	3.37815200	0.00000000	0.00933000
C	3.46648900	-2.46772100	-0.04738800
C	2.46772100	-3.46648900	-0.04738800
C	1.18235000	-2.76650700	0.01953300
N	1.42470100	-1.42470100	0.06819100
C	2.76650700	-1.18235000	0.01953300
N	0.00000000	-3.37815200	0.00933000
N	-1.42470100	-1.42470100	0.06819100
C	-1.18235000	-2.76650700	0.01953300
C	-2.46772100	-3.46648900	-0.04738800
C	-3.46648900	-2.46772100	-0.04738800
C	-2.76650700	-1.18235000	0.01953300
N	-3.37815200	0.00000000	0.00933000
Zn	0.00000000	0.00000000	0.51046600
C	4.81584700	-2.80417900	-0.11201000
C	5.14916400	-4.15620000	-0.16918500
C	4.15620000	-5.14916400	-0.16918500
C	2.80417900	-4.81584700	-0.11201000
C	-4.81584700	-2.80417900	-0.11201000
C	-5.14916400	-4.15620000	-0.16918500
C	-4.15620000	-5.14916400	-0.16918500
C	-2.80417900	-4.81584700	-0.11201000
C	-2.80417900	4.81584700	-0.11201000
C	-4.15620000	5.14916400	-0.16918500
C	-5.14916400	4.15620000	-0.16918500
C	-4.81584700	2.80417900	-0.11201000
C	4.81584700	2.80417900	-0.11201000
C	5.14916400	4.15620000	-0.16918500

C	4.15620000	5.14916400	-0.16918500
C	2.80417900	4.81584700	-0.11201000
H	5.58268600	-2.03738600	-0.11562500
H	2.03738600	-5.58268600	-0.11562500
H	-5.58268600	-2.03738600	-0.11562500
H	-2.03738600	-5.58268600	-0.11562500
H	-2.03738600	5.58268600	-0.11562500
H	-5.58268600	2.03738600	-0.11562500
H	5.58268600	2.03738600	-0.11562500
H	2.03738600	5.58268600	-0.11562500
H	-4.44927400	6.19241600	-0.21565400
H	-6.19241600	4.44927400	-0.21565400
H	4.44927400	6.19241600	-0.21565400
H	6.19241600	4.44927400	-0.21565400
H	6.19241600	-4.44927400	-0.21565400
H	4.44927400	-6.19241600	-0.21565400
H	-4.44927400	-6.19241600	-0.21565400
H	-6.19241600	-4.44927400	-0.21565400

Optimised coordinates for T_1 state ZnTtBPC

^{3}ZnTtBPC*, T_1 state

Experimental compound: zinc tetra(tert-butyl) phthylocyanine

Simplified for computation: zinc phthylocyanine

B3LYP-GD3BJ; 6-311G* (C H N), LanL2TZ (Zn); solvation SMD-DMSO

Multiplicity 3; charge 0

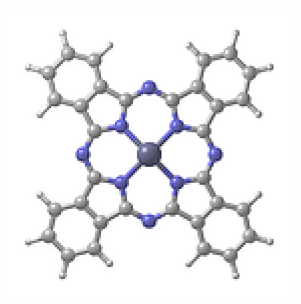

Single-point energy calculated at B3LYP-GD3BJ/6-311+G** (SMD-DMSO): -3447.132566

C	2.77957500	1.18248200	0.01617300
N	1.41387600	1.43063500	0.06483100
C	1.17945600	2.75946600	0.01664100
C	2.47181000	3.46271200	-0.04945200
C	3.47283700	2.46654900	-0.04791000
N	0.00000000	3.39834200	0.01119400
C	-1.17945600	2.75946600	0.01664100
N	-1.41387600	1.43063500	0.06483100
C	-2.77957500	1.18248200	0.01617300
C	-3.47283700	2.46654900	-0.04791000
C	-2.47181000	3.46271200	-0.04945200
N	3.38381300	0.00000000	0.00305400
C	3.47283700	-2.46654900	-0.04791000
C	2.47181000	-3.46271200	-0.04945200
C	1.17945600	-2.75946600	0.01664100
N	1.41387600	-1.43063500	0.06483100
C	2.77957500	-1.18248200	0.01617300
N	0.00000000	-3.39834200	0.01119400

N	-1.41387600	-1.43063500	0.06483100
C	-1.17945600	-2.75946600	0.01664100
C	-2.47181000	-3.46271200	-0.04945200
C	-3.47283700	-2.46654900	-0.04791000
C	-2.77957500	-1.18248200	0.01617300
N	-3.38381300	0.00000000	0.00305400
Zn	0.00000000	0.00000000	0.51206300
C	4.81918900	-2.81132900	-0.11009800
C	5.14576900	-4.16829300	-0.16536400
C	4.15134300	-5.15522200	-0.16544800
C	2.79754200	-4.81033500	-0.11068000
C	-4.81918900	-2.81132900	-0.11009800
C	-5.14576900	-4.16829300	-0.16536400
C	-4.15134300	-5.15522200	-0.16544800
C	-2.79754200	-4.81033500	-0.11068000
C	-2.79754200	4.81033500	-0.11068000
C	-4.15134300	5.15522200	-0.16544800
C	-5.14576900	4.16829300	-0.16536400
C	-4.81918900	2.81132900	-0.11009800
C	4.81918900	2.81132900	-0.11009800
C	5.14576900	4.16829300	-0.16536400
C	4.15134300	5.15522200	-0.16544800
C	2.79754200	4.81033500	-0.11068000
H	5.59168800	-2.05028300	-0.11319700
H	2.02538800	-5.57171100	-0.11428300
H	-5.59168800	-2.05028300	-0.11319700
H	-2.02538800	-5.57171100	-0.11428300
H	-2.02538800	5.57171100	-0.11428300
H	-5.59168800	2.05028300	-0.11319700
H	5.59168800	2.05028300	-0.11319700
H	2.02538800	5.57171100	-0.11428300
H	-4.43735400	6.20029000	-0.21005100
H	-6.18814800	4.46482900	-0.20979600
H	4.43735400	6.20029000	-0.21005100
H	6.18814800	4.46482900	-0.20979600
H	6.18814800	-4.46482900	-0.20979600
H	4.43735400	-6.20029000	-0.21005100
H	-4.43735400	-6.20029000	-0.21005100
H	-6.18814800	-4.46482900	-0.20979600

Optimised coordinates for ZnTtBPC anion radical	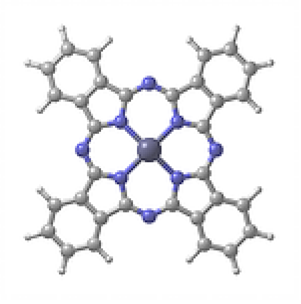
**ZnTtBPC$^{\bullet-}$, anion radical**	
Experimental compound: zinc tetra(tert-butyl) phthylocyanine	
Simplified for computation: zinc phthylocyanine	
B3LYP-GD3BJ; 6-311G* (C H N), LanL2TZ (Zn); solvation SMD-DMSO	

Multiplicity 2; charge -1				
Single-point energy calculated at B3LYP-GD3BJ/6-311+G** (SMD-DMSO): -3447.296478				
C	2.77227300	1.18459900	0.01127200	
N	1.43827300	1.42312600	0.04611800	
C	1.18664400	2.78582800	0.01170700	
C	2.46549000	3.47546800	-0.03688100	
C	3.46767200	2.47173800	-0.03804000	
N	0.00000000	3.38555900	0.00234600	
C	-1.18664400	2.78582800	0.01170700	
N	-1.43827300	1.42312600	0.04611800	
C	-2.77227300	1.18459900	0.01127200	
C	-3.46767200	2.47173800	-0.03804000	
C	-2.46549000	3.47546800	-0.03688100	
N	3.40407800	0.00000000	0.00648600	
C	3.46767200	-2.47173800	-0.03804000	
C	2.46549000	-3.47546800	-0.03688100	
C	1.18664400	-2.78582800	0.01170700	
N	1.43827300	-1.42312600	0.04611800	
C	2.77227300	-1.18459900	0.01127200	
N	0.00000000	-3.38555900	0.00234600	
N	-1.43827300	-1.42312600	0.04611800	
C	-1.18664400	-2.78582800	0.01170700	
C	-2.46549000	-3.47546800	-0.03688100	
C	-3.46767200	-2.47173800	-0.03804000	
C	-2.77227300	-1.18459900	0.01127200	
N	-3.40407800	0.00000000	0.00648600	
Zn	0.00000000	0.00000000	0.40840300	
C	4.81986700	-2.80897100	-0.08664000	
C	5.16318700	-4.15745100	-0.12991800	
C	4.16968300	-5.15491700	-0.12987300	
C	2.81850800	-4.82683400	-0.08627400	
C	-4.81986700	-2.80897100	-0.08664000	
C	-5.16318700	-4.15745100	-0.12991800	
C	-4.16968300	-5.15491700	-0.12987300	
C	-2.81850800	-4.82683400	-0.08627400	
C	-2.81850800	4.82683400	-0.08627400	
C	-4.16968300	5.15491700	-0.12987300	
C	-5.16318700	4.15745100	-0.12991800	
C	-4.81986700	2.80897100	-0.08664000	
C	4.81986700	2.80897100	-0.08664000	
C	5.16318700	4.15745100	-0.12991800	
C	4.16968300	5.15491700	-0.12987300	
C	2.81850800	4.82683400	-0.08627400	
H	5.58312300	-2.03769200	-0.08944800	
H	2.05691100	-5.59973900	-0.08901000	
H	-5.58312300	-2.03769200	-0.08944800	
H	-2.05691100	-5.59973900	-0.08901000	
H	-2.05691100	5.59973900	-0.08901000	
H	-5.58312300	2.03769200	-0.08944800	
H	5.58312300	2.03769200	-0.08944800	
H	2.05691100	5.59973900	-0.08901000	

H	-4.46656800	6.19815300	-0.16517800
H	-6.20814700	4.44708700	-0.16523700
H	4.46656800	6.19815300	-0.16517800
H	6.20814700	4.44708700	-0.16523700
H	6.20814700	-4.44708700	-0.16523700
H	4.46656800	-6.19815300	-0.16517800
H	-4.46656800	-6.19815300	-0.16517800
H	-6.20814700	-4.44708700	-0.16523700

Optimised coordinates for ZnTtBPC cation radical

ZnTtBPC$^{•+}$, cation radical

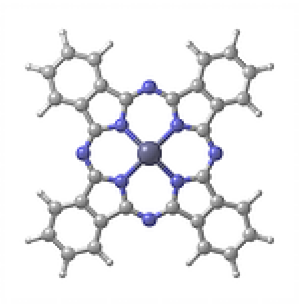

Experimental compound: zinc tetra(tert-butyl) phthylocyanine

Simplified for computation: zinc phthylocyanine

B3LYP-GD3BJ; 6-311G* (C H N), LanL2TZ (Zn); solvation SMD-DMSO

Multiplicity 2; charge 1

Single-point energy calculated at B3LYP-GD3BJ/6-311+G** (SMD-DMSO): -3446.992024

C	2.75947200	1.17947400	0.03176700
N	1.41918700	1.41918700	0.09677400
C	1.17947400	2.75947200	0.03176700
C	2.46744300	3.46360300	-0.05442700
C	3.46360300	2.46744300	-0.05442700
N	0.00000000	3.37520600	0.01848300
C	-1.17947400	2.75947200	0.03176700
N	-1.41918700	1.41918700	0.09677400
C	-2.75947200	1.17947400	0.03176700
C	-3.46360300	2.46744300	-0.05442700
C	-2.46744300	3.46360300	-0.05442700
N	3.37520600	0.00000000	0.01848300
C	3.46360300	-2.46744300	-0.05442700
C	2.46744300	-3.46360300	-0.05442700
C	1.17947400	-2.75947200	0.03176700
N	1.41918700	-1.41918700	0.09677400
C	2.75947200	-1.17947400	0.03176700
N	0.00000000	-3.37520600	0.01848300
N	-1.41918700	-1.41918700	0.09677400
C	-1.17947400	-2.75947200	0.03176700
C	-2.46744300	-3.46360300	-0.05442700
C	-3.46360300	-2.46744300	-0.05442700
C	-2.75947200	-1.17947400	0.03176700
N	-3.37520600	0.00000000	0.01848300
Zn	0.00000000	0.00000000	0.58276200
C	4.80803500	-2.79516400	-0.13481500
C	5.14137400	-4.15294800	-0.20664600
C	4.15294800	-5.14137400	-0.20664600

C	2.79516400	-4.80803500	-0.13481500
C	-4.80803500	-2.79516400	-0.13481500
C	-5.14137400	-4.15294800	-0.20664600
C	-4.15294800	-5.14137400	-0.20664600
C	-2.79516400	-4.80803500	-0.13481500
C	-2.79516400	4.80803500	-0.13481500
C	-4.15294800	5.14137400	-0.20664600
C	-5.14137400	4.15294800	-0.20664600
C	-4.80803500	2.79516400	-0.13481500
C	4.80803500	2.79516400	-0.13481500
C	5.14137400	4.15294800	-0.20664600
C	4.15294800	5.14137400	-0.20664600
C	2.79516400	4.80803500	-0.13481500
H	5.57432100	-2.02861100	-0.13929700
H	2.02861100	-5.57432100	-0.13929700
H	-5.57432100	-2.02861100	-0.13929700
H	-2.02861100	-5.57432100	-0.13929700
H	-2.02861100	5.57432100	-0.13929700
H	-5.57432100	2.02861100	-0.13929700
H	5.57432100	2.02861100	-0.13929700
H	2.02861100	5.57432100	-0.13929700
H	-4.44355300	6.18418600	-0.26474100
H	-6.18418600	4.44355300	-0.26474100
H	4.44355300	6.18418600	-0.26474100
H	6.18418600	4.44355300	-0.26474100
H	6.18418600	-4.44355300	-0.26474100
H	4.44355300	-6.18418600	-0.26474100
H	-4.44355300	-6.18418600	-0.26474100
H	-6.18418600	-4.44355300	-0.26474100

Optimised coordinates for ground state ZnOBOPC

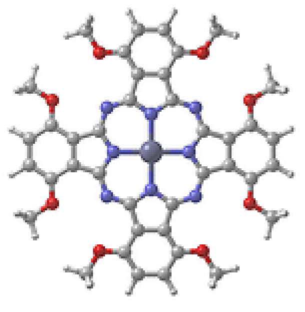

ZnOBOPC, ground state

Experimental compound: zinc octabutyloxy phthylocyanine

Simplified for computation: zinc octamethoxy phthylocyanine

B3LYP-GD3BJ; 6-311G* (C H N O), LanL2TZ (Zn); solvation SMD-DMSO

Multiplicity 1; charge 0

Single-point energy calculated at B3LYP-GD3BJ/6-311+G** (SMD-DMSO): -4363.654403

C	-1.12790300	2.81043400	0.08875800
N	0.00000000	2.04326500	-0.00093800
C	1.12790300	2.81043400	0.08875800
C	0.71026100	4.20714300	0.26664100
C	-0.71026100	4.20714300	0.26664100

N	2.39495000	2.39495000	0.00000000
C	2.81043400	1.12790300	-0.08875800
N	2.04326500	0.00000000	0.00093800
C	2.81043400	-1.12790300	-0.08875800
C	4.20714300	-0.71026100	-0.26664100
C	4.20714300	0.71026100	-0.26664100
N	-2.39495000	2.39495000	0.00000000
C	-4.20714300	0.71026100	-0.26664100
C	-4.20714300	-0.71026100	-0.26664100
C	-2.81043400	-1.12790300	-0.08875800
N	-2.04326500	0.00000000	0.00093800
C	-2.81043400	1.12790300	-0.08875800
N	-2.39495000	-2.39495000	0.00000000
N	0.00000000	-2.04326500	-0.00093800
C	-1.12790300	-2.81043400	0.08875800
C	-0.71026100	-4.20714300	0.26664100
C	0.71026100	-4.20714300	0.26664100
C	1.12790300	-2.81043400	0.08875800
N	2.39495000	-2.39495000	0.00000000
Zn	0.00000000	0.00000000	0.00000000
C	-5.40344300	1.43438200	-0.42796500
C	-6.58814500	0.70172900	-0.57679300
C	-6.58814500	-0.70172900	-0.57679300
C	-5.40344300	-1.43438200	-0.42796500
C	1.43438200	-5.40344300	0.42796500
C	0.70172900	-6.58814500	0.57679300
C	-0.70172900	-6.58814500	0.57679300
C	-1.43438200	-5.40344300	0.42796500
C	5.40344300	1.43438200	-0.42796500
C	6.58814500	0.70172900	-0.57679300
C	6.58814500	-0.70172900	-0.57679300
C	5.40344300	-1.43438200	-0.42796500
C	-1.43438200	5.40344300	0.42796500
C	-0.70172900	6.58814500	0.57679300
C	0.70172900	6.58814500	0.57679300
C	1.43438200	5.40344300	0.42796500
H	7.53526400	1.21172500	-0.70247700
H	7.53526400	-1.21172500	-0.70247700
H	1.21172500	7.53526400	0.70247700

H	-1.21172500	7.53526400	0.70247700
H	-7.53526400	1.21172500	-0.70247700
H	-7.53526400	-1.21172500	-0.70247700
H	-1.21172500	-7.53526400	0.70247700
H	1.21172500	-7.53526400	0.70247700
O	2.78980000	5.33817100	0.43768700
O	-2.78980000	5.33817100	0.43768700
O	5.33817100	2.78980000	-0.43768700
O	5.33817100	-2.78980000	-0.43768700
O	2.78980000	-5.33817100	0.43768700
O	-2.78980000	-5.33817100	0.43768700
O	-5.33817100	-2.78980000	-0.43768700
O	-5.33817100	2.78980000	-0.43768700
C	3.52369700	6.55913700	0.54282900
H	3.31611500	7.07228200	1.48925800
H	3.30220400	7.22931900	-0.29638800
H	4.57583700	6.27197700	0.51069900
C	6.55913700	3.52369700	-0.54282900
H	7.07228200	3.31611500	-1.48925800
H	7.22931900	3.30220400	0.29638800
H	6.27197700	4.57583700	-0.51069900
C	6.55913700	-3.52369700	-0.54282900
H	7.22931900	-3.30220400	0.29638800
H	7.07228200	-3.31611500	-1.48925800
H	6.27197700	-4.57583700	-0.51069900
C	-3.52369700	6.55913700	0.54282900
H	-3.30220400	7.22931900	-0.29638800
H	-3.31611500	7.07228200	1.48925800
H	-4.57583700	6.27197700	0.51069900
C	-6.55913700	3.52369700	-0.54282900
H	-7.22931900	3.30220400	0.29638800
H	-7.07228200	3.31611500	-1.48925800
H	-6.27197700	4.57583700	-0.51069900
C	-6.55913700	-3.52369700	-0.54282900
H	-7.07228200	-3.31611500	-1.48925800
H	-7.22931900	-3.30220400	0.29638800
H	-6.27197700	-4.57583700	-0.51069900
C	-3.52369700	-6.55913700	0.54282900
H	-3.31611500	-7.07228200	1.48925800

H	-3.30220400	-7.22931900	-0.29638800
H	-4.57583700	-6.27197700	0.51069900
C	3.52369700	-6.55913700	0.54282900
H	3.30220400	-7.22931900	-0.29638800
H	3.31611500	-7.07228200	1.48925800
H	4.57583700	-6.27197700	0.51069900

Optimised coordinates for T_1 state ZnOBOPC

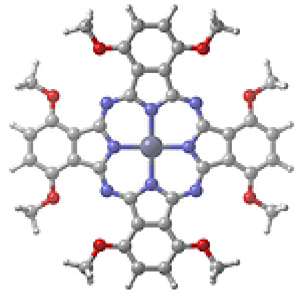

^{3}ZnOBOPC*, T_1 state (UDFT)

Experimental compound: zinc octabutyloxy phthylocyanine

Simplified for computation: zinc octamethoxy phthylocyanine

B3LYP-GD3BJ; 6-311G* (C H N O), LanL2TZ (Zn); solvation SMD-DMSO

Multiplicity 3; charge 0

Single-point energy calculated at B3LYP-GD3BJ/6-311+G** (SMD-DMSO): -4363.622015

C	-1.12617400	2.79976500	-0.01820700
N	-0.00179200	2.03785100	-0.16920700
C	1.12123900	2.80172900	-0.01800700
C	0.70955700	4.16616200	0.24778000
C	-0.71690900	4.16490300	0.24766700
N	2.40762300	2.38526300	-0.11286100
C	2.79668600	1.13280600	-0.18723100
N	2.01899100	0.00179300	-0.12899800
C	2.79865300	-1.12788000	-0.18744600
C	4.20890100	-0.70119500	-0.31398800
C	4.20769300	0.70854000	-0.31381600
N	-2.41178800	2.38104600	-0.11332200
C	-4.20892200	0.70120400	-0.31396500
C	-4.20770300	-0.70853500	-0.31384700
C	-2.79669200	-1.13279500	-0.18727400
N	-2.01901000	-0.00177700	-0.12903100
C	-2.79866500	1.12789400	-0.18748300
N	-2.40762100	-2.38526300	-0.11289000
N	0.00180800	-2.03786400	-0.16919100
C	-1.12122400	-2.80173600	-0.01800400
C	-0.70953900	-4.16617100	0.24778200
C	0.71693000	-4.16489500	0.24774400
C	1.12618800	-2.79976400	-0.01816500
N	2.41179200	-2.38103700	-0.11328000
Zn	-0.00000700	-0.00000900	-0.36753700
C	-5.40258300	1.42681200	-0.40744000
C	-6.59596200	0.69001800	-0.49971600
C	-6.59477700	-0.70142200	-0.49959600

C	-5.40013800	-1.43616500	-0.40719500
C	1.43897600	-5.34838200	0.49882400
C	0.70621300	-6.51515500	0.74020900
C	-0.69453700	-6.51641800	0.74021000
C	-1.42942800	-5.35097500	0.49884000
C	5.40013300	1.43616100	-0.40714900
C	6.59476300	0.70141300	-0.49962100
C	6.59593200	-0.69002800	-0.49983500
C	5.40254700	-1.42681400	-0.40755900
C	-1.43894900	5.34841200	0.49867400
C	-0.70618400	6.51517500	0.74009600
C	0.69456500	6.51641300	0.74019100
C	1.42945000	5.35095700	0.49887000
H	7.54483800	1.21278600	-0.57770400
H	7.54685200	-1.19977800	-0.57811300
H	1.20147400	7.45127600	0.93781600
H	-1.21475300	7.44914700	0.93769100
H	-7.54689100	1.19976400	-0.57791500
H	-7.54485000	-1.21279700	-0.57767600
H	-1.20144100	-7.45128600	0.93781700
H	1.21478300	-7.44911700	0.93784800
O	2.77892600	5.29429200	0.49635900
O	-2.78831600	5.28929700	0.49600400
O	5.33956200	2.78403500	-0.40953600
O	5.34421000	-2.77479400	-0.41041200
O	2.78834500	-5.28923400	0.49623700
O	-2.77890500	-5.29432700	0.49625500
O	-5.33955100	-2.78403900	-0.40965200
O	-5.34428200	2.77479300	-0.41016400
C	3.50716200	6.48555000	0.78396300
H	3.27132300	6.86656600	1.78194700
H	3.31421400	7.26432700	0.04025300
H	4.55740000	6.20237000	0.74492800
C	6.55987500	3.52159600	-0.45075200
H	7.11661400	3.32739400	-1.37212600
H	7.19189900	3.29746600	0.41367000
H	6.26655400	4.56927200	-0.42154300
C	6.56570000	-3.51038900	-0.45185400
H	7.19739900	-3.28553900	0.41261700
H	7.12211300	-3.31508200	-1.37319300
H	6.27400200	-4.55852700	-0.42292900
C	-3.51875200	6.47924500	0.78341300
H	-3.32748100	7.25818100	0.03942400
H	-3.28345700	6.86101900	1.78123700
H	-4.56844300	6.19401900	0.74463200
C	-6.56579800	3.51034900	-0.45145900
H	-7.19744100	3.28538100	0.41302400
H	-7.12225700	3.31512200	-1.37278800
H	-6.27413600	4.55849500	-0.42243300
C	-6.55984700	-3.52162900	-0.45089800
H	-7.11659700	-3.32739400	-1.37225900
H	-7.19187100	-3.29756100	0.41354000
H	-6.26649900	-4.56929800	-0.42174700

C	-3.50714200	-6.48559400	0.78381700
H	-3.27134700	-6.86661100	1.78181100
H	-3.31415300	-7.26436700	0.04011400
H	-4.55738100	-6.20242300	0.74473300
C	3.51880400	-6.47914700	0.78373300
H	3.32761300	-7.25811500	0.03975700
H	3.28345300	-6.86089700	1.78155300
H	4.56848800	-6.19388600	0.74501700

Optimised coordinates for ZnOBOPC anion radical

ZnOBOPC$^{\bullet-}$, anion radical

Experimental compound: zinc octabutyloxy phthylocyanine

Simplified for computation: zinc octamethoxy phthylocyanine

B3LYP-GD3BJ; 6-311G* (C H N O), LanL2TZ (Zn); solvation SMD-DMSO

Multiplicity 2; charge -1

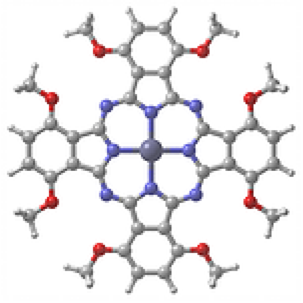

Single-point energy calculated at B3LYP-GD3BJ/6-311+G** (SMD-DMSO): -4363.770113

C	-1.12864600	2.82290100	-0.01255000
N	0.00000000	2.05057600	0.00454000
C	1.12864600	2.82290100	-0.01255000
C	0.71491800	4.21087500	-0.04622200
C	-0.71491800	4.21087500	-0.04622200
N	2.41370100	2.39044300	0.00536500
C	2.81294100	1.13473600	0.01782700
N	2.03906500	0.00000000	0.00234800
C	2.81294100	-1.13473600	0.01782700
C	4.22615600	-0.70641700	0.04847200
C	4.22615600	0.70641700	0.04847200
N	-2.41370100	2.39044300	0.00536500
C	-4.22615600	0.70641700	0.04847200
C	-4.22615600	-0.70641700	0.04847200
C	-2.81294100	-1.13473600	0.01782700
N	-2.03906500	0.00000000	0.00234800
C	-2.81294100	1.13473600	0.01782700
N	-2.41370100	-2.39044300	0.00536500
N	0.00000000	-2.05057600	0.00454000
C	-1.12864600	-2.82290100	-0.01255000
C	-0.71491800	-4.21087500	-0.04622200
C	0.71491800	-4.21087500	-0.04622200
C	1.12864600	-2.82290100	-0.01255000
N	2.41370100	-2.39044300	0.00536500
Zn	0.00000000	0.00000000	-0.00020700
C	-5.43155800	1.42958400	0.07651700
C	-6.62257700	0.69992800	0.10216100
C	-6.62257700	-0.69992800	0.10216100

C	-5.43155800	-1.42958400	0.07651700
C	1.43201400	-5.43127000	-0.07772300
C	0.70665000	-6.61414400	-0.10476900
C	-0.70665000	-6.61414400	-0.10476900
C	-1.43201400	-5.43127000	-0.07772300
C	5.43155800	1.42958400	0.07651700
C	6.62257700	0.69992800	0.10216100
C	6.62257700	-0.69992800	0.10216100
C	5.43155800	-1.42958400	0.07651700
C	-1.43201400	5.43127000	-0.07772300
C	-0.70665000	6.61414400	-0.10476900
C	0.70665000	6.61414400	-0.10476900
C	1.43201400	5.43127000	-0.07772300
H	7.57701900	1.20906100	0.12411700
H	7.57701900	-1.20906100	0.12411700
H	1.21353200	7.56990400	-0.12890100
H	-1.21353200	7.56990400	-0.12890100
H	-7.57701900	1.20906100	0.12411700
H	-7.57701900	-1.20906100	0.12411700
H	-1.21353200	-7.56990400	-0.12890100
H	1.21353200	-7.56990400	-0.12890100
O	2.78986000	5.36746700	-0.08112300
O	-2.78986000	5.36746700	-0.08112300
O	5.37184000	2.78405900	0.07835900
O	5.37184000	-2.78405900	0.07835900
O	2.78986000	-5.36746700	-0.08112300
O	-2.78986000	-5.36746700	-0.08112300
O	-5.37184000	-2.78405900	0.07835900
O	-5.37184000	2.78405900	0.07835900
C	3.51330100	6.58978000	-0.10912700
H	3.29684000	7.16751100	-1.01414900
H	4.56602300	6.31068000	-0.10515200
C	6.59592200	3.50858100	0.10324800
H	7.17388800	3.29374000	1.00795400
H	6.31450800	4.56039000	0.09841600
C	6.59592200	-3.50858100	0.10324800
H	7.17388800	-3.29374000	1.00795400
H	6.31450800	-4.56039000	0.09841600
C	-3.51330100	6.58978000	-0.10912700
H	-3.29684000	7.16751100	-1.01414900
H	-4.56602300	6.31068000	-0.10515200
C	-6.59592200	3.50858100	0.10324800
H	-7.17388800	3.29374000	1.00795400
H	-6.31450800	4.56039000	0.09841600
C	-6.59592200	-3.50858100	0.10324800
H	-7.17388800	-3.29374000	1.00795400
H	-6.31450800	-4.56039000	0.09841600
C	-3.51330100	-6.58978000	-0.10912700
H	-3.29684000	-7.16751100	-1.01414900
H	-4.56602300	-6.31068000	-0.10515200
C	3.51330100	-6.58978000	-0.10912700
H	3.29684000	-7.16751100	-1.01414900
H	4.56602300	-6.31068000	-0.10515200

H	3.30018000	-7.20611500	0.77086900
H	-3.30018000	-7.20611500	0.77086900
H	-7.20924100	-3.29485400	-0.77815800
H	-7.20924100	3.29485400	-0.77815800
H	3.30018000	7.20611500	0.77086900
H	-3.30018000	7.20611500	0.77086900
H	7.20924100	-3.29485400	-0.77815800
H	7.20924100	3.29485400	-0.77815800

Optimised coordinates for ZnOBOPC cation radical

ZnOBOPC•+, cation radical

Experimental compound: zinc octabutyloxy phthylocyanine

Simplified for computation: zinc octamethoxy phthylocyanine

B3LYP-GD3BJ; 6-311G* (C H N O), LanL2TZ (Zn); solvation SMD-DMSO

Multiplicity 2; charge 1

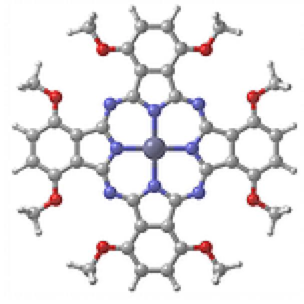

Single-point energy calculated at B3LYP-GD3BJ/6-311+G** (SMD-DMSO): -4363.491979

C	-1.12547700	2.80500300	0.04388100
N	0.00000000	2.03765300	-0.02288400
C	1.12547700	2.80500300	0.04388100
C	0.70845000	4.20636000	0.16332100
C	-0.70845000	4.20636000	0.16332100
N	2.39145300	2.39145300	0.00000000
C	2.80500300	1.12547700	-0.04388100
N	2.03765300	0.00000000	0.02288400
C	2.80500300	-1.12547700	-0.04388100
C	4.20636000	-0.70845000	-0.16332100
C	4.20636000	0.70845000	-0.16332100
N	-2.39145300	2.39145300	0.00000000
C	-4.20636000	0.70845000	-0.16332100
C	-4.20636000	-0.70845000	-0.16332100
C	-2.80500300	-1.12547700	-0.04388100
N	-2.03765300	0.00000000	0.02288400
C	-2.80500300	1.12547700	-0.04388100
N	-2.39145300	-2.39145300	0.00000000
N	0.00000000	-2.03765300	-0.02288400
C	-1.12547700	-2.80500300	0.04388100

C	-0.70845000	-4.20636000	0.16332100
C	0.70845000	-4.20636000	0.16332100
C	1.12547700	-2.80500300	0.04388100
N	2.39145300	-2.39145300	0.00000000
Zn	0.00000000	0.00000000	0.00000000
C	-5.39928400	1.43431300	-0.26889000
C	-6.59356300	0.69416400	-0.36897300
C	-6.59356300	-0.69416400	-0.36897300
C	-5.39928400	-1.43431300	-0.26889000
C	1.43431300	-5.39928400	0.26889000
C	0.69416400	-6.59356300	0.36897300
C	-0.69416400	-6.59356300	0.36897300
C	-1.43431300	-5.39928400	0.26889000
C	5.39928400	1.43431300	-0.26889000
C	6.59356300	0.69416400	-0.36897300
C	6.59356300	-0.69416400	-0.36897300
C	5.39928400	-1.43431300	-0.26889000
C	-1.43431300	5.39928400	0.26889000
C	-0.69416400	6.59356300	0.36897300
C	0.69416400	6.59356300	0.36897300
C	1.43431300	5.39928400	0.26889000
H	7.54337100	1.20450400	-0.45335600
H	7.54337100	-1.20450400	-0.45335600
H	1.20450400	7.54337100	0.45335600
H	-1.20450400	7.54337100	0.45335600
H	-7.54337100	1.20450400	-0.45335600
H	-7.54337100	-1.20450400	-0.45335600
H	-1.20450400	-7.54337100	0.45335600
H	1.20450400	-7.54337100	0.45335600
O	2.77651900	5.34209200	0.27319800
O	-2.77651900	5.34209200	0.27319800
O	5.34209200	2.77651900	-0.27319800
O	5.34209200	-2.77651900	-0.27319800
O	2.77651900	-5.34209200	0.27319800
O	-2.77651900	-5.34209200	0.27319800
O	-5.34209200	-2.77651900	-0.27319800
O	-5.34209200	2.77651900	-0.27319800
C	3.51652500	6.56315400	0.35321800
H	3.30859400	7.09306800	1.28641100

H	3.30073100	7.21326200	-0.49880100
H	4.56323300	6.26746200	0.32920800
C	6.56315400	3.51652500	-0.35321800
H	7.09306800	3.30859400	-1.28641100
H	7.21326200	3.30073100	0.49880100
H	6.26746200	4.56323300	-0.32920800
C	6.56315400	-3.51652500	-0.35321800
H	7.21326200	-3.30073100	0.49880100
H	7.09306800	-3.30859400	-1.28641100
H	6.26746200	-4.56323300	-0.32920800
C	-3.51652500	6.56315400	0.35321800
H	-3.30073100	7.21326200	-0.49880100
H	-3.30859400	7.09306800	1.28641100
H	-4.56323300	6.26746200	0.32920800
C	-6.56315400	3.51652500	-0.35321800
H	-7.21326200	3.30073100	0.49880100
H	-7.09306800	3.30859400	-1.28641100
H	-6.26746200	4.56323300	-0.32920800
C	-6.56315400	-3.51652500	-0.35321800
H	-7.09306800	-3.30859400	-1.28641100
H	-7.21326200	-3.30073100	0.49880100
H	-6.26746200	-4.56323300	-0.32920800
C	-3.51652500	-6.56315400	0.35321800
H	-3.30859400	-7.09306800	1.28641100
H	-3.30073100	-7.21326200	-0.49880100
H	-4.56323300	-6.26746200	0.32920800
C	3.51652500	-6.56315400	0.35321800
H	3.30073100	-7.21326200	-0.49880100
H	3.30859400	-7.09306800	1.28641100
H	4.56323300	-6.26746200	0.32920800

Optimised coordinates for ground state ZnTtBNPC	
**ZnTtBNPC, ground state**	
Experimental compound: zinc tetra(tert-butyl) naphthylocyanine	
Simplified for computation: zinc naphthylocyanine	

B3LYP-GD3BJ; 6-311G* (C H N), LanL2TZ (Zn); solvation SMD-DMSO	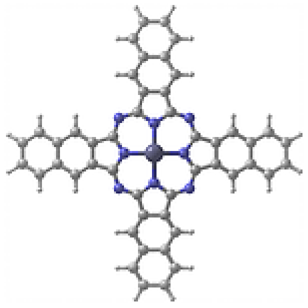
Multiplicity 1; charge 0	
Single-point energy calculated at B3LYP-GD3BJ/6-311+G** (SMD-DMSO): -4061.963942	

C	1.12207700	2.79302100	0.17278400
N	0.00000000	2.02036100	0.25982100
C	-1.12207700	2.79302100	0.17278400
C	-0.71452400	4.19463500	0.05146800
C	0.71452400	4.19463500	0.05146800
N	-2.38950600	2.38950500	0.15461300
C	-2.79302300	1.12207700	0.17280100
N	-2.02036200	0.00000000	0.25982600
C	-2.79302300	-1.12207700	0.17280100
C	-4.19464100	-0.71452400	0.05152600
C	-4.19464100	0.71452400	0.05152600
N	2.38950600	2.38950500	0.15461300
C	4.19464100	0.71452400	0.05152600
C	4.19464100	-0.71452400	0.05152600
C	2.79302300	-1.12207700	0.17280100
N	2.02036200	0.00000000	0.25982600
C	2.79302300	1.12207700	0.17280100
N	2.38950600	-2.38950500	0.15461300
N	0.00000000	-2.02036100	0.25982100
C	1.12207700	-2.79302100	0.17278400
C	0.71452400	-4.19463500	0.05146800
C	-0.71452400	-4.19463500	0.05146800
C	-1.12207700	-2.79302100	0.17278400
N	-2.38950600	-2.38950500	0.15461300
Zn	0.00000000	0.00000000	0.77752800
C	5.36349200	1.42745400	-0.05461600
C	6.58861000	0.72073000	-0.15637700
C	6.58861000	-0.72073000	-0.15637700
C	5.36349200	-1.42745400	-0.05461600
C	-1.42745400	-5.36348300	-0.05470800
C	-0.72073000	-6.58859800	-0.15650100
C	0.72073000	-6.58859800	-0.15650100
C	1.42745400	-5.36348300	-0.05470800
C	-5.36349200	1.42745400	-0.05461600
C	-6.58861000	0.72073000	-0.15637700
C	-6.58861000	-0.72073000	-0.15637700
C	-5.36349200	-1.42745400	-0.05461600
C	1.42745400	5.36348300	-0.05470800
C	0.72073000	6.58859800	-0.15650100
C	-0.72073000	6.58859800	-0.15650100
C	-1.42745400	5.36348300	-0.05470800
C	1.40241200	-7.83041700	-0.25896100

C	0.70807400	-9.01150700	-0.35678400
C	-0.70807400	-9.01150700	-0.35678400
C	-1.40241200	-7.83041700	-0.25896100
C	7.83043100	-1.40241200	-0.25880300
C	9.01152400	-0.70807400	-0.35659300
C	9.01152400	0.70807400	-0.35659300
C	7.83043100	1.40241200	-0.25880300
C	-1.40241200	7.83041700	-0.25896100
C	-0.70807400	9.01150700	-0.35678400
C	0.70807400	9.01150700	-0.35678400
C	1.40241200	7.83041700	-0.25896100
C	-7.83043100	-1.40241200	-0.25880300
C	-9.01152400	-0.70807400	-0.35659300
C	-9.01152400	0.70807400	-0.35659300
C	-7.83043100	1.40241200	-0.25880300
H	5.36498500	2.51242100	-0.05821100
H	5.36498500	-2.51242100	-0.05821100
H	-2.51242100	-5.36497500	-0.05830300
H	2.51242100	-5.36497500	-0.05830300
H	-5.36498500	2.51242100	-0.05821100
H	-5.36498500	-2.51242100	-0.05821100
H	2.51242100	5.36497500	-0.05830300
H	-2.51242100	5.36497500	-0.05830300
H	2.48784300	-7.82854100	-0.25881400
H	1.24326200	-9.95178800	-0.43508800
H	-1.24326200	-9.95178800	-0.43508800
H	-2.48784300	-7.82854100	-0.25881400
H	7.82855600	-2.48784300	-0.25865500
H	9.95180800	-1.24326200	-0.43487000
H	9.95180800	1.24326200	-0.43487000
H	7.82855600	2.48784300	-0.25865500
H	-2.48784300	7.82854100	-0.25881400
H	-1.24326200	9.95178800	-0.43508800
H	1.24326200	9.95178800	-0.43508800
H	2.48784300	7.82854100	-0.25881400
H	-7.82855600	-2.48784300	-0.25865500
H	-9.95180800	-1.24326200	-0.43487000
H	-9.95180800	1.24326200	-0.43487000
H	-7.82855600	2.48784300	-0.25865500

Optimised coordinates for T_1 state ZnTtBNPC	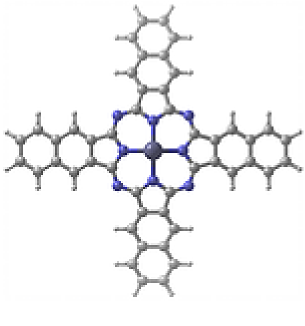
**^{3}ZnTtBNPC*, T_1 state (UDFT)**	
Experimental compound: zinc tetra(tert-butyl) naphthylocyanine	
Simplified for computation: zinc naphthylocyanine	
B3LYP-GD3BJ; 6-311G* (C H N), LanL2TZ (Zn); solvation SMD-DMSO	

Multiplicity 3; charge 0				
Single-point energy calculated at B3LYP-GD3BJ/6-311+G** (SMD-DMSO): -4061.931343				
C	1.12126300	2.79013600	0.18131900	
N	0.00000000	2.02392100	0.32249500	
C	-1.12126300	2.79013600	0.18131900	
C	-0.71770500	4.16623200	-0.04567000	
C	0.71770500	4.16623200	-0.04567000	
N	-2.41272100	2.38359700	0.20859400	
C	-2.79467900	1.12750700	0.26412100	
N	-2.01051700	0.00000000	0.32639400	
C	-2.79467900	-1.12750700	0.26412100	
C	-4.20847900	-0.71227800	0.20924000	
C	-4.20847900	0.71227800	0.20924000	
N	2.41272100	2.38359700	0.20859400	
C	4.20847900	0.71227800	0.20924000	
C	4.20847900	-0.71227800	0.20924000	
C	2.79467900	-1.12750700	0.26412100	
N	2.01051700	0.00000000	0.32639400	
C	2.79467900	1.12750700	0.26412100	
N	2.41272100	-2.38359700	0.20859400	
N	0.00000000	-2.02392100	0.32249500	
C	1.12126300	-2.79013600	0.18131900	
C	0.71770500	-4.16623200	-0.04567000	
C	-0.71770500	-4.16623200	-0.04567000	
C	-1.12126300	-2.79013600	0.18131900	
N	-2.41272100	-2.38359700	0.20859400	
Zn	0.00000000	0.00000000	0.84497200	
C	5.37384000	1.42627700	0.15326700	
C	6.60943700	0.71888300	0.10387300	
C	6.60943700	-0.71888300	0.10387300	
C	5.37384000	-1.42627700	0.15326700	
C	-1.42832400	-5.32591300	-0.25461800	
C	-0.72145600	-6.53579300	-0.46639700	
C	0.72145600	-6.53579300	-0.46639700	
C	1.42832400	-5.32591300	-0.25461800	
C	-5.37384000	1.42627700	0.15326700	
C	-6.60943700	0.71888300	0.10387300	
C	-6.60943700	-0.71888300	0.10387300	
C	-5.37384000	-1.42627700	0.15326700	
C	1.42832400	5.32591300	-0.25461800	
C	0.72145600	6.53579300	-0.46639700	
C	-0.72145600	6.53579300	-0.46639700	
C	-1.42832400	5.32591300	-0.25461800	
C	1.40232500	-7.76253200	-0.68470400	
C	0.70802000	-8.93010200	-0.89221600	
C	-0.70802000	-8.93010200	-0.89221600	
C	-1.40232500	-7.76253200	-0.68470400	
C	7.84909600	-1.40134500	0.05364000	
C	9.03666300	-0.70586500	0.00573600	
C	9.03666300	0.70586500	0.00573600	
C	7.84909600	1.40134500	0.05364000	

C	-1.40232500	7.76253200	-0.68470400
C	-0.70802000	8.93010200	-0.89221600
C	0.70802000	8.93010200	-0.89221600
C	1.40232500	7.76253200	-0.68470400
C	-7.84909600	-1.40134500	0.05364000
C	-9.03666300	-0.70586500	0.00573600
C	-9.03666300	0.70586500	0.00573600
C	-7.84909600	1.40134500	0.05364000
H	5.37389600	2.51115900	0.14823600
H	5.37389600	-2.51115900	0.14823600
H	-2.51335900	-5.32842800	-0.26113000
H	2.51335900	-5.32842800	-0.26113000
H	-5.37389600	2.51115900	0.14823600
H	-5.37389600	-2.51115900	0.14823600
H	2.51335900	5.32842800	-0.26113000
H	-2.51335900	5.32842800	-0.26113000
H	2.48780500	-7.76097600	-0.68427700
H	1.24352100	-9.85890100	-1.05738700
H	-1.24352100	-9.85890100	-1.05738700
H	-2.48780500	-7.76097600	-0.68427700
H	7.84735700	-2.48661500	0.05329100
H	9.97847500	-1.24246900	-0.03283700
H	9.97847500	1.24246900	-0.03283700
H	7.84735700	2.48661500	0.05329100
H	-2.48780500	7.76097600	-0.68427700
H	-1.24352100	9.85890100	-1.05738700
H	1.24352100	9.85890100	-1.05738700
H	2.48780500	7.76097600	-0.68427700
H	-7.84735700	-2.48661500	0.05329100
H	-9.97847500	-1.24246900	-0.03283700
H	-9.97847500	1.24246900	-0.03283700
H	-7.84735700	2.48661500	0.05329100

Optimised coordinates for ZnTtBNPC anion radical

ZnTtBNPC•-, anion radical

Experimental compound: zinc tetra(tert-butyl) naphthylocyanine

Simplified for computation: zinc naphthylocyanine

B3LYP-GD3BJ; 6-311G* (C H N), LanL2TZ (Zn); solvation SMD-DMSO

Multiplicity 2; charge -1

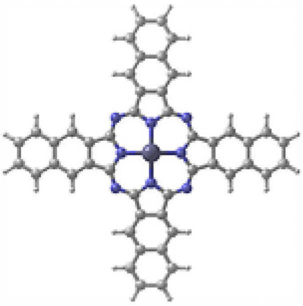

Single-point energy calculated at B3LYP-GD3BJ/6-311+G** (SMD-DMSO): -4062.082644

C	1.12425200	2.80281100	0.12188100
N	0.00000000	2.02928800	0.19801400
C	-1.12425200	2.80281100	0.12188100
C	-0.72003700	4.18840600	0.00601600

C	0.72003700	4.18840600	0.00601600
N	-2.41141800	2.38811400	0.11590200
C	-2.79824600	1.12984700	0.13625200
N	-2.01650600	0.00000000	0.19551300
C	-2.79824600	-1.12984700	0.13625200
C	-4.20685000	-0.71399200	0.06082400
C	-4.20685000	0.71399200	0.06082400
N	2.41141800	2.38811400	0.11590200
C	4.20685000	0.71399200	0.06082400
C	4.20685000	-0.71399200	0.06082400
C	2.79824600	-1.12984700	0.13625200
N	2.01650600	0.00000000	0.19551300
C	2.79824600	1.12984700	0.13625200
N	2.41141800	-2.38811400	0.11590200
N	0.00000000	-2.02928800	0.19801400
C	1.12425200	-2.80281100	0.12188100
C	0.72003700	-4.18840600	0.00601600
C	-0.72003700	-4.18840600	0.00601600
C	-1.12425200	-2.80281100	0.12188100
N	-2.41141800	-2.38811400	0.11590200
Zn	0.00000000	0.00000000	0.69054700
C	5.37873100	1.42505200	-0.00591000
C	6.60971700	0.72026100	-0.06803500
C	6.60971700	-0.72026100	-0.06803500
C	5.37873100	-1.42505200	-0.00591000
C	-1.42726900	-5.37137200	-0.09586100
C	-0.72363700	-6.59035500	-0.19270200
C	0.72363700	-6.59035500	-0.19270200
C	1.42726900	-5.37137200	-0.09586100
C	-5.37873100	1.42505200	-0.00591000
C	-6.60971700	0.72026100	-0.06803500
C	-6.60971700	-0.72026100	-0.06803500
C	-5.37873100	-1.42505200	-0.00591000
C	1.42726900	5.37137200	-0.09586100
C	0.72363700	6.59035500	-0.19270200
C	-0.72363700	6.59035500	-0.19270200
C	-1.42726900	5.37137200	-0.09586100
C	1.40264800	-7.83938100	-0.29065800
C	0.71063400	-9.01916900	-0.38217300
C	-0.71063400	-9.01916900	-0.38217300
C	-1.40264800	-7.83938100	-0.29065800
C	7.85397700	-1.40130600	-0.12910400
C	9.03935200	-0.70767100	-0.18595600
C	9.03935200	0.70767100	-0.18595600
C	7.85397700	1.40130600	-0.12910400
C	-1.40264800	7.83938100	-0.29065800
C	-0.71063400	9.01916900	-0.38217300
C	0.71063400	9.01916900	-0.38217300
C	1.40264800	7.83938100	-0.29065800
C	-7.85397700	-1.40130600	-0.12910400
C	-9.03935200	-0.70767100	-0.18595600
C	-9.03935200	0.70767100	-0.18595600
C	-7.85397700	1.40130600	-0.12910400

H	5.37962900	2.51041500	-0.00877000
H	5.37962900	-2.51041500	-0.00877000
H	-2.51298500	-5.37586600	-0.09899600
H	2.51298500	-5.37586600	-0.09899600
H	-5.37962900	2.51041500	-0.00877000
H	-5.37962900	-2.51041500	-0.00877000
H	2.51298500	5.37586600	-0.09899600
H	-2.51298500	5.37586600	-0.09899600
H	2.48864700	-7.83807900	-0.29070500
H	1.24557400	-9.96032500	-0.45581400
H	-1.24557400	-9.96032500	-0.45581400
H	-2.48864700	-7.83807900	-0.29070500
H	7.85209100	-2.48696200	-0.12920000
H	9.98134300	-1.24391700	-0.23171000
H	9.98134300	1.24391700	-0.23171000
H	7.85209100	2.48696200	-0.12920000
H	-2.48864700	7.83807900	-0.29070500
H	-1.24557400	9.96032500	-0.45581400
H	1.24557400	9.96032500	-0.45581400
H	2.48864700	7.83807900	-0.29070500
H	-7.85209100	-2.48696200	-0.12920000
H	-9.98134300	-1.24391700	-0.23171000
H	-9.98134300	1.24391700	-0.23171000
H	-7.85209100	2.48696200	-0.12920000

Optimised coordinates for ZnTtBNPC cation radical

ZnTtBNPC$^{•+}$, cation radical

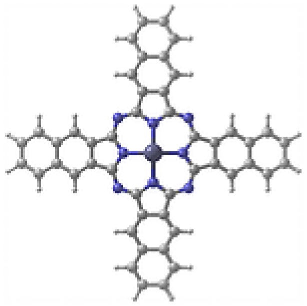

Experimental compound: zinc tetra(tert-butyl) naphthylocyanine

Simplified for computation: zinc naphthylocyanine

B3LYP-GD3BJ; 6-311G* (C H N), LanL2TZ (Zn); solvation SMD-DMSO

Multiplicity 2; charge 1

Single-point energy calculated at B3LYP-GD3BJ/6-311+G** (SMD-DMSO): -4061.797109

C	1.11993400	2.78769300	0.20314900
N	0.00000000	2.01419200	0.30352500
C	-1.11993400	2.78769300	0.20314900
C	-0.71350500	4.18903300	0.06593600
C	0.71350500	4.18903300	0.06593600
N	-2.38847500	2.38847400	0.18442300
C	-2.78769600	1.11993400	0.20317400
N	-2.01419200	0.00000000	0.30352800
C	-2.78769600	-1.11993400	0.20317400
C	-4.18904100	-0.71350500	0.06600600
C	-4.18904100	0.71350500	0.06600600
N	2.38847500	2.38847400	0.18442300

C	4.18904100	0.71350500	0.06600600
C	4.18904100	-0.71350500	0.06600600
C	2.78769600	-1.11993400	0.20317400
N	2.01419200	0.00000000	0.30352800
C	2.78769600	1.11993400	0.20317400
N	2.38847500	-2.38847400	0.18442300
N	0.00000000	-2.01419200	0.30352500
C	1.11993400	-2.78769300	0.20314900
C	0.71350500	-4.18903300	0.06593600
C	-0.71350500	-4.18903300	0.06593600
C	-1.11993400	-2.78769300	0.20314900
N	-2.38847500	-2.38847400	0.18442300
Zn	0.00000000	0.00000000	0.83945000
C	5.35001200	1.42892700	-0.05638200
C	6.57791900	0.71949200	-0.17494200
C	6.57791900	-0.71949200	-0.17494200
C	5.35001200	-1.42892700	-0.05638200
C	-1.42892700	-5.35000000	-0.05649400
C	-0.71949200	-6.57790200	-0.17509700
C	0.71949200	-6.57790200	-0.17509700
C	1.42892700	-5.35000000	-0.05649400
C	-5.35001200	1.42892700	-0.05638200
C	-6.57791900	0.71949200	-0.17494200
C	-6.57791900	-0.71949200	-0.17494200
C	-5.35001200	-1.42892700	-0.05638200
C	1.42892700	5.35000000	-0.05649400
C	0.71949200	6.57790200	-0.17509700
C	-0.71949200	6.57790200	-0.17509700
C	-1.42892700	5.35000000	-0.05649400
C	1.40225600	-7.81220400	-0.29471300
C	0.70576700	-8.99456600	-0.40960300
C	-0.70576700	-8.99456600	-0.40960300
C	-1.40225600	-7.81220400	-0.29471300
C	7.81222500	-1.40225600	-0.29451300
C	8.99459100	-0.70576700	-0.40936000
C	8.99459100	0.70576700	-0.40936000
C	7.81222500	1.40225600	-0.29451300
C	-1.40225600	7.81220400	-0.29471300
C	-0.70576700	8.99456600	-0.40960300
C	0.70576700	8.99456600	-0.40960300
C	1.40225600	7.81220400	-0.29471300
C	-7.81222500	-1.40225600	-0.29451300
C	-8.99459100	-0.70576700	-0.40936000
C	-8.99459100	0.70576700	-0.40936000
C	-7.81222500	1.40225600	-0.29451300
H	5.35035800	2.51347600	-0.06164300
H	5.35035800	-2.51347600	-0.06164300
H	-2.51347600	-5.35034600	-0.06175500
H	2.51347600	-5.35034600	-0.06175500
H	-5.35035800	2.51347600	-0.06164300
H	-5.35035800	-2.51347600	-0.06164300
H	2.51347600	5.35034600	-0.06175500
H	-2.51347600	5.35034600	-0.06175500

H	2.48727300	-7.81043600	-0.29454400
H	1.24178000	-9.93282300	-0.50123800
H	-1.24178000	-9.93282300	-0.50123800
H	-2.48727300	-7.81043600	-0.29454400
H	7.81045700	-2.48727300	-0.29434400
H	9.93285200	-1.24178000	-0.50096100
H	9.93285200	1.24178000	-0.50096100
H	7.81045700	2.48727300	-0.29434400
H	-2.48727300	7.81043600	-0.29454400
H	-1.24178000	9.93282300	-0.50123800
H	1.24178000	9.93282300	-0.50123800
H	2.48727300	7.81043600	-0.29454400
H	-7.81045700	-2.48727300	-0.29434400
H	-9.93285200	-1.24178000	-0.50096100
H	-9.93285200	1.24178000	-0.50096100
H	-7.81045700	2.48727300	-0.29434400

Optimised coordinates for ground state DTPA

DTPA, ground state

Experimental compound: 2-(dodecylthiocarbonothioylthio)propionic acid

Simplified for computation: 2-(butylthiocarbonothioylthio)propionic acid

B3LYP-GD3BJ; 6-311G* (C H O S); solvation SMD-DMSO

Multiplicity 1; charge 0

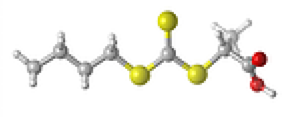

Single-point energy calculated at B3LYP-GD3BJ/6-311+G** (SMD-DMSO): -1658.608356

C	4.06665900	-0.32072000	0.54316800
C	2.91323700	0.42152100	-0.10374400
O	4.53322300	-0.05402200	1.62092100
O	4.52455900	-1.31588000	-0.23835800
C	3.28329300	1.05564100	-1.44046700
S	1.53022300	-0.79634100	-0.26738100
C	0.07787500	0.19785900	-0.04165500
S	-1.32122100	-0.85098900	-0.20667700
S	0.03673900	1.81822400	0.26679000
C	-2.75124800	0.26831800	0.04303400
C	-4.03659800	-0.55084400	-0.02721900
C	-5.26488900	0.33937300	0.18395900
C	-6.56963300	-0.45283400	0.13727800
H	2.57601400	1.17181900	0.60760900
H	5.25578000	-1.76033800	0.22547100
H	2.43743600	1.61926900	-1.83541700
H	4.11668900	1.74980000	-1.29866300
H	3.57777200	0.30429000	-2.17328500
H	-2.63632100	0.74842700	1.01457300
H	-2.71825900	1.03297300	-0.73324800

H	-4.11174200	-1.05141000	-0.99848300
H	-4.02104600	-1.33541900	0.73682700
H	-5.17806900	0.85076600	1.14935500
H	-5.27981300	1.12292700	-0.58205000
H	-7.43518800	0.19743600	0.29288500
H	-6.69811500	-0.95073700	-0.82908600
H	-6.59143500	-1.22476800	0.91316000

Optimised coordinates for DTPA anion radical

DTPA•-, anion radical

Experimental compound: 2-(dodecylthiocarbonothioylthio)propionic acid

Simplified for computation: 2-(butylthiocarbonothioylthio)propionic acid

B3LYP-GD3BJ; 6-311G* (C H O S); solvation SMD-DMSO

Multiplicity 2; charge -1

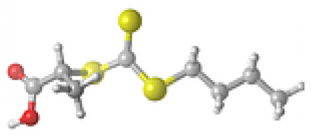

Single-point energy calculated at B3LYP-GD3BJ/6-311+G** (SMD-DMSO): -1658.711482

C	-4.10424400	-0.46722100	-0.12017600
C	-2.84618400	0.11669400	0.45252600
O	-4.92263300	0.15317000	-0.76287700
O	-4.23067300	-1.79236900	0.11135000
C	-2.28589800	-0.57939700	1.67756800
S	-1.59636400	0.10624900	-0.99301700
C	-0.12563900	0.70816200	-0.24985100
S	1.07335200	-0.58663700	0.06734900
S	0.02175100	2.31061000	0.36249200
C	2.67941500	0.28860600	-0.10683900
C	3.83561900	-0.70122100	-0.01500000
C	5.19463100	-0.00506700	-0.13327800
C	6.36497400	-0.98332600	-0.05494600
H	-3.03868500	1.17000200	0.65107400
H	-5.04811800	-2.08569900	-0.32516700
H	-1.37709100	-0.07173100	2.00336100
H	-3.00668100	-0.54079600	2.50059100
H	-2.04648000	-1.62380300	1.47696900
H	2.67578000	0.80080000	-1.07171100
H	2.74648600	1.04312900	0.67712900
H	3.78738500	-1.24053700	0.93853000
H	3.74384000	-1.45506900	-0.80530400
H	5.23750600	0.54449200	-1.08104900
H	5.28826600	0.74470300	0.66112500
H	7.32534300	-0.46695500	-0.14555900
H	6.36873500	-1.52274300	0.89796000
H	6.31430100	-1.72914300	-0.85494600

Optimised coordinates for ground state TEA	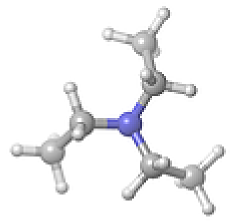
**TEA, ground state**	
Experimental compound: triethylamine	
Simplified for computation: (no need to simplify)	
B3LYP-GD3BJ; 6-311G* (C H N); solvation SMD-DMSO	
Multiplicity 1; charge 0	
Single-point energy calculated at B3LYP-GD3BJ/6-311+G** (SMD-DMSO): -292.535922	

N	-0.00007500	-0.00028900	0.01528000
C	-1.40293300	0.02132200	0.44826700
C	0.68333500	-1.22560800	0.44817300
C	0.71910200	1.20406300	0.44965500
C	-2.30767800	-0.82634800	-0.43926500
C	1.87024000	-1.58355300	-0.43940000
C	0.43832700	2.41020300	-0.43992000
H	-1.75426700	1.05448100	0.41306100
H	-1.49325200	-0.29896700	1.50156200
H	-0.03451000	-2.04737500	0.41347700
H	1.00648400	-1.14282300	1.50116400
H	1.78973200	0.99265800	0.41899400
H	0.48225000	1.44325800	1.50163300
H	-3.34547900	-0.76810600	-0.09670200
H	-2.02117000	-1.88138400	-0.43617400
H	-2.26840100	-0.47166200	-1.47319800
H	2.33766600	-2.51266200	-0.09878300
H	2.64090600	-0.80819500	-0.43345500
H	1.54363600	-1.72381700	-1.47381200
H	1.00447100	3.28165000	-0.09667800
H	-0.61950900	2.68626700	-0.44042300
H	0.72961500	2.19822600	-1.47268600

Optimised coordinates for TEA cation radical	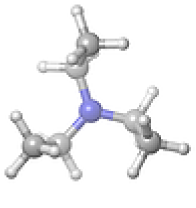
**TEA$^{•+}$, cation radical**	
Experimental compound: triethylamine	
Simplified for computation: (no need to simplify)	
B3LYP-GD3BJ; 6-311G* (C H N); solvation SMD-DMSO	
Multiplicity 2; charge 1	
Single-point energy calculated at B3LYP-GD3BJ/6-311+G** (SMD-DMSO): -292.356371	

N	0.000158	-0.002085	-0.615506
C	-0.983122	-1.068480	-0.605883
C	-0.430996	1.382724	-0.609931
C	1.415140	-0.319906	-0.608205
C	-1.777206	-1.102159	0.706948
C	-0.072280	2.087734	0.705396
C	1.848170	-0.978828	0.708592
H	-0.464305	-2.009334	-0.777237
H	-1.661004	-0.877292	-1.444255
H	-1.503932	1.404990	-0.789252
H	0.081097	1.873978	-1.443724
H	1.968878	0.598759	-0.790580
H	1.585229	-1.010882	-1.440419
H	-2.491798	-1.925104	0.646048
H	-2.332842	-0.177379	0.866135
H	-1.116344	-1.274051	1.557066
H	-0.420857	3.120054	0.638710
H	1.004725	2.100220	0.875690
H	-0.562817	1.606195	1.551784
H	2.917981	-1.186028	0.644455
H	1.326662	-1.921537	0.877993
H	1.669991	-0.314500	1.554627

Optimised coordinates for ground state molecular oxygen

O_2, ground state

Experimental compound: molecular oxygen

Simplified for computation: (no need to simplify)

B3LYP-GD3BJ; 6-311G* (O); solvation SMD-DMSO

Multiplicity 3; charge 0

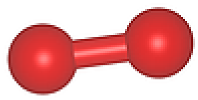

Single-point energy calculated at B3LYP-GD3BJ/6-311+G** (SMD-DMSO): -150.382828

O	0.00000000	0.00000000	0.60259200
O	0.00000000	0.00000000	-0.60259200

Optimised coordinates for singlet oxygen

$^1O_2$, singlet oxygen

Experimental compound: singlet oxygen

Simplified for computation: (no need to simplify)

B3LYP-GD3BJ; 6-311G* (O); solvation SMD-DMSO

Multiplicity 1; charge 0	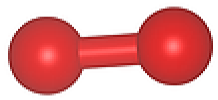
Single-point energy calculated at B3LYP-GD3BJ/6-311+G** (SMD-DMSO): -150.321734	
O 0.00000000 0.00000000 0.60304200<br>O 0.00000000 0.00000000 -0.60304200	

Optimised coordinates for superoxide	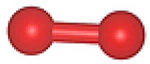
**$O_2^{•-}$, superoxide**	
Experimental compound: superoxide	
Simplified for computation: (no need to simplify)	
B3LYP-GD3BJ; 6-311G* (O); solvation SMD-DMSO	
Multiplicity 2; charge -1	
Single-point energy calculated at B3LYP-GD3BJ/6-311+G** (SMD-DMSO): -150.503105	
O 0.00000000 0.00000000 0.67366800<br>O 0.00000000 0.00000000 -0.67366800	

13.2 Molecular orbital transition and wave function analysis

The check point files of the ground state PCs (at relaxed structure) containing wave function information were obtained from DFT calculations in the previous subsection and converted to .fchk files by Gaussian09 D01 software for wave function analyses. Highest occupied molecular orbitals (HOMO), lowest unoccupied molecular orbitals (LUMO), HOMO+1 and LUMO-1 were visualised by the Multiwfn software package with main function 1 (isovalue = 0.3).[2] π-electron localized orbital locator (LOL) analyses with visualised isosurfaces were conducted by the orbital localisation function of the Multiwfn software package (LOL, medium quality grids, isovalue = 0.2).[2]

Time-dependent DFT (TD-DFT) calculations were performed using the optimised geometries of the ground state PC molecules obtained in the previous subsection, by Gaussian09 D01 software with the keyword td(nstates=10), B3LYP-GD3BJ as the functional, 6-311G* basis set for C H N O, LanL2TZ pseudopotential basis set for Zn and solvation model SMD-DMSO. TD-DFT calculations were used to reveal the percentage contribution of HOMO→LUMO or HOMO-1→LUMO/LUMO+1 transitions to S_1 vertical excitation for each PC.

13.3 Calculating excited state energies with ZnOEP as an example

To present the Jablonski diagram as **Supplementary Figure 33** in the main text, we select ZnOEP as an example and calculated its relevant excited state energies. The TD-DFT approach by Gaussian09 D01 software was used for geometry optimisation and energy calculations of the related ZnOEP excited states. Because the ground state (S_0) and the lowest triplet excited state (T_1) of ZnOEP have been calculated by DFT and unrestricted DFT (UDFT) respectively (presented as above), here we only used TD-DFT to calculate the lowest singlet excited state (S_1) and the second lowest triplet excited state (T_2). It should be noted that because of the intrinsic symmetry of the metalloporphyrin ZnOEP, there are as expected two identical T_1 and two identical T_2 listed in the computation output; therefore, setting root = 1 and root = 2 in the keyword td both corresponds to T_1 while root = 3 (or 4) corresponds to T_2. Details are listed as below.

Related molecular coordinates:

S_0 energy at B3LYP-GD3BJ; 6-311G* (C H N), LanL2TZ (Zn); solvation SMD-DMSO: -1369.048693

T_1 energy at B3LYP-GD3BJ; 6-311G* (C H N), LanL2TZ (Zn); solvation SMD-DMSO: -1368.983407

Optimised coordinates for S_1 state ZnOEP	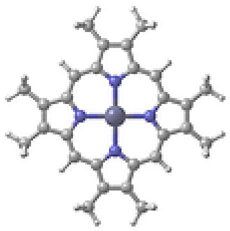
**S_1 state ZnOEP (TD-DFT)**	
Experimental compound: zinc octaethyl porphyrin	
Simplified for computation: zinc octamethyl porphyrin	
B3LYP-GD3BJ; 6-311G* (C H N), LanL2TZ (Zn); solvation SMD-DMSO	

TD-DFT geometry optimisation: **singlets**; root = 1; nstates = 10			
Multiplicity 1; charge 0			
Energy: -1368.96303049			
C	3.51958600	2.54596000	-0.00332500
C	2.82761200	1.26126800	-0.00400800
N	1.47315200	1.47529600	0.00001400
C	1.26281900	2.82861500	0.00394500
C	2.54761800	3.51896200	0.00323400
C	0.00000000	3.44800600	0.00657900
C	-1.26281900	2.82861500	0.00394500
N	-1.47315200	1.47529600	0.00001400
C	-2.82761200	1.26126800	-0.00400800
C	-3.51958600	2.54596000	-0.00332500
C	-2.54761800	3.51896200	0.00323400
C	3.44246300	0.00000000	-0.00662300
C	2.82761200	-1.26126800	-0.00400800
C	3.51958600	-2.54596000	-0.00332500
C	2.54761800	-3.51896200	0.00323400
C	1.26281900	-2.82861500	0.00394500
N	1.47315200	-1.47529600	0.00001400
C	0.00000000	-3.44800600	0.00657900
C	-1.26281900	-2.82861500	0.00394500
C	-2.54761800	-3.51896200	0.00323400
C	-3.51958600	-2.54596000	-0.00332500
C	-2.82761200	-1.26126800	-0.00400800
N	-1.47315200	-1.47529600	0.00001400
C	-3.44246300	0.00000000	-0.00662300
Zn	0.00000000	0.00000000	0.00007100
H	0.00000000	-4.53187700	0.00951100
H	4.52669400	0.00000000	-0.00971100
H	0.00000000	4.53187700	0.00951100
H	-4.52669400	0.00000000	-0.00971100
C	2.71017500	5.00440100	0.00855500
H	2.23066500	5.45921600	0.88235400
H	2.25251700	5.46397100	-0.87459600
H	3.76211400	5.29397900	0.02271000
C	5.00459200	2.70917500	-0.00852000

H	5.45915200	2.22923600	-0.88190900
H	5.46354200	2.25225000	0.87504000
H	5.29371500	3.76118400	-0.02328100
C	5.00459200	-2.70917500	-0.00852000
H	5.46354200	-2.25225000	0.87504000
H	5.45915200	-2.22923600	-0.88190900
H	5.29371500	-3.76118400	-0.02328100
C	2.71017500	-5.00440100	0.00855500
H	2.25251700	-5.46397100	-0.87459600
H	2.23066500	-5.45921600	0.88235400
H	3.76211400	-5.29397900	0.02271000
C	-2.71017500	-5.00440100	0.00855500
H	-2.23066500	-5.45921600	0.88235400
H	-2.25251700	-5.46397100	-0.87459600
H	-3.76211400	-5.29397900	0.02271000
C	-5.00459200	-2.70917500	-0.00852000
H	-5.45915200	-2.22923600	-0.88190900
H	-5.46354200	-2.25225000	0.87504000
H	-5.29371500	-3.76118400	-0.02328100
C	-5.00459200	2.70917500	-0.00852000
H	-5.46354200	2.25225000	0.87504000
H	-5.45915200	2.22923600	-0.88190900
H	-5.29371500	3.76118400	-0.02328100
C	-2.71017500	5.00440100	0.00855500
H	-2.25251700	5.46397100	-0.87459600
H	-2.23066500	5.45921600	0.88235400
H	-3.76211400	5.29397900	0.02271000

Optimised coordinates for T_2 state ZnOEP	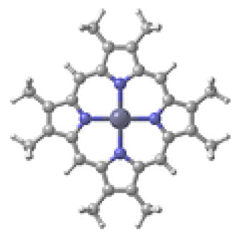
**T_2 state ZnOEP (TD-DFT)**	
Experimental compound: zinc octaethyl porphyrin	
Simplified for computation: zinc octamethyl porphyrin	
B3LYP-GD3BJ; 6-311G* (C H N), LanL2TZ (Zn); solvation SMD-DMSO	
TD-DFT geometry optimisation: **triplets**; root = 3; nstates = 10	

Multiplicity 1; charge 0			
Energy: -1368.97472320			
C	3.520476	2.546030	-0.003245
C	2.829663	1.263656	-0.003956
N	1.475673	1.476455	-0.000028
C	1.264696	2.829065	0.003946
C	2.547364	3.519192	0.003250
C	0.000000	3.445471	0.006586
C	-1.264696	2.829065	0.003946
N	-1.475673	1.476455	-0.000028
C	-2.829663	1.263656	-0.003956
C	-3.520476	2.546030	-0.003245
C	-2.547364	3.519192	0.003250
C	3.445189	0.000000	-0.006555
C	2.829663	-1.263656	-0.003956
C	3.520476	-2.546030	-0.003245
C	2.547364	-3.519192	0.003250
C	1.264696	-2.829065	0.003946
N	1.475673	-1.476455	-0.000028
C	0.000000	-3.445471	0.006586
C	-1.264696	-2.829065	0.003946
C	-2.547364	-3.519192	0.003250
C	-3.520476	-2.546030	-0.003245
C	-2.829663	-1.263656	-0.003956
N	-1.475673	-1.476455	-0.000028
C	-3.445189	-0.000000	-0.006555
Zn	0.000000	0.000000	-0.000048
H	0.000000	-4.529602	0.009674
H	4.529297	0.000000	-0.009552
H	-0.000000	4.529602	0.009674
H	-4.529297	-0.000000	-0.009552
C	2.711950	5.004911	0.008457
H	2.233392	5.460334	0.882170
H	2.255688	5.464794	-0.874966
H	3.764332	5.292915	0.022815
C	5.006019	2.709883	-0.008411
H	5.461340	2.231041	-0.882028
H	5.465747	2.253722	0.875155

H	5.294428	3.762166	-0.022990
C	5.006019	-2.709883	-0.008411
H	5.465747	-2.253722	0.875155
H	5.461340	-2.231041	-0.882028
H	5.294428	-3.762166	-0.022990
C	2.711950	-5.004911	0.008457
H	2.255688	-5.464794	-0.874966
H	2.233392	-5.460334	0.882170
H	3.764332	-5.292915	0.022815
C	-2.711950	-5.004911	0.008457
H	-2.233392	-5.460334	0.882170
H	-2.255688	-5.464794	-0.874966
H	-3.764332	-5.292915	0.022815
C	-5.006019	-2.709883	-0.008411
H	-5.461340	-2.231041	-0.882028
H	-5.465747	-2.253722	0.875155
H	-5.294428	-3.762166	-0.022990
C	-5.006019	2.709883	-0.008411
H	-5.465747	2.253722	0.875155
H	-5.461340	2.231041	-0.882028
H	-5.294428	3.762166	-0.022990
C	-2.711950	5.004911	0.008457
H	-2.255688	5.464794	-0.874966
H	-2.233392	5.460334	0.882170
H	-3.764332	5.292915	0.022815

13.4 Thermodynamics calculations

To evaluate the thermodynamics of each electron-transfer catalytic process, the Gibbs free energy change (ΔG) was calculated as $\Delta G = G(P_1) + G(P_2) - G(R_1) – G(R_2)$, where $G(P_1)$ and $G(P_2)$ represent the Gibbs free energies (G) of products while $G(R_1)$ and $G(R_2)$ represent those of reactants. G of each species was taken as the electronic energy as common practice, since this is sufficiently accurate for thermodynamic studies of electron transfer events. ΔG was converted from the atomic unit (a. u.) to kcal/mol for evaluation.

13.5 Probing into the $O_2^{\bullet -}$-DTPA pair

Geometry optimisation on the bimolecular system $O_2^{\bullet -}$-DTPA (before SET) with an overall multiplicity of 2 and a charge of -1, as well as the bimolecular system DTPA$^{\bullet -}$-O_2 (after SET) with an overall multiplicity of 4 and a charge of -1 was performed at B3LYP-GD3BJ/6-311+G** (SMD-DMSO) level of theory by the Gaussian D09 software. The highest SOMO of both systems with contains the outermost single electron of both systems were analysed by the Multiwfn software package[10] and visualised by VMD[3] (Supplementary Figure 40a and S40b). Reduced Density Gradient (RDG) analysis was then performed by the Multiwfn software package[10] to visualise weak interactions and reveal the strong interaction between $O_2^{\bullet -}$ and DTPA by observing the shoe-pad-shaped isosurface region between DTPA and $O_2^{\bullet -}$ (Supplementary Figure 40c).

<u>Molecular Coordinates:</u>

Optimised coordinates for the bimolecular system $O_2^{\bullet -}$-DTPA (before SET)				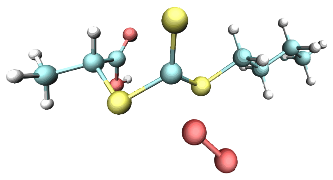
**$O_2^{\bullet -}$-DTPA, multiplicity = 2**				
B3LYP-GD3BJ; 6-311+G**; solvation SMD-DMSO				
Multiplicity 2; charge -1				
C	2.29828000	-1.87296000	-0.00683700	
C	2.90720500	-0.53269100	0.33669200	
O	1.74770000	-2.59764500	0.79150900	
O	2.46007600	-2.21696500	-1.30028100	
C	4.39940000	-0.47796000	0.00341000	
S	2.05316000	0.85913600	-0.53324500	
C	0.45078000	0.89392800	0.33218900	
S	-0.72030000	-0.00920100	-0.61821800	
S	0.35500300	1.17084300	1.99749900	
C	-2.24048700	0.00462700	0.40198300	
C	-3.37791300	-0.67081800	-0.35969400	
C	-4.66748000	-0.70867300	0.46617400	
C	-5.82149500	-1.37984600	-0.27806300	
H	2.74452400	-0.37544300	1.40155700	

H	2.04242300	-3.08493400	-1.42951500
H	4.82958100	0.45193600	0.37985700
H	4.91663800	-1.31414200	0.48239000
H	4.57689700	-0.53799700	-1.07198500
H	-2.02478700	-0.51504900	1.33643600
H	-2.48207800	1.04269800	0.63401400
H	-3.56503700	-0.13807100	-1.29872300
H	-3.09098100	-1.69448900	-0.62703400
H	-4.47683600	-1.23925400	1.40614700
H	-4.94999500	0.31497800	0.73802300
H	-6.73156700	-1.39404900	0.32909500
H	-6.05005000	-0.85141000	-1.20949200
H	-5.57533700	-2.41546600	-0.53484800
O	0.14290500	2.85902300	-0.48174000
O	-0.70804800	2.94116300	-1.44044300

Optimised coordinates for the bimolecular system $DTPA^{\bullet -}$-O_2 (after SET)

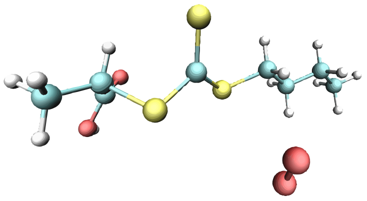

$DTPA^{\bullet -}$-O_2, multiplicity = 4

B3LYP-GD3BJ; 6-311+G**; solvation SMD-DMSO

Multiplicity 4; charge -1

C	3.35460800	-1.30602800	-0.58517300
C	3.22750900	-0.12272400	0.31991000
O	2.90886500	-2.41239200	-0.35436400
O	4.00761600	-1.02984500	-1.74096400
C	4.43385100	0.80441000	0.34818500
S	1.69575600	0.85194200	-0.22850600
C	0.36653900	0.13653700	0.67096000
S	-0.77526700	-0.75841800	-0.37556400
S	0.32552500	0.08588300	2.39095000
C	-2.39037500	-0.49940900	0.46281900
C	-3.53301700	-0.97001700	-0.43160100
C	-4.89752000	-0.80216800	0.24446100
C	-6.05540500	-1.25058800	-0.64719800
H	2.97076200	-0.48117700	1.31556800
H	3.99846800	-1.83830200	-2.27943900

H	4.28373400	1.57857700	1.10321500
H	5.33835000	0.24414500	0.60414300
H	4.59867600	1.28890100	-0.61643200
H	-2.38304700	-1.04508500	1.40721900
H	-2.48106200	0.56566800	0.68570000
H	-3.52313200	-0.40570900	-1.37102800
H	-3.38936200	-2.02471800	-0.69499500
H	-4.90960600	-1.37447500	1.17926800
H	-5.03382400	0.24964900	0.52211700
H	-7.01862900	-1.12131900	-0.14447700
H	-6.08382400	-0.67183800	-1.57638100
H	-5.95884200	-2.30752900	-0.91670800
O	-1.35995500	3.73913000	-0.28168800
O	-1.47927900	3.26968500	-1.38573700

Supplementary Note 14. Chain of evidence summarised for the O-RQP scheme

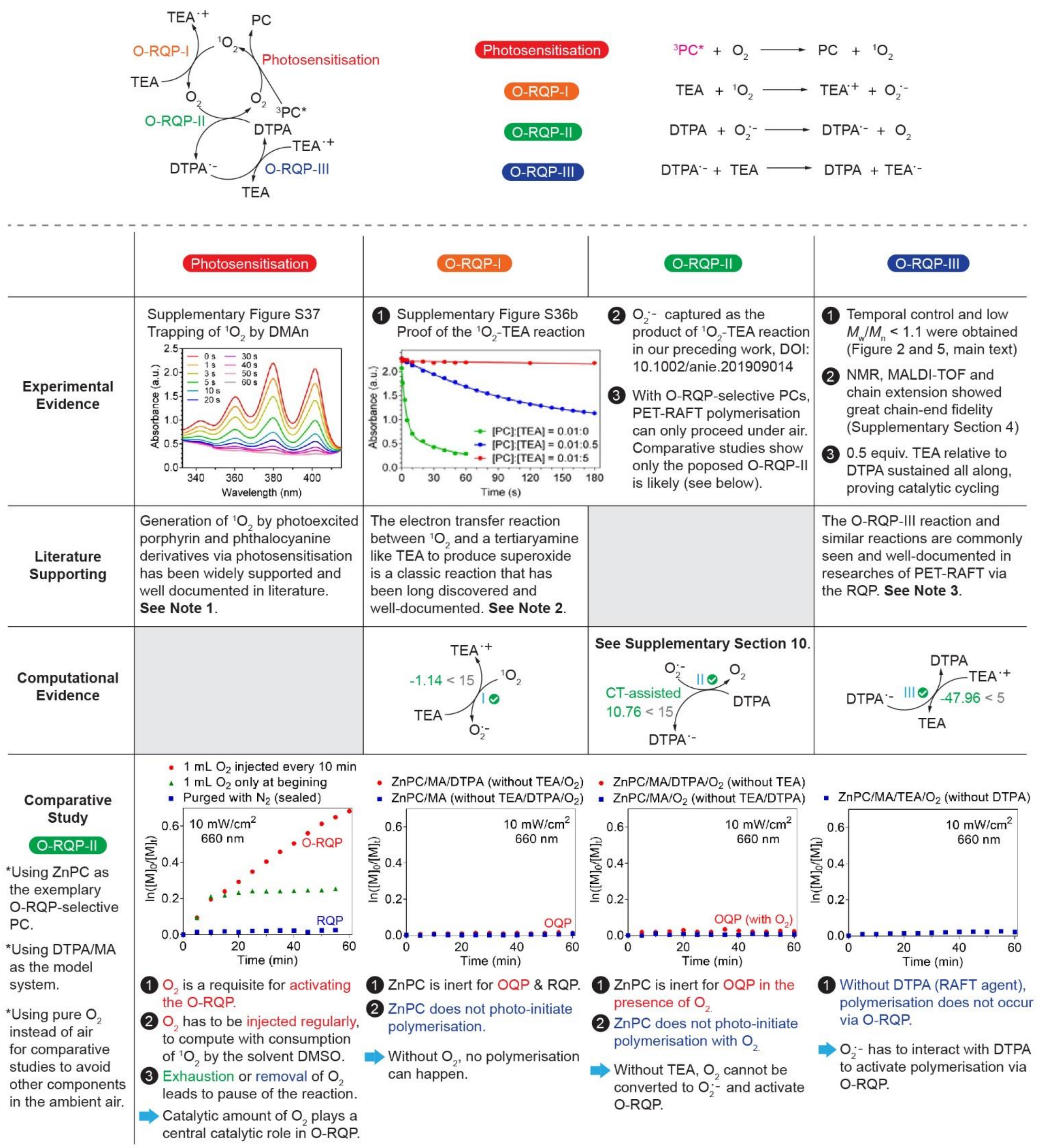

Supplementary Figure 46 | Chain of evidence and supporting materials for the O-RQP.

Note 1: Porphyrin and phthalocyanine derivatives are commonly used as $^1O_2$ photosensitisers because of their significant triplet quantum yields; their ability to generate $^1O_2$ is well-documented in the literature.[40-44]

Note 2: The electron transfer reaction between $^1O_2$ and a tertiary amine such as TEA producing superoxide $O_2^{\bullet -}$ is a classic reaction which has been studied and well-documented.[45-49]

Note 3: The O-RQP-III reaction and similar reactions are commonly seen and well-documented in work pertaining PET-RAFT via the RQP and other photoredox catalysed polymerisation techniques via the RQP.[50-52]

Supplementary References

1 Gruendling, T., Junkers, T., Guilhaus, M. & Barner-Kowollik, C. Mark-Houwink Parameters for the Universal Calibration of Acrylate, Methacrylate and Vinyl Acetate Polymers Determined by Online Size-Exclusion Chromatography-Mass Spectrometry. *Macromolecular Chemistry and Physics* **211**, 520-528, doi:10.1002/macp.200900323 (2010).

2 Lu, T. & Chen, F. Multiwfn: A multifunctional wavefunction analyzer. *Journal of Computational Chemistry* **33**, 580-592, doi:doi:10.1002/jcc.22885 (2012).

3 Humphrey, W., Dalke, A. & Schulten, K. VMD: visual molecular dynamics. *J Mol Graph* **14**, 33-38, 27-38, doi:10.1016/0263-7855(96)00018-5 (1996).

4 Wu, C. *et al.* Computer-Guided Discovery of a pH-Responsive Organic Photocatalyst and Application for pH and Light Dual-Gated Polymerization. *J Am Chem Soc* **141**, 8207-8220, doi:10.1021/jacs.9b01096 (2019).

5 Romero, N. A. & Nicewicz, D. A. Organic Photoredox Catalysis. *Chem Rev* **116**, 10075-10166, doi:10.1021/acs.chemrev.6b00057 (2016).

6 Sartor, S. M., McCarthy, B. G., Pearson, R. M., Miyake, G. M. & Damrauer, N. H. Exploiting Charge-Transfer States for Maximizing Intersystem Crossing Yields in Organic Photoredox Catalysts. *J Am Chem Soc* **140**, 4778-4781, doi:10.1021/jacs.8b01001 (2018).

7 Wu, C. *et al.* Guiding the Design of Organic Photocatalyst for PET-RAFT Polymerization: Halogenated Xanthene Dyes. *Macromolecules* **52**, 236-248, doi:10.1021/acs.macromol.8b02517 (2018).

8 Orr-Ewing, A. J. Perspective: How can ultrafast laser spectroscopy inform the design of new organic photoredox catalysts for chemical and materials synthesis? *Structural Dynamics* **6**, 010901, doi:10.1063/1.5082620 (2019).

9 Speckmeier, E., Fischer, T. G. & Zeitler, K. A Toolbox Approach To Construct Broadly Applicable Metal-Free Catalysts for Photoredox Chemistry: Deliberate Tuning of Redox Potentials and Importance of Halogens in Donor-Acceptor Cyanoarenes. *J Am Chem Soc* **140**, 15353-15365, doi:10.1021/jacs.8b08933 (2018).

10 Lu, T. & Chen, F. Multiwfn: a multifunctional wavefunction analyzer. *J Comput Chem* **33**, 580-592, doi:10.1002/jcc.22885 (2012).

11 Banfi, S. *et al.* Porphyrins and azaporphines as catalysts in alkene epoxidations with peracetic acid. *Tetrahedron Lett* **36**, 2317-2320, doi:https://doi.org/10.1016/0040-4039(95)00244-7 (1995).

12 Theriot, J. C. *et al.* Organocatalyzed atom transfer radical polymerization driven by visible light. *Science* **352**, 1082-1086, doi:10.1126/science.aaf3935 (2016).

13 Goulard, R., Scala, S. M. & Thomas, R. N. *Radiative Energy Transfer: Proceedings of the Symposium on Interdisciplinary Aspects of Radiative Energy Transfer*. (Elsevier Science, 2017).

14 Sahoo, H. Förster resonance energy transfer – A spectroscopic nanoruler: Principle and applications. *Journal of Photochemistry and Photobiology C: Photochemistry Reviews* **12**, 20-30, doi:https://doi.org/10.1016/j.jphotochemrev.2011.05.001 (2011).

15 Jang, S., Newton, M. D. & Silbey, R. J. Multichromophoric Forster resonance energy transfer. *Phys Rev Lett* **92**, 218301, doi:10.1103/PhysRevLett.92.218301 (2004).

16 Balzani, V., Bolletta, F. & Scandola, F. Vertical and "nonvertical" energy transfer processes. A general classical treatment. *J Am Chem Soc* **102**, 2152-2163, doi:10.1021/ja00527a002 (1980).

17 Bai, S., Zhang, P., Antoniou, P., Skourtis, S. S. & Beratan, David N. Quantum interferences among Dexter energy transfer pathways. *Faraday Discussions* **216**, 301-318, doi:10.1039/C9FD00007K (2019).

18 Marcus, R. A. On the Theory of Oxidation‐Reduction Reactions Involving Electron Transfer. I. *The Journal of Chemical Physics* **24**, 966-978, doi:10.1063/1.1742723 (1956).

19 Marcus, R. A. Electrostatic Free Energy and Other Properties of States Having Nonequilibrium Polarization. I. *The Journal of Chemical Physics* **24**, 979-989, doi:10.1063/1.1742724 (1956).

20 Marcus, R. A. On the Theory of Oxidation‐Reduction Reactions Involving Electron Transfer. III. Applications to Data on the Rates of Organic Redox Reactions. *The Journal of Chemical Physics* **26**, 872-877, doi:10.1063/1.1743424 (1957).

21 Marcus, R. A. On the Theory of Oxidation—Reduction Reactions Involving Electron Transfer. V. Comparison and Properties of Electrochemical and Chemical Rate Constants1. *The Journal of Physical Chemistry* **67**, 853-857, doi:10.1021/j100798a033 (1963).

22 Marcus, R. A. Chemical and Electrochemical Electron-Transfer Theory. *Annual Review of Physical Chemistry* **15**, 155-196, doi:10.1146/annurev.pc.15.100164.001103 (1964).

23 Corrigan, N., Xu, J., Boyer, C. & Allonas, X. Exploration of the PET-RAFT Initiation Mechanism for Two Commonly Used Photocatalysts. *ChemPhotoChem* **0**, doi:10.1002/cptc.201800182 (2019).

24 Seal, P., Xu, J., De Luca, S., Boyer, C. & Smith, S. C. Unraveling Photocatalytic Mechanism and Selectivity in PET-RAFT Polymerization. *Advanced Theory and Simulations* **2**, 1900038, doi:10.1002/adts.201900038 (2019).

25 Xu, J., Jung, K., Atme, A., Shanmugam, S. & Boyer, C. A robust and versatile photoinduced living polymerization of conjugated and unconjugated monomers and its oxygen tolerance. *J Am Chem Soc* **136**, 5508-5519, doi:10.1021/ja501745g (2014).

26 Xu, J. T., Jung, K., Corrigan, N. A. & Boyer, C. Aqueous photoinduced living/controlled polymerization: tailoring for bioconjugation. *Chemical Science* **5**, 3568-3575, doi:10.1039/c4sc01309c (2014).

27 Brewer, L. The Determination of Stability Constants and Other Equilibrium Constants in Solution. Francis J. C. Rossotti and Hazel Rossotti. McGraw-Hill, New York, 1961. xiv + 425 pp. $12.50. *Science* **136**, 643-644, doi:10.1126/science.136.3516.643 (1962).

28 Rossotti, F. J. C. & Rossotti, H. *The determination of stability constants: and other equilibrium constants in solution*. (McGraw-Hill, 1961).

29 Physical, U. i. d. c. p. e. a. *et al. Quantities, Units and Symbols in Physical Chemistry*. (Royal Society of Chemistry, 2007).

30 Bard, A. J., Inzelt, G. & Scholz, F. *Electrochemical Dictionary*. (Springer Berlin Heidelberg, 2012).

31 Stock, J. T., Chemistry, A. C. S. D. o. t. H. o., Orna, M. V., Chemistry, A. C. S. D. o. A. & Meeting, A. C. S. *Electrochemistry: past and present*. (American Chemical Society, 1989).

32 Mariano, P. S. *Advances in Electron Transfer Chemistry*. (Elsevier Science, 2013).

33 Silverstein, T. P. Marcus Theory: Thermodynamics CAN Control the Kinetics of Electron Transfer Reactions. *Journal of Chemical Education* **89**, 1159-1167, doi:10.1021/ed1007712 (2012).

34 Arrhenius, S. in *Zeitschrift für Physikalische Chemie* Vol. 4U 226 (1889).

35 Eyring, H. The Activated Complex in Chemical Reactions. *The Journal of Chemical Physics* **3**, 107-115, doi:10.1063/1.1749604 (1935).

36 Laidler, K. J. & King, M. C. Development of transition-state theory. *The Journal of Physical Chemistry* **87**, 2657-2664, doi:10.1021/j100238a002 (1983).

37 Pineiro, M. *et al.* Photoacoustic Measurements of Porphyrin Triplet-State Quantum Yields and Singlet-Oxygen Efficiencies. *Chemistry – A European Journal* **4**, 2299-2307, doi:10.1002/(sici)1521-3765(19981102)4:11<2299::aid-chem2299>3.0.co;2-h (1998).

38 Redmond, R. W. & Kochevar, I. E. Symposium-in-Print: Singlet Oxygen Invited Review. *Photochem Photobiol* **82**, 1178-1186, doi:10.1562/2006-04-14-IR-874 (2006).

39 Minaev, B. F. Electronic mechanisms of activation of molecular oxygen. *Russian Chemical Reviews* **76**, 1059-1083, doi:10.1070/RC2007v076n11ABEH003720 (2007).

40 Rybicka-Jasinska, K., Shan, W., Zawada, K., Kadish, K. M. & Gryko, D. Porphyrins as Photoredox Catalysts: Experimental and Theoretical Studies. *J Am Chem Soc* **138**, 15451-15458, doi:10.1021/jacs.6b09036 (2016).

41 Alberto, M. E., De Simone, B. C., Mazzone, G., Sicilia, E. & Russo, N. The heavy atom effect on Zn(ii) phthalocyanine derivatives: a theoretical exploration of the photophysical properties. *Phys Chem Chem Phys* **17**, 23595-23601, doi:10.1039/c5cp03833b (2015).

42 Josefsen, L. B. & Boyle, R. W. Photodynamic therapy and the development of metal-based photosensitisers. *Met Based Drugs* **2008**, 276109, doi:10.1155/2008/276109 (2008).

43 Yeow, J. *et al.* A Polymerization-Induced Self-Assembly Approach to Nanoparticles Loaded with Singlet Oxygen Generators. *Macromolecules* **49**, 7277-7285, doi:10.1021/acs.macromol.6b01581 (2016).

44 Zhang, X. F. & Xu, H. J. Influence of Halogenation and Aggregation on Photosensitizing Properties of Zinc Phthalocyanine (Znpc). *J Chem Soc Faraday T* **89**, 3347-3351, doi:DOI 10.1039/ft9938903347 (1993).

45 Smith, W. F. Kinetic Evidence for Both Quenching and Reaction of Singlet Oxygen with Triethylamine in Pyridine Solution. *Journal of the American Chemical Society* **94**, 186-&, doi:DOI 10.1021/ja00756a032 (1972).

46 Tsubomura, H., Yagishita, T. & Toi, H. Intermediates and Mechanism of Photo-Oxygenation Reaction of Triethylamine. *Bulletin of the Chemical Society of Japan* **46**, 3051-3055, doi:10.1246/bcsj.46.3051 (1973).

47 Haugen, C. M., Bergmark, W. R. & Whitten, D. G. Singlet Oxygen Mediated Fragmentation of Amino-Alcohols, 1,2-Diamines, and Amino Ketones. *Journal of the American Chemical Society* **114**, 10293-10297, doi:DOI 10.1021/ja00052a027 (1992).

48 Bernstein, R. & Foote, C. S. Singlet Oxygen Involvement in the Photochemical Reaction of C60and Amines. Synthesis of an Alkyne-Containing Fullerene. *The Journal of Physical Chemistry A* **103**, 7244-7247, doi:10.1021/jp991534t (1999).

49 Cocquet, G., Rool, P. & Ferroud, C. Photosensitized oxidation, by single-electron transfer, of catharanthine and vindoline: a highly regio- and diastereoselective photocyanation reaction. *J Chem Soc Perk T 1*, 2277-2281, doi:10.1039/b001114m (2000).

50 Corrigan, N., Xu, J. T. & Boyer, C. A Photoinitiation System for Conventional and Controlled Radical Polymerization at Visible and NIR Wavelengths. *Macromolecules* **49**, 3274-3285, doi:10.1021/acs.macromol.6b00542 (2016).

51 Liu, X. D., Zhang, L. F., Cheng, Z. P. & Zhu, X. L. Metal-free photoinduced electron transfer-atom transfer radical polymerization (PET-ATRP) via a visible light organic photocatalyst. *Polym Chem-Uk* **7**, 689-700, doi:10.1039/c5py01765c (2016).

52 Xu, J. T., Shanmugam, S., Duong, H. T. & Boyer, C. Organo-photocatalysts for photoinduced electron transfer-reversible addition-fragmentation chain transfer (PET-RAFT) polymerization. *Polym Chem-Uk* **6**, 5615-5624, doi:10.1039/c4py01317d (2015).
